# Design, Synthesis, *In Vitro* Biological Evaluation, and *In Silico* Analyses of Novel Pyrano[3,4‑*b*]indole-Containing Compounds as Promising Multitarget Candidates for Alzheimer’s Disease and Cancer Therapy

**DOI:** 10.1021/acsomega.6c03670

**Published:** 2026-08-26

**Authors:** Ferah Comert Onder, Alper Onder, Kadircan Ural, Merve Sıkık, Khaled A.N. Abusharkh, Bulent Ozpolat, Mehmet Ay

**Affiliations:** † 52950 Çanakkale Onsekiz Mart University, Faculty of Medicine, Department of Medical Biology, Çanakkale 17020, Türkiye; ‡ 52950 Çanakkale Onsekiz Mart University, Faculty of Science, Department of Chemistry, Natural Products and Drug Research Laboratory, Çanakkale 17020, Türkiye; § 52950 Çanakkale Onsekiz Mart University, School of Graduate Studies, Department of Medical System Biology, Çanakkale 17020, Türkiye; ∥ Al-Quds University, Faculty of Science and Technology, Department of Chemistry and Chemical Technology, East Jerusalem 20002, Palestine; ⊥ 6187 The University of Oklahoma, Stephenson School of Biomedical Engineering, Norman, Oklahoma 73019, United States

## Abstract

Alzheimer’s disease (AD) is a neurodegenerative disease associated with decreased activity of the cholinergic system in the brain. Identifying a drug that has no side effects and can prevent or delay the progression of this neurodegenerative disease is crucial. In this study, 11 novel pyrano­[3,4-*b*]­indole derivatives (**ECA-ECM**) were designed and synthesized as AChE and BChE inhibitors, and their structures were elucidated by spectroscopic analyses. Enzyme activity studies were conducted *in vitro*. Inhibitory potentials for both cholinesterase enzymes were determined by *in silico* analyses, and protein–ligand interactions were elucidated. The **ECB** compound (AChE IC_50_ = 35.31 ± 0.81 nM and BChE IC_50_ = 28.91 ± 0.19 nM) is a potential candidate as a dual AChE/BChE inhibitor. These findings indicate that the synthesized pyrano­[3,4-*b*]­indole derivatives have inhibitory potential for AD. The cytotoxic activities of the synthesized compounds were evaluated *in vitro*. The **ECK** compound showed a strong cytotoxic effect in five different human cancer cell lines in the ranging concentrations from 10.37 ± 1.43 to 29.34 ± 1.71 μM. The **ECD** compound exhibited strong cytotoxic activity at a concentration of 9.27 ± 0.97 μM in the colon cancer cell line. The cytotoxic activities of **ECK** and **ECM** were found to be below 25 μM in four human cancer cell lines. *In silico* analyses, including molecular docking, molecular dynamics simulations, and binding free energy calculations, confirmed that the synthesized compounds are potential candidates targeting AChE and BChE. Consequently, the synthesized compounds showed promising results for the treatment of AD and cancer.

## Graphical abstract

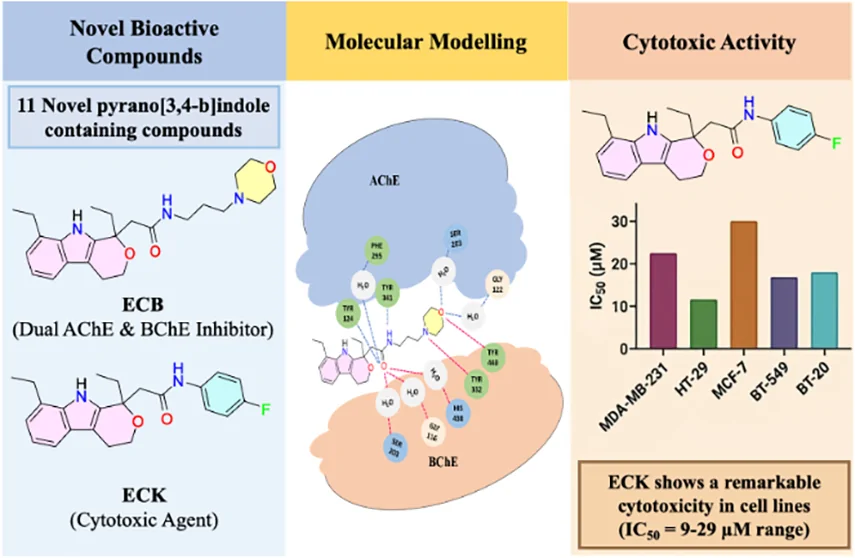

## 1 Introduction

Alzheimer’s disease (AD) is a neurodegenerative disorder affecting millions of people worldwide, characterized by progressive cognitive decline, memory loss, and behavioral changes.^1,2^ Statistics, indicate that approximately 50 million people worldwide are affected by AD or related dementias, highlighting the increasing prevalence of the condition.^3^ The pathogenesis of AD involves the neurotoxic accumulation of extracellular amyloid-β plaques and intracellular tau neurofibrillary tangles, contributing to neuronal loss and cognitive impairment.^4^ Additionally, genetic factors, environmental influences, and aging play significant roles in initiating the pathological changes observed in AD, such as neuron loss, synaptic insufficiency, and the formation of β-amyloid plaques and neurofibrillary tangles.^5^ The cholinergic hypothesis, in particular, suggests that the dysfunction of cholinergic neurons plays a crucial role in the cognitive decline associated with AD.^6^ This hypothesis highlights the importance of acetylcholinesterase (AChE) inhibition in raising acetylcholine (ACh) levels and enhancing cholinergic transmission, thereby alleviating the associated cognitive symptoms of the disease.^7,8^ AChE is a key enzyme involved in the regulation of the neurotransmitter ACh in the central and peripheral nervous systems, various organs, and synapses.^7^ Enzymatic hydrolysis of ACh by AChE is essential for maintaining proper synaptic function and preventing the overstimulation of cholinergic receptors.^9^ Inhibition of AChE can maintain or increase ACh levels, leading to improved neuronal transmission and cognitive function in individuals with AD.^10,11^ Consequently, AChE remains a primary therapeutic target due to its role in ACh hydrolysis and its effect on cholinergic neurotransmission.^12,13^ Recent studies have shown a significant loss of presynaptic cholinergic neurons in the brains of individuals with AD, leading to the impaired cholinergic markers such as ACh and choline acetyltransferase (ChAT).^7,13^ The activity of AChE has been modified through various compounds and inhibitors. This modulation offers a promising avenue for developing new therapies targeting cholinergic dysfunction in neurodegenerative disorders.^8,14,15^

Tacrine was the first AChE inhibitor for the treatment of AD, followed by donepezil, galantamine, and rivastigmine, which subsequently entered clinical use.^16,17^ These drugs have shown clinical efficacy in patients with mild to moderate AD, but their therapeutic benefits are significantly limited by systemic side effects, such as muscle cramps and gastrointestinal discomfort, as well as ineffectiveness in advanced stages of the disease.^16,17^ Specifically, as AD progresses to advanced stages, AChE activity decreases to approximately 55%–67% of physiological levels, while butyrylcholinesterase (BChE) activity increases up to 120%.^18^ Consequently, BChE becomes the dominant enzyme responsible for ACh hydrolysis in the brain during late-stage AD, highlighting the clinical necessity of developing potent and selective or dual inhibitors. In conclusion, selective inhibition of BChE represents a strategically viable and potentially safer therapeutic intervention, particularly for mitigating cholinergic deficits and restoring cognitive performance in patients with advanced AD.^18^

Etodolac (1,8-diethyl-1,3,4,9-tetrahydropyrano­[3,4-*b*]­indol-1-yl)­acetic acid) is a nonsteroidal anti-inflammatory drug (NSAID) commonly used to relieve pain and reduce inflammation in conditions such as arthritis.^19^ It acts by inhibiting cyclooxygenase enzymes, which play a role in the production of prostaglandins that contribute to pain and inflammation.^20^ Recent studies have investigated the neuroprotective properties of etodolac and its derivatives in preclinical models of AD and highlighted their potential as novel treatment options for this debilitating condition.^21^ Furthermore, the studies have investigated the impact of etodolac on oxidative stress, neuroinflammation, and neuronal function in the context of AD pathology.^22,23^ It has also been examined for its potential antitumor properties in various cancer types. Recent studies collectively highlight the diverse potential of etodolac in targeting various cancer types through mechanisms such as apoptosis induction and inhibition of cancer cell proliferation. In the field of targeted cancer therapy, novel etodolac derivatives have been identified as eukaryotic elongation factor 2 kinase (eEF2K) inhibitors and have shown potential in precision medicine for cancer treatment.^24^ In general, indole-based compounds have been reported to be antineurodegenerative agents.^25–29^ Figure 1 shows the structures of some known drugs and candidates containing an indole scaffold. In recent years, Multiple Target Directed Ligands (MTDLs), a dual-target strategy, have gained significant interest due to common pathophysiological pathways such as oxidative stress, kinase dysregulation, and apoptotic signaling between neurodegenerative disorders and cancer.^30–35^ The present study investigates the anti-Alzheimer’s and anticancer potentials of the synthesized compounds to discover versatile scaffolds that can address these interconnected complex pathologies.

**Figure 1:**
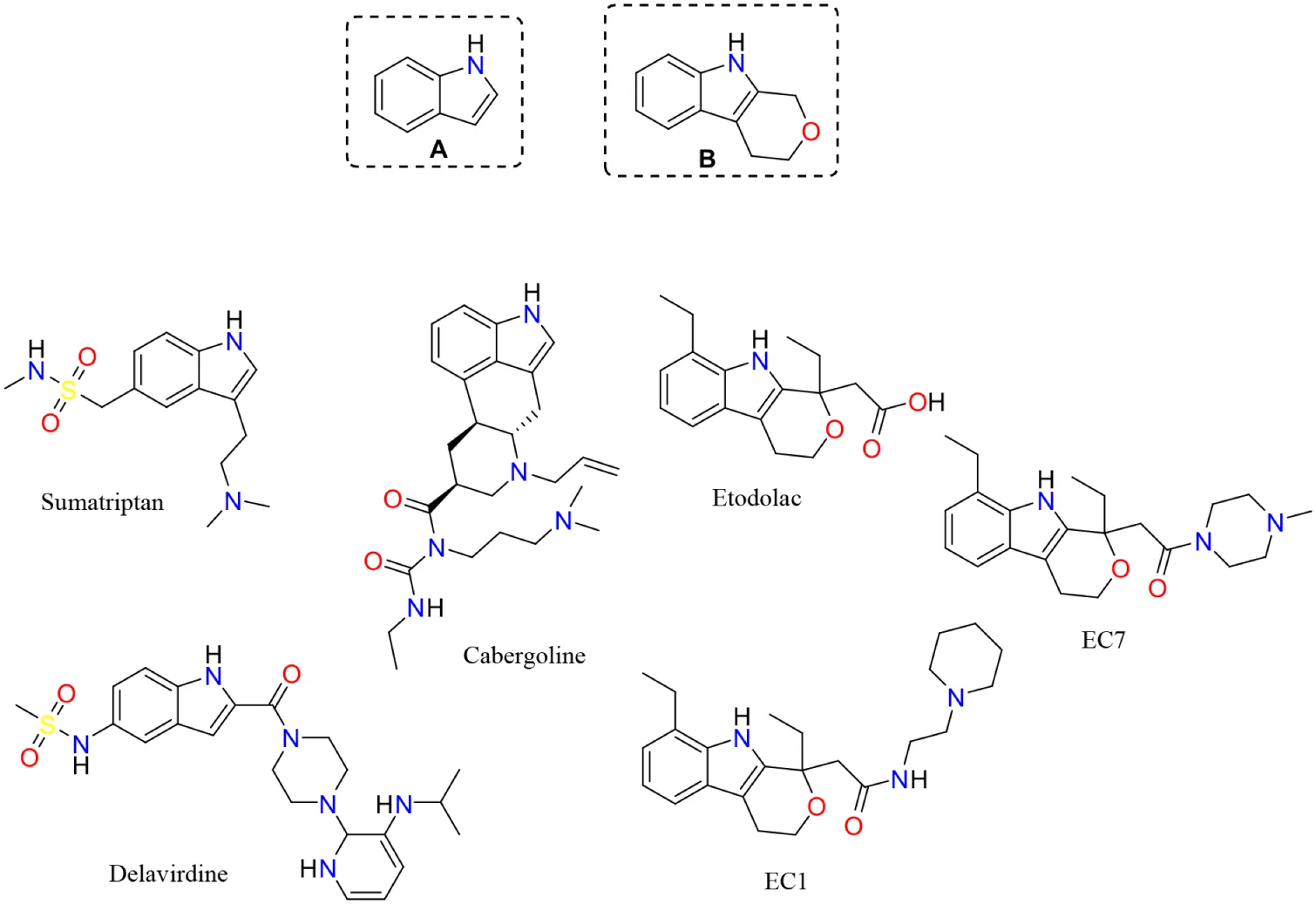
Structures of some known A) indole and B) pyrano­[3,4-*b*]­indole containing drugs and candidates.^24–28^

In conclusion, the aim of this study was to design, synthesize, and characterize novel and effective substituted pyrano­[3,4-*b*]­indole compounds (**ECA-ECM**) as potential cholinesterase inhibitors (AChE and BChE), determine their *in vitro* enzyme-activities, and conduct *in silico* studies investigating their ligand binding capabilities and protein–ligand interactions using molecular docking, molecular dynamics (MD) simulation, and binding free energy analyses. Moreover, the anticancer activity of the synthesized novel compounds was investigated in various breast and colon cancer cell lines. The findings are promising for novel drug candidates and provide guidance for future studies targeting AD and cancers.

## 2 Results and Discussion

### 2.1 Synthesis

Figure 2 displays the overall reaction scheme and the structures of the 11 novel compounds synthesized. The structures of the 11 novel 1,8-diethyl-1,3,4,9-tetrahydropyrano­[3,4-*b*]­indole derivatives (**ECA-ECM**) were confirmed by comprehensive spectroscopic analyses, including Fourier-transform infrared (FT-IR), Proton nuclear magnetic resonance (^1^H NMR), Carbon-13 nuclear magnetic resonance (^13^C NMR), and mass spectrometry (MS) techniques. The FT-IR spectra of all compounds exhibited characteristic absorption bands, confirming the presence of the common functional groups. The N–H stretching vibrations appeared in the range of 3396–3278 cm^–1^, while aromatic C–H stretching was observed in the range of 3033–3109 cm^–1^. The aliphatic C–H stretching vibrations were detected at 2995–2838 cm^–1^, while carbonyl (C=O) stretching of the amide group were detected at 1706–1621 cm^–1^, consistent with amide functionality. Aromatic C= C stretching vibrations were observed in the range of 1594–1447 cm^–1^. The ^1^H NMR spectra provided definitive evidence for the proposed structures; the indole N–H proton appeared as a singlet at δ 9.46–10.57 ppm, while the amide N–H proton was observed between δ 7.59–10.45 ppm depending on the substituent. Aromatic protons were detected in the expected region (δ 6.67–8.19 ppm), while the characteristic methylene protons of the pyrano ring system appeared at δ 2.48–4.37 ppm. The ethyl substituents were confirmed with triplet signals at δ 0.62–0.87 ppm and δ 1.20–1.49 ppm with appropriate coupling constants (*J* = 7.2–7.5 Hz). The ^13^C NMR spectra further confirmed the structures, with carbonyl carbons resonating at δ 168.50–173.29 ppm, aromatic carbons in the δ 107.29–138.51 ppm range, and aliphatic carbons at δ 7.59–76.36 ppm. Due to the low yield (3%) of the isolated compound **ECC**, the ^13^C NMR and ^13^C NMR APT spectra have a relatively low signal-to-noise ratio. However, the fundamental signals, including carbonyl carbons at 170.94 and 170.95 ppm, and aromatic and aliphatic protons, are clearly distinguishable and fully support the proposed structure. MS analysis yielded molecular ion peaks consistent with the calculated molecular formulas; compounds **ECB, ECD, ECE, ECG**, **ECH, ECK,** and **ECM** analyzed by Liquid Chromatography Tandem Mass Spectrometry (LC-MS/MS) showed [M + 1]^+^ or [M – 1]^−^ peaks, while compounds **ECC, ECF**, and **ECL** were characterized by Quadrupole Time-of-Flight High-Resolution Mass Spectrometry (QTOF-HRMS) exhibited accurate mass measurements within acceptable error limits. Spectroscopic data collectively confirmed that all target compounds were successfully synthesized in high purity. The 3% isolate yield for compound **ECC** is mainly due to the structural characteristics and the chair conformation of the starting amine, 3-(piperidin-1-yl)­propan-1-amine, and the challenges encountered during isolation. Although 3-(piperidin-1-yl)­propan-1-amine is an aliphatic amine, the presence of flexible piperidin-1-yl alkyl chain may cause significant steric hindrance around the primary amino group. This conformational bulk reduces the rate of effective nucleophilic attack on the electrophilic center, leading to a slow conversion rate even over extended reaction time. Furthermore, preparative thin layer chromatography (prep TLC) was applied to obtain the target compound with high analytical purity, which is necessary for biological analyses.

**Figure 2:**
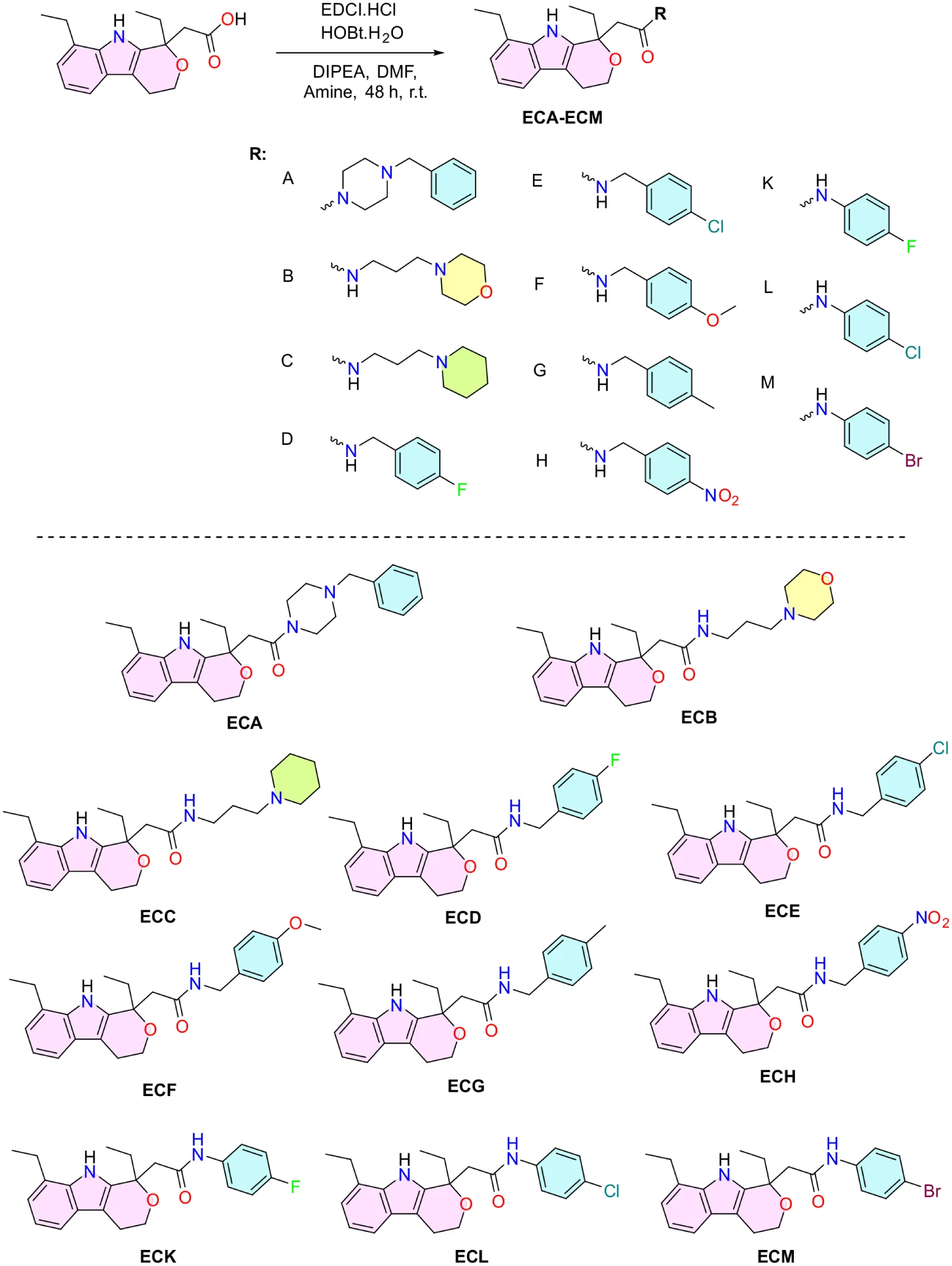
General reaction scheme and the structures of the 11 novel compounds synthesized.

### 2.2 *In Vitro* Enzyme Activity

The enzyme inhibitory activities of the synthesized substituted pyrano­[3,4-*b*]­indole derivatives (**ECA-ECM**) against the key enzymes AChE and BChE, which are targeted in the treatment of AD, were evaluated *in vitro*. The IC_50_ values of the synthesized compounds are given in Table 1. The obtained data were compared with the reference inhibitor tacrine. The IC_50_ values of the compounds targeting AChE were found to be between 35.31 nM and 80.02 nM. **ECB**, which has the lowest IC_50_ value (IC_50_ = 35.31 ± 0.81 nM), has the strongest inhibitory potential against the AChE enzyme; this is followed by **ECA** (IC_50_ = 45.03 ± 0.44 nM) and **ECC** (IC_50_ = 46.59 ± 0.68 nM), respectively. Among the synthesized compounds, **ECA, ECB**, and **ECC**, which had the lowest IC_50_ values, were found to be 2.14, 1.68, and 1.62 times more potent inhibitors, respectively, compared to the reference inhibitor tacrine (IC_50_ = 75.63 ± 0.77 nM). The synthesized compounds (**ECA-ECG, ECK-ECM**) were found to be more potent than the reference inhibitor tacrine (IC_50_ = 75.63 ± 0.77 nM) and were identified as potential AChE inhibitors. The **ECH** compound, with an IC_50_ value of 80.02 ± 0.60 nM, showed results close to tacrine for AChE. The AChE inhibition effects of groups attached to 2-(1,8-diethyl-1,3,4,9-tetrahydropyrano­[3,4-*b*]­indol-1-yl) were investigated as follows:

**Table 1:** AChE and BChE Enzyme Inhibition (IC_50_ nM) Values of the Synthesized Compounds^a^

No	Compounds	AChE (IC_50_ nM)	BChE (IC_50_ nM)
1	**ECA**	45.03 ± 0.44	37.24 ± 0.61
2	**ECB**	35.31 ± 0.81	28.91 ± 0.19
3	**ECC**	46.59 ± 0.68	37.45 ± 0.28
4	**ECD**	57.84 ± 0.49	37.06 ± 0.29
5	**ECE**	48.75 ± 0.56	36.78 ± 0.38
6	**ECF**	64.74 ± 1.93	37.06 ± 0.61
7	**ECG**	50.22 ± 1.12	28.95 ± 0.65
8	**ECH**	80.02 ± 0.60	37.33 ± 0.46
9	**ECK**	64.58 ± 0.30	35.19 ± 0.65
10	**ECL**	66.53 ± 1.08	34.73 ± 0.72
11	**ECM**	66.80 ± 0.38	33.80 ± 0.60
12	**Tacrine**	75.63 ± 0.77	45.47 ± 1.57

a SD (±) indicates the standard deviation.

*N*-(3-morpholinopropyl) > 1-(4-benzylpiperazin-1-yl) > *N*-(3-(piperidin-1-yl)­propyl) > *N*-(4-chlorobenzyl) > *N*-(4-methylbenzyl) > *N*-(4-fluorobenzyl) > *N*-(4-fluorophenyl) > *N*-(4-methoxybenzyl) > *N*-(4-chlorophenyl) > *N*-(4-bromophenyl) > *N*-(4-nitrobenzyl).

When the results obtained against BChE were evaluated, the IC_50_ values of the synthesized compounds were found to be between 28.95 nM and 37.45 nM. In particular, **ECB** (IC_50_ = 28.91 ± 0.19 nM) and **ECG** (IC_50_ = 28.95 ± 0.65 nM) stood out as the most potent inhibitors of BChE. These were followed by **ECM** (IC_50_ = 33.80 ± 0.60 nM) and **ECL** (IC_50_ = 34.73 ± 0.72 nM), respectively. The reference drug tacrine, with an IC_50_ value of 45.47 ± 1.57 nM, had a lower inhibitory potency than the synthesized compounds. **ECB** and **ECG** were found to be 1.57 times stronger BChE inhibitors than tacrine. The BChE inhibition effects of groups attached to 2-(1,8-diethyl-1,3,4,9-tetrahydropyrano­[3,4-*b*]­indol-1-yl) were investigated as follows:

*N*-(3-morpholinopropyl) > *N*-(4-methylbenzyl) > *N*-(4-bromophenyl) > *N*-(4-chlorophenyl) > *N*-(4-fluorophenyl) > *N*-(4-chlorobenzyl) > *N*-(4-fluorobenzyl) = *N*-(4-methoxybenzyl) > 1-(4-benzylpiperazin-1-yl) > *N*-(4-nitrobenzyl) > *N*-(3-(piperidin-1-yl)­propyl).

In conclusion, all synthesized compounds were found to be more potent than tacrine for BChE. Compound **ECB**, which had the lowest IC_50_ values against both enzymes, is a potential candidate as a dual enzyme inhibitor (AChE/BChE). These findings demonstrate that the synthesized substituted pyrano­[3,4-*b*]­indole derivatives have enzyme inhibitory potential for AD.

In our previously reported studies, the IC_50_ values of tacrine for AChE were found to be 77.41 ± 3.12 nM, 74.23 ± 0.83 nM and 68.46 ± 0.42 nM, and for BChE 45.43 ± 4.46 nM.^28,36–38^ Rivastigmine is a dual inhibitor of AChE and BChE and is known to have IC_50_ values at micromolar (μM) concentrations.^39^ Consequently, the newly synthesized pyrano­[3,4-*b*]­indole-based compounds were determined to be potential inhibitors of AChE and BChE at nanomolar concentrations.

### 2.3 Cytotoxic Activity

The anticancer activities of the 11 synthesized pyrano­[3,4-*b*]­indole derivatives were evaluated in five different human cancer cell lines using the MTT (3-(4,5-dimethylthiazol-2-yl)-2,5-diphenyltetrazolium bromide) assay. The obtained IC_50_ (μM) values are given in Table 2. Some of the synthesized compounds were found to exhibit significant cytotoxic effects, particularly in colon cancer cell lines. In the HT-29 cell line, the lowest IC_50_ value of the tested compounds was calculated for **ECD** (IC_50_ = 9.27 ± 0.97 μM). This was followed by **ECK** (IC_50_ = 10.37 ± 1.43 μM) and **ECM** (IC_50_ = 12.86 ± 1.71 μM), respectively. **ECK** was also found to be effective in BT-20, MCF-7, and BT-549 cell lines with the IC_50_ values below 30 μM. Compounds **ECG**, **ECL,** and **ECM** exhibited strong cytotoxicity in almost all cell lines, even at doses below 30 μM. Compounds **ECG**, **ECK, ECL,** and **ECM** showed strong cytotoxicity in the breast cancer cell line with the IC_50_ values ranging from 18–36 μM. In contrast, **ECD** was found to be less effective in the BT-20 cell line at ≥100 μM. Compound **ECK** (IC_50_ = 14.92 ± 7.05 μM) showed significant cytotoxicity in the BT-549 cell line. Compound **ECD** (IC_50_ = 53.38 ± 12.84 μM) was effective in the BT-549 cell line at lower doses. The IC_50_ value for **ECF** was calculated to be below 50 μM in four different cancer cell lines. The cytotoxicity values of the chemotherapy drug Doxorubicin (Dox) have been reported in several studies.^40–43^ For example, the IC_50_ value of Dox was found to be 87.7 nM.^40^ However, significantly higher IC_50_ values, such as 1.69 μM and 0.28 μM, have been reported in other studies on MDA-MB-231 cell lines.^41,42^ Furthermore, Dox concentration as high as 3 μM reduced the colonies of MDA-MB-231 cells.^43^ However, we reported the effect of Dox on the colony formation and cell proliferation in triple negative breast cancer (TNBC) cell lines.^44,45^ The 48 h cytotoxicity of Dox has been reported at a concentration of approximately 100 nM in MDA-MB-231.^45^

**Table 2:** Determination of the Cytotoxic Potential (IC_50_ μM) of Novel Compounds Using the MTT Assay

	Human cancer cell lines^a^				
Compounds	MDA-MB-231	BT-20	HT-29	MCF-7	BT-549
**ECA**	≥100	≥100	≥100	≥100	-
**ECB**	≥100	≥100	65.74 ± 5.17	≥100	≥100
**ECC**	72.85 ± 13.30	≥100	68.62 ± 4.79	≥100	-
**ECD**	43.11 ± 9.46	≥100	9.27 ± 0.97	78.05 ± 16.13	53.38 ± 12.84
**ECE**	≥100	≥100	≥100	≥100	≥100
**ECF**	48.59 ± 7.81	42.10 ± 5.37	49.56 ± 16.21	36.72 ± 5.68	-
**ECG**	29.13 ± 1.41	35.96 ± 2.60	15.36 ± 2.26	34.88 ± 6.19	87.80 ± 8.57
**ECH**	≥100	≥100	≥100	≥100	≥100
**ECK**	20.78 ± 2.79	22.35 ± 1.13	10.37 ± 1.43	29.34 ± 1.71	14.92 ± 7.05
**ECL**	23.29 ± 2.40	18.78 ± 2.98	25.78 ± 2.42	20.77 ± 2.62	-
**ECM**	23.28 ± 1.04	24.15 ± 0.91	12.86 ± 1.71	24.60 ± 3.08	≥100

a SD (±) indicates the standard deviation. (−) indicates that it has not been analyzed.

Among the synthesized compounds, **ECA, ECB**, **ECC, ECE**, and **ECH** did not show significant cytotoxic effects in various cancer cell lines, as their IC_50_ values were greater than 100 μM. MTT results revealed that **ECD, ECF, ECG**, **ECK, ECL,** and **ECM** exhibited significant cytotoxic activity at low doses in almost all cancer cell lines. It is of great importance that most of the synthesized compounds showed strong cytotoxic effects in breast and colon cancer cell lines. These results suggest that the anticancer activity of these compounds is promising for future studies to elucidate their molecular mechanisms *in vitro* and evaluate their potential in the treatment of breast, colon, and other cancers.

In a recent study, we investigated the therapeutic role of various derivatives containing the pyrano­[3,4-*b*]­indole ring in the treatment of TNBC.^24^ The results showed that potential EF2K inhibitor candidates were present at very low concentrations, such as 2.5 μM.^24^ Furthermore, the synthesis and anticancer activity of various derivatives containing a pyrano­[3,4-*b*]­indole ring have been reported.^46,47^ In a reported drug repositioning study, the effects of a combination of etodolac and alpha-tocopherol, known for their antioxidant properties, on amyloid-beta (A*β*)-related pathology in individual brain cell types and multiple pathways involved in AD pathology were investigated using *in vitro* and *in vivo* models, compared to the use of the drug alone.^21^ Therefore, the importance of combination therapy to simultaneously target multiple disease pathways has been highlighted, and the design and combination of etodolac and alpha tocopherol has been proposed as a novel therapeutic strategy against AD.^21^

### 2.4 *In Silico* Studies

#### 2.4.1 Molecular Docking, MD Simulations, and Binding Free Energy Calculations

In molecular docking studies against cholinesterases using the Glide/SP method, the highest binding energies to AChE were calculated for **ECC** (−10.503 kcal/mol), **ECA** (−9.899 kcal/mol), and **ECB** (−9.610 kcal/mol), respectively. The binding energy values of **ECF** (−8.394 kcal/mol), **ECH** (−8.150 kcal/mol), **ECK** (−8.141 kcal/mol), **ECL** (−8.137 kcal/mol), and **ECM** (−8.068 kcal/mol) compounds showed values close to the binding energy of tacrine. Furthermore, the highest binding energy values to BChE were calculated for **ECC** (−8.029 kcal/mol), **ECB** (−8.024 kcal/mol), **ECH** (−7.618 kcal/mol), **ECD** (−7.576 kcal/mol), **ECE** (−7.458 kcal/mol), **ECG** (−7.337 kcal/mol), **ECF** (−7.239 kcal/mol), **ECA** (−6.755 kcal/mol), **ECL** (−6.727 kcal/mol), **ECK** (−6.326 kcal/mol), **ECM** (−6.273 kcal/mol), respectively.

The average Root-Mean-Square Deviation (RMSD) protein Cα values (Å) and binding free energy (ΔG_bind_ kcal/mol) values obtained using the Molecular Mechanics/Generalized Born Surface Area (MM/GBSA) method during MD simulations, are shown in Table 3. For AChE-ligand complexes, the average RMSD values of the protein were calculated to be in the range of 1.41 ± 0.15 Å–1.86 ± 0.30 Å. The protein–ligand complexes were determined to be stable throughout the MD simulation. The RMSD plots of AChE-**ECA**, AChE-**ECB**, and AChE-**ECC** complexes showed that the average RMSD values of the protein were approximately 1.56 ± 0.16 Å, 1.72 ± 0.36 Å, and 1.53 ± 0.16 Å, respectively. This indicates that the complex remained stable throughout the MD simulation (Figure 3). Root-Mean-Square Fluctuation (RMSF) plots are presented to examine and characterize the effect of the mobility of each amino acid residue atom during the MD simulation (Figures 3 and 4). The RMSF values of the AChE complexes were generally below 2.5 Å. For one residue, the fluctuation exceeded 4 Å (Figure 3). The RMSF values of the BChE complexes were below 2 Å; however, in **ECA, ECB, ECD, ECE, ECG,** and **ECL** compounds, the fluctuation at residue 375 exceeded 2 Å (Figure 4).

**Table 3:** Average Protein Cα RMSD (Å) during MD Simulations and Average Binding Free Energy (ΔG_bind_ kcal/mol) Calculated via MM/GBSA^a^

Compounds	RMSD (Å) 4EY7	RMSD (Å) 6I0C	MM/GBSA 4EY7	MM/GBSA 6I0C
ECA	1.56 ± 0.16	1.43 ± 0.13	–74.89 ± 8.91	–54.86 ± 5.32
ECB	1.72 ± 0.36	1.41 ± 0.14	–58.51 ± 9.43	–48.39 ± 8.15
ECC	1.53 ± 0.16	1.39 ± 0.13	–68.19 ± 8.43	–72.34 ± 3.67
ECD	1.71 ± 0.24	1.39 ± 0.12	–46.78 ± 5.13	–44.81 ± 5.91
ECE	1.68 ± 0.27	1.48 ± 0.18	–52.24 ± 7.37	–47.61 ± 3.29
ECF	1.63 ± 0.25	1.32 ± 0.11	–51.93 ± 3.62	–51.63 ± 6.48
ECG	1.86 ± 0.30	1.45 ± 0.18	–46.20 ± 6.26	–50.79 ± 4.15
ECH	1.41 ± 0.15	1.37 ± 0.13	–57.10 ± 6.99	–54.76 ± 5.90
ECK	1.60 ± 0.15	1.59 ± 0.15	–53.32 ± 6.22	–52.29 ± 4.83
ECL	1.79 ± 0.23	1.48 ± 0.11	–48.06 ± 5.60	–44.56 ± 4.93
ECM	1.63 ± 0.30	1.37 ± 0.12	–61.34 ± 7.07	–51.67 ± 2.79
Tacrine^a,b^	1.31 ± 0.11^a^	1.38 ± 0.18^b^	–49.12 ± 3.15^a^	–56.67 ± 2.22^b^

a For tacrine, a and b refer to refs 28 and 36, respectively.

**Figure 3:**
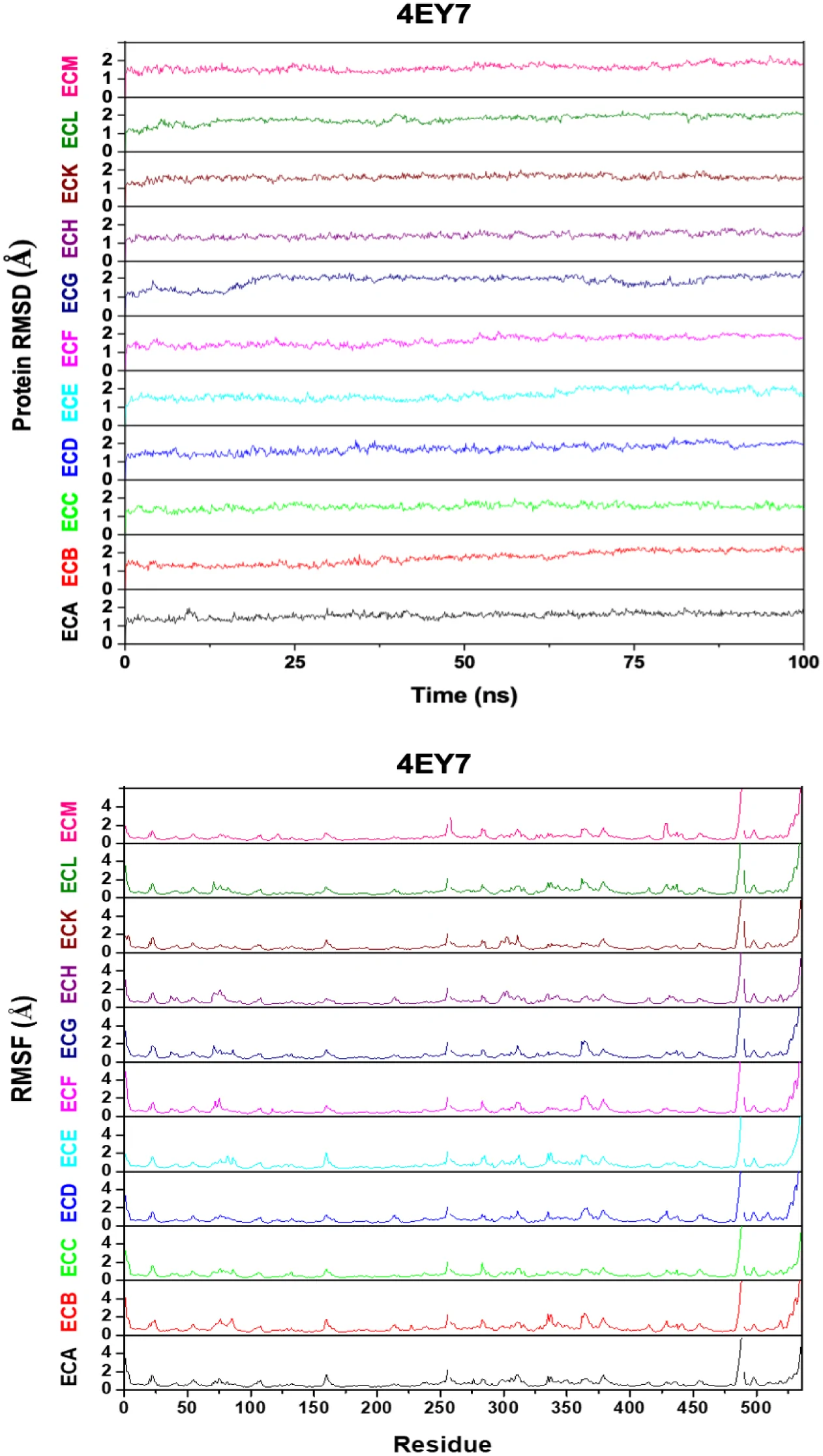
Time-dependent RMSD plots of the AChE protein Cα atoms in complex with the synthesized compounds during the MD simulation trajectory. RMSF profiles of the AChE amino acid residues, illustrating the local conformational flexibility and residual fluctuations upon ligand binding.

**Figure 4:**
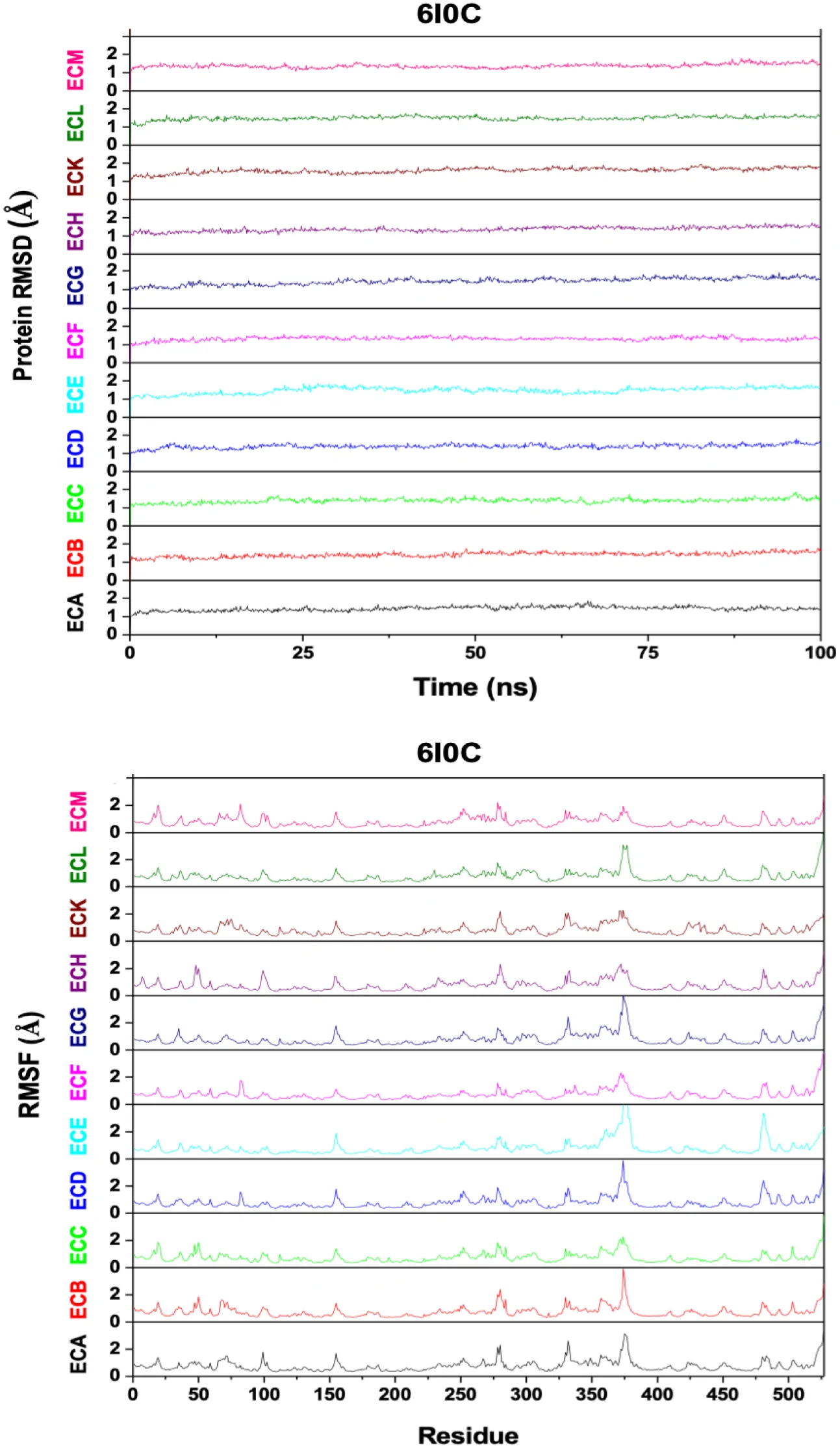
Time-dependent RMSD plots of the BChE protein Cα atoms in complex with the synthesized compounds during the MD simulation trajectory. RMSF profiles of the BChE amino acid residues, illustrating the local conformational flexibility and residual fluctuations upon ligand binding.

For the BChE-ligand complexes, the average RMSD values of the protein Cα were calculated to range from 1.32 ± 0.11 Å to 1.59 ± 0.15 Å. The RMSD graph of the BChE-**ECB** complex determined that the average RMSD value of the protein Cα was approximately 1.41 ± 0.14 Å. The average binding free energies of the protein–ligand complexes targeting AChE were calculated to range from −74.89 ± 8.91 kcal/mol to −46.20 ± 6.26 kcal/mol. Among the synthesized compounds, those with the highest binding free energy values were AChE**-ECA** (ΔG_bind_ = −74.89 ± 8.91 kcal/mol) and BChE**-ECC** (ΔG_bind_ = −72.34 ± 3.67 kcal/mol) (Table 3 and Figure 5). We have recently reported the average RMSD and average binding free energy values for the reference drugs donepezil (for AChE) and tacrine (for both AChE and BChE).^28,36^ Accordingly, the average binding free energy values of **ECA** and donepezil are very close to each other. However, the **ECC** compound has a much stronger binding free energy to the BChE compared to the reference inhibitor tacrine. In addition, the binding free energy and average RMSD values for tacrine were reported in a study using different crystal structures of proteins.^48^

**Figure 5:**
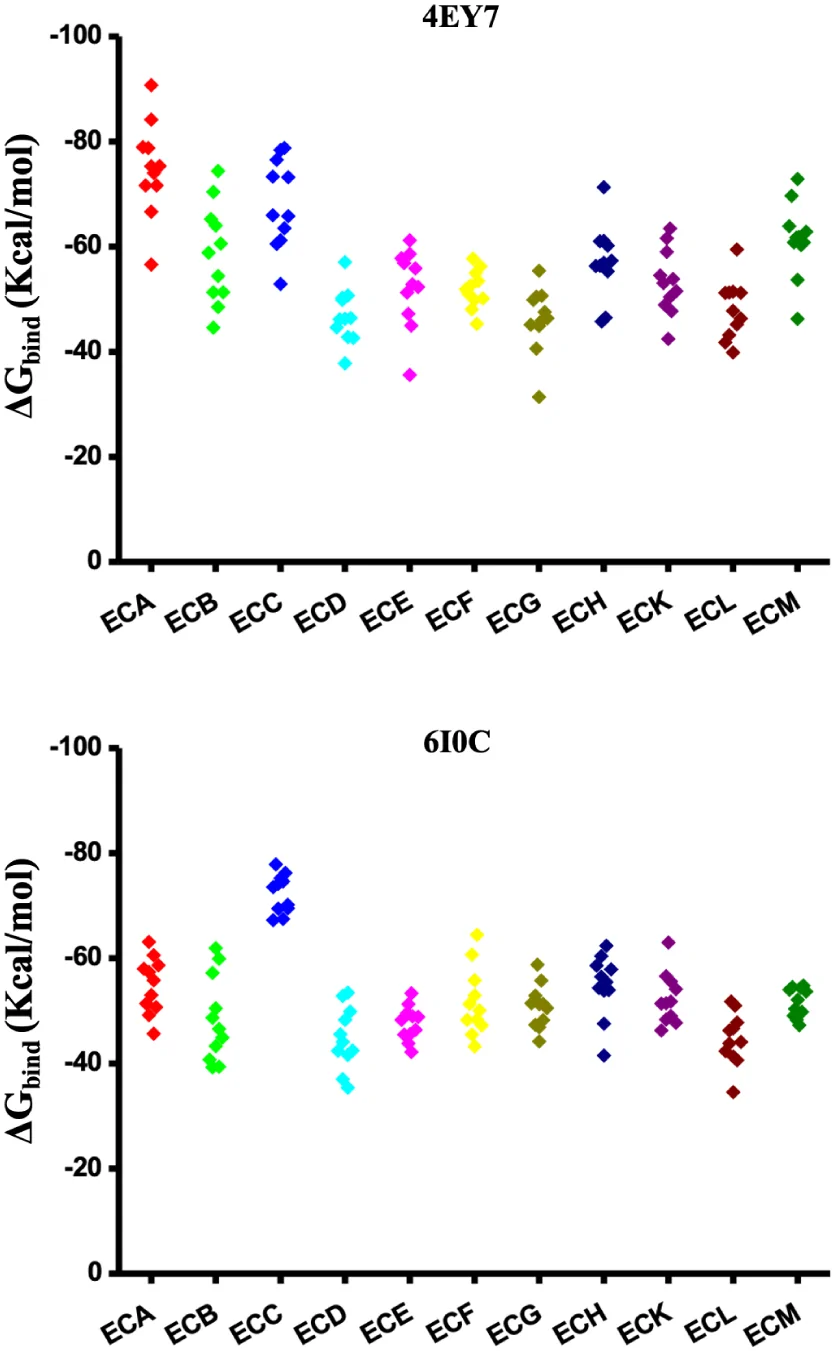
Average binding free energy profiles (ΔG_bind_ kcal/mol) of the target protein–ligand complexes estimated by the MM/GBSA.

Furthermore, it was determined that the interactions between the protein AChE and **ECA** occur via hydrogen bonds, hydrophobic interactions, and water bridges with crucial residues such as Tyr124, Tyr341, Phe295, Phe338, Tyr72, His287, Ser203, and Gly122 (Figure 6A). The interactions between BChE and **ECA** were identified with the residues Asn68, Asn289, Phe329, Trp231, and Pro285, including hydrogen bonds, hydrophobic interactions, and water bridges. These results demonstrate that interactions occur with expected residues in the enzyme’s binding pocket, and strong interactions such as H-bonding are formed (Figure 6B). The interactions between crucial residues of the proteins and **ECB** as an AChE/BChE dual inhibitor are shown in Figure 7A, B. The amide group of **ECB** interacts with AChE residues, including Tyr124 and Tyr341, while the morpholine oxygen contributes to interactions with Ser203 and Gly122 (Figure 7A). The oxygen atoms of the carbonyl and morpholine groups interact with AChE residues, including His438, Gly116, Ser198, and Tyr440 (Figure 7B). The **ECC** compound interacted with the critical residues of the proteins throughout MD simulations, and protein–ligand contacts are shown in Figure 8A, B. The nitrogen atom in the piperidine ring interacted with Trp86 and Trp82 residues to form pi-cation (Figure 8A, B). In addition, oxygen and nitrogen atoms formed hydrogen bonds with Tyr124, Asp74, and Phe295 residues via water bridges (Figure 8A). A pi–pi stacking occurred between the phenyl ring and the Trp231 residue (Figure 8B). Figure 9 shows the interactions of **ECG**, the second inhibitor with the highest IC_50_ value for BChE. In addition to its strong *in vitro* result (IC_50_ = 28.95 ± 0.65 nM), it was also supported *in silico* by interacting with crucial residues of the target (Figure 9).

**Figure 6:**
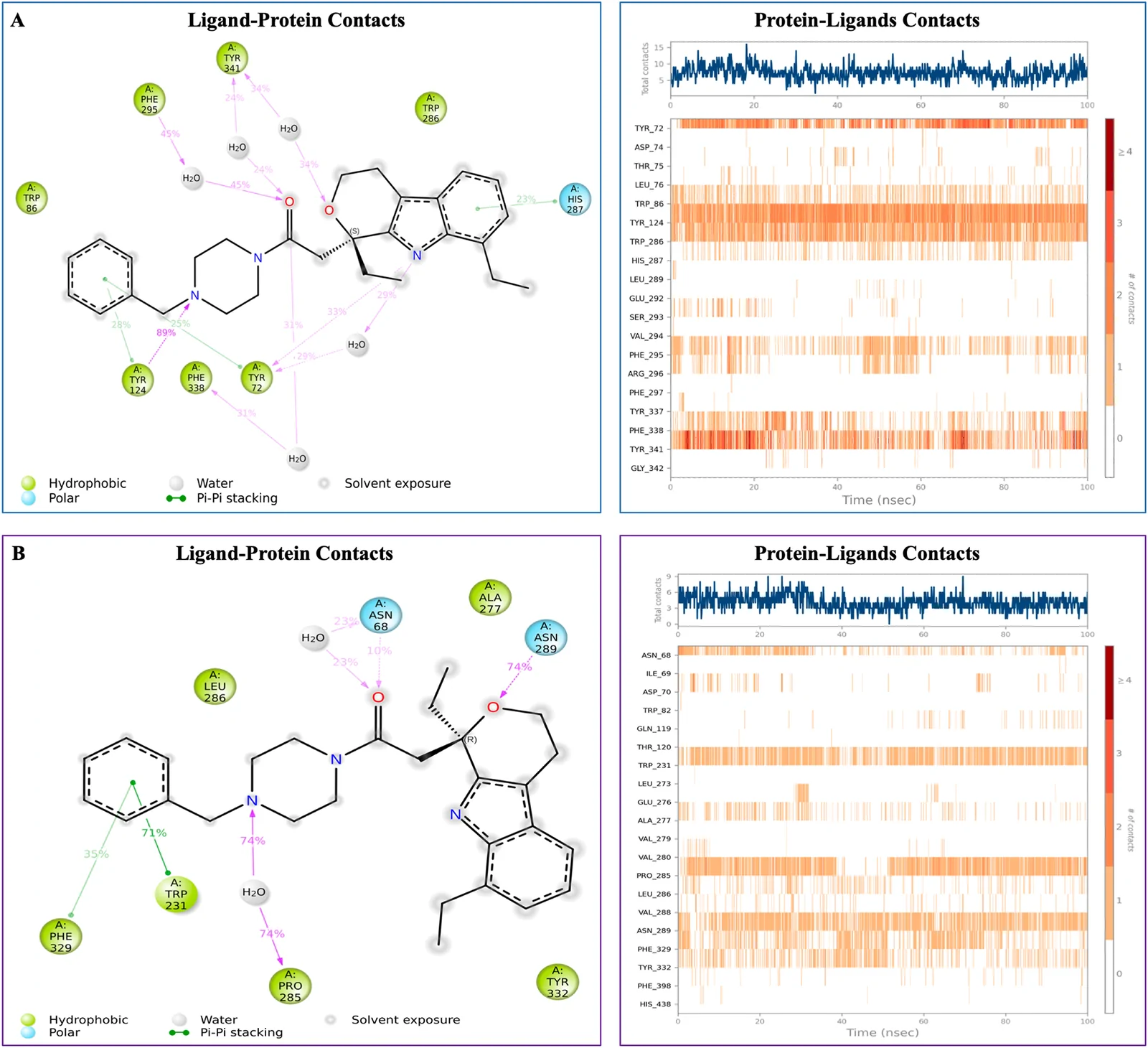
Ligand-protein and protein–ligand contacts through MD simulations. A) AChE-**ECA** and B) BChE-**ECA**.

**Figure 7:**
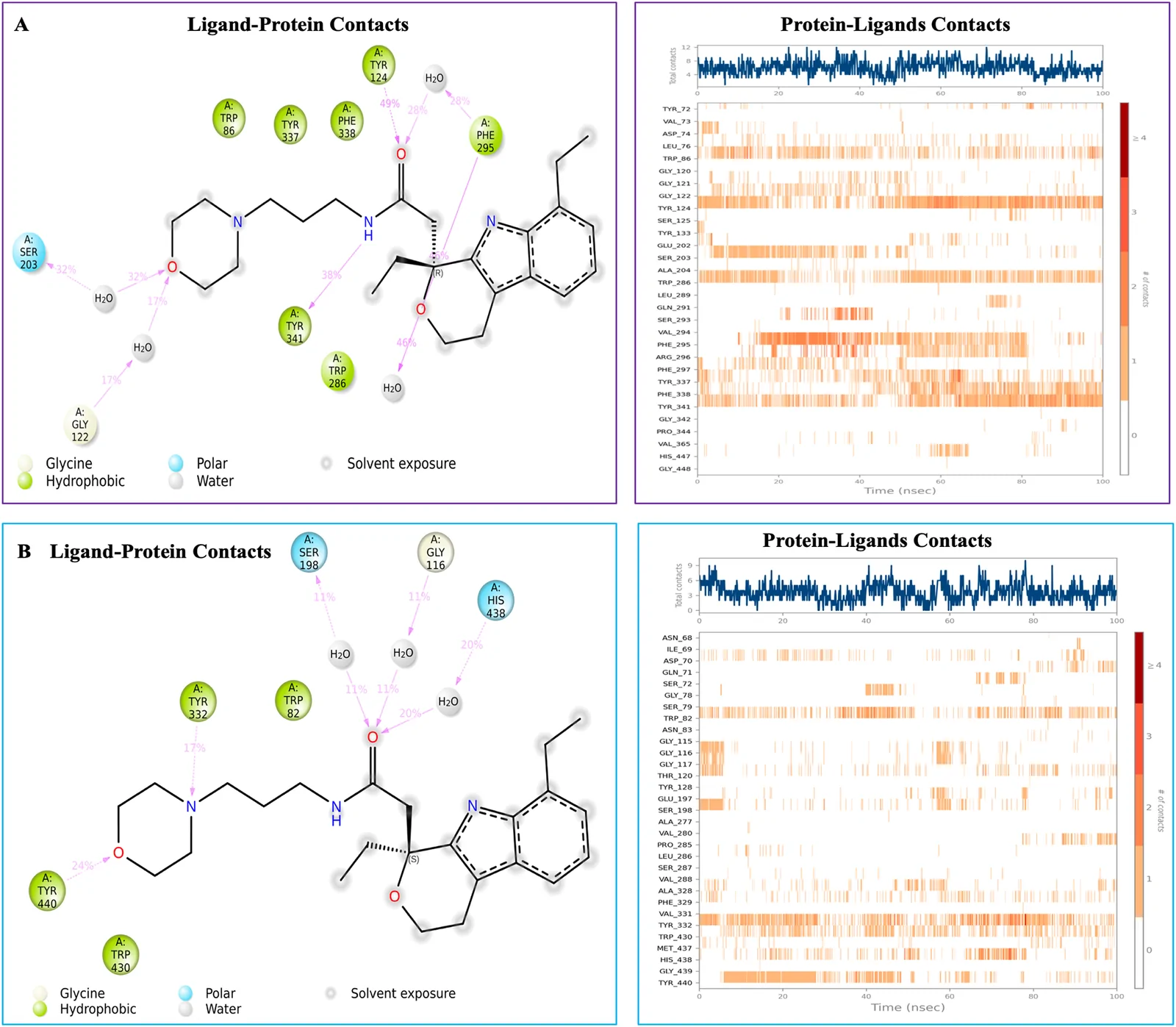
Ligand-protein and protein–ligand contacts of dual inhibitor **ECB** through MD simulations. A) AChE B) BChE.

**Figure 8:**
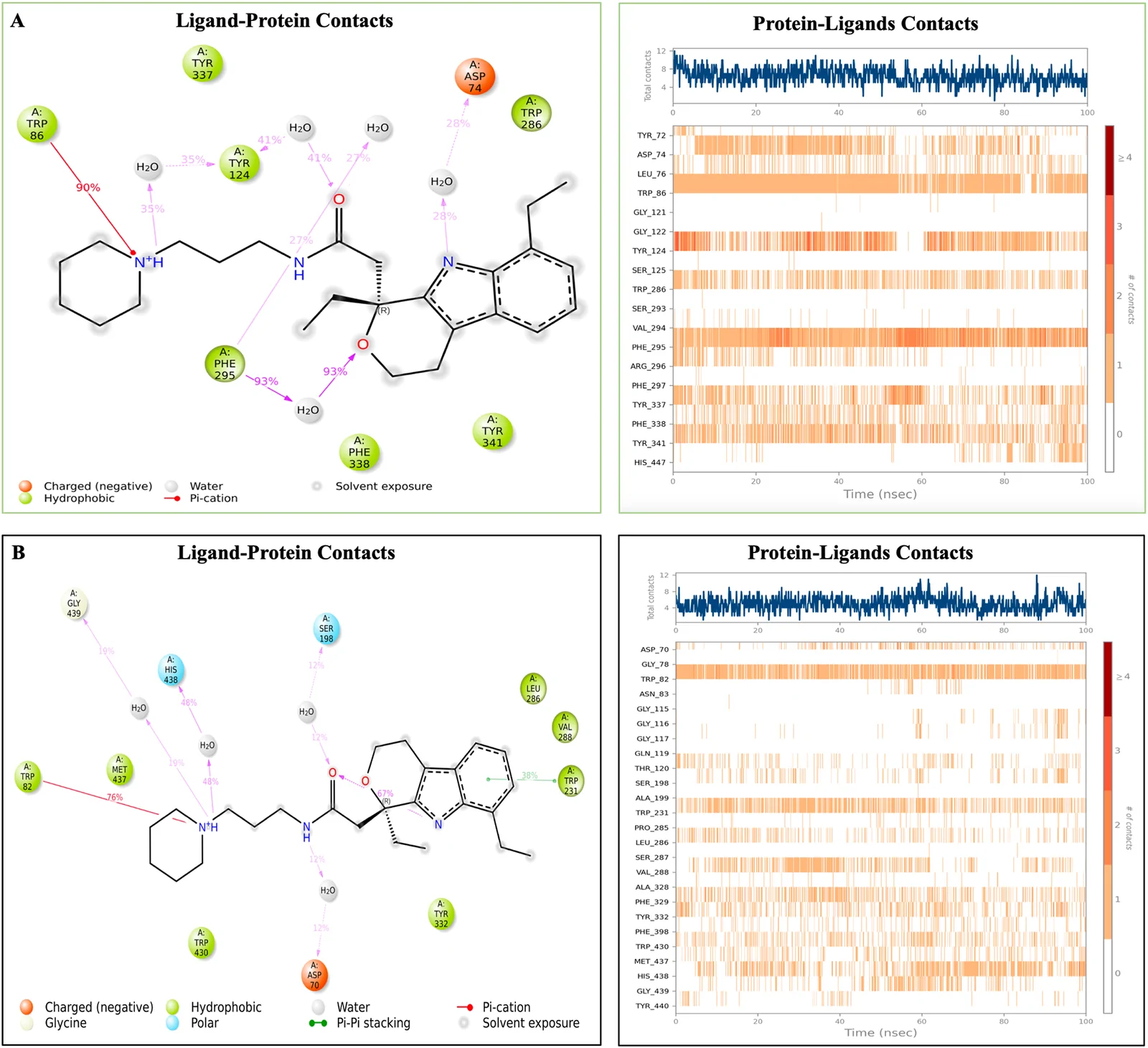
Ligand-protein and protein–ligand contacts through MD simulations. A) AChE-**ECC** and B) BChE-**ECC**.

**Figure 9:**
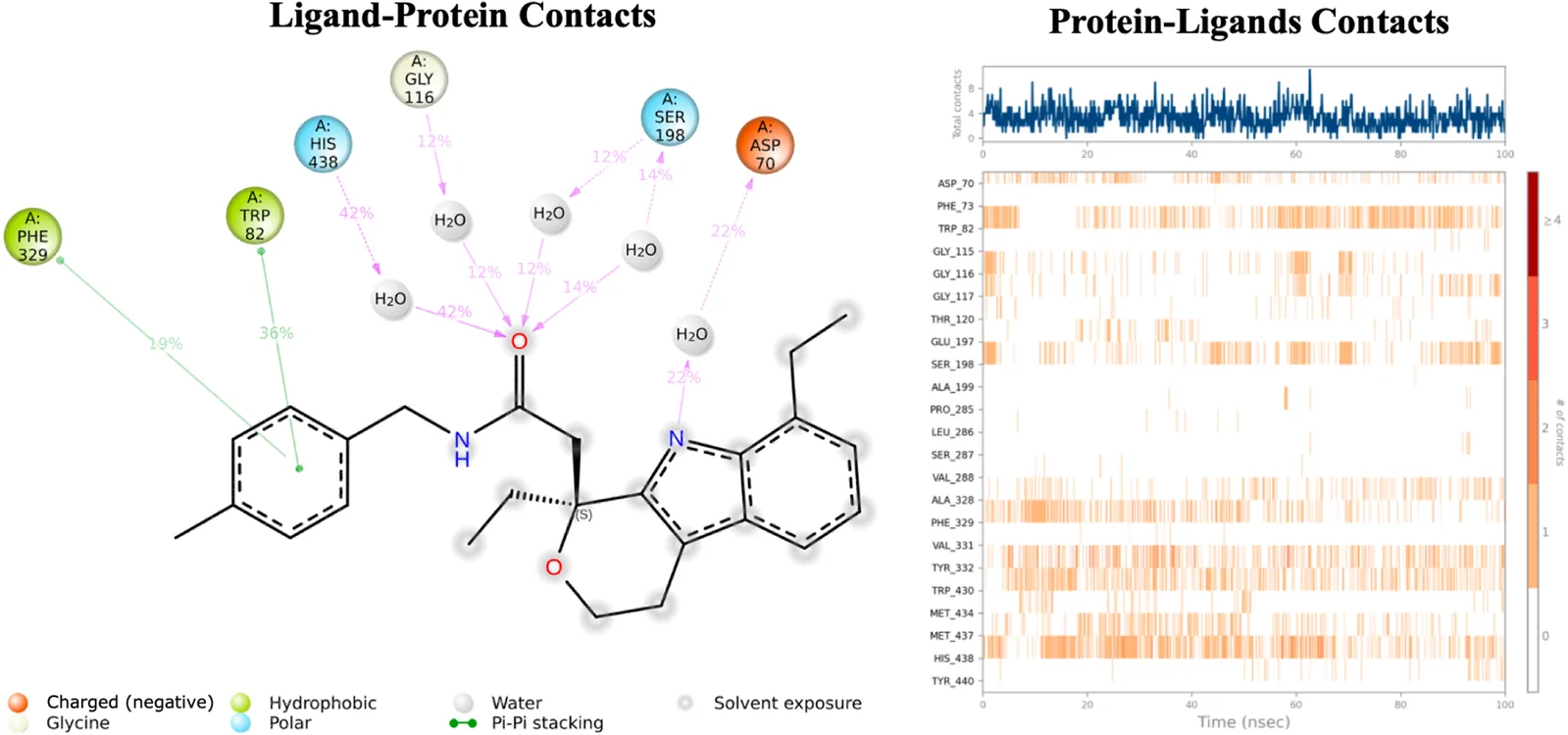
Ligand-protein and protein–ligand contacts of **ECG**. The second inhibitor with the highest IC_50_ value for BChE.

In recent years, many researchers have reported the discovery and development of new and effective AChE inhibitors for the treatment of AD.^28,36–38,48–52^ H-bonding, pi–pi stacking, and pi–pi cation interactions have been observed in the binding pocket of AChE with critical residues such as Phe295, Tyr341, Phe338, Arg296, Gly121, Tyr337, His447, Trp86, and Trp286 between the target enzyme and natural-synthetic hybrid compounds.^51^ The active site of AChE has been reported to contain subregions with various amino acid residues such as Phe295, Trp86, Trp286, and Tyr337.^48,52–54^ Previous studies have reported that MD simulations reveal the interactions for coumarin-based compounds.^52,55^ The AChE binding pocket contains the residues Tyr337, Trp86, Phe338, Tyr341, Asp74, and Gly120.^55^ In addition, AChE inhibitors bind to the catalytic active site, which includes the catalytic triad Ser200, Glu327, and His440.^53,56^ Trp84 and Phe330 residues have been reported to play a significant role in the catalytic reaction.

Furthermore, the secondary noncholinergic function of AChE is associated with the peripheral anionic site containing the residues Tyr70, Asp72, Tyr121, Trp279, and Tyr334. As previously reported, both peripheral anionic site (PAS) and catalytic active site (CAS) are potential targets for the discovery of novel compounds in AD.^53,57^ In addition, binding poses of some ligands in the CAS and PAS regions of AChE were obtained, and it was found that one of the ligands interacts with the key residues.^58,59^ The integration of experimental IC_50_ values with computational modeling provides an insight into the inhibition mechanism. Binding poses and subsequent MD simulations reveal that the compounds are stable in the active site and interact with key residues in the CAS and PAS. Supported by favorable MM/GBSA binding free energies, these findings suggest that these compounds may be suitable inhibitors for the enzyme’s binding sites.

Notably, while compound **ECC** displayed more favorable computational profiles in both molecular docking (−10.503 kcal/mol) and MM/GBSA calculations (AChE: ΔG_bind_ = −68.19 ± 8.43 kcal/mol; BChE: ΔG_bind_ = −72.34 ± 3.67 kcal/mol), compound **ECB** demonstrated slightly superior experimental potency in the *in vitro* assays against both target enzymes (AChE: IC_50_ = 35.31 ± 0.81 nM; BChE = 28.91 ± 0.19 nM). This minor variation in the relative potency ranking of the two lead candidates is a recognized phenomenon in computer-aided drug design. Experimental variables such as solubility, kinetic stability in the assay buffer, or minor differences in binding kinetics can exert a profound influence on the observed activity. Nevertheless, both synthesized compounds significantly outperformed the reference drug, tacrine (AChE: IC_50_ = 75.63 ± 0.77 nM; BChE 45.47 ± 1.57 nM), validating the predictive value of our computational workflow for identifying high affinity nanomolar inhibitors.

#### 2.4.2 ADMET Descriptors and Toxicity Prediction

A comprehensive ADMET (Absorption, Distribution, Metabolism, Excretion, Toxicity) and Lipinski’s Rule of Five (Ro5) analyses was performed to evaluate the pharmacokinetic suitability and drug similarity of the synthesized pyrano­[3,4-*b*]­indole derivatives. The results are summarized in Table 4. The molecular weights (MWs) of all compounds (**ECA-ECM**) ranged from 380.45 to 445.59 g/mol, well below the 500 g/mol threshold defined by Lipinski. Furthermore, the number of hydrogen bond donors (HBD ≤ 5) and hydrogen bond acceptors (HBA ≤ 10) were within the ideal ranges, indicating high membrane permeability and oral bioavailability. However, the lipophilicity profiles (AlogP98) show that **ECA, ECD, ECE, ECG**, **ECK, ECL,** and **ECM**, exhibit values above or near the optimal limit of 5.0. In contrast, **ECB** (AlogP98 = 3.19), **ECC** (AlogP98 = 4.419), and **ECF** (AlogP98 = 4.868) demonstrate a more balanced lipophilic-hydrophilic character, reflected in their improved solubility levels. The **ECH** compound stands out as a less favorable candidate for central nervous system (CNS) treatment, likely due to its higher polar surface area or structural properties, as it exhibits low blood–brain barrier (BBB) penetration and moderate absorption. Metabolically, most compounds were predicted to be CYP2D6 inhibitors. Notably, **ECA, ECH**, and **ECM** were predicted to be noninhibitory, suggesting a different metabolic clearance pathway. Based on the ADMET prediction, **ECB** and **ECC** emerge as the most promising candidates. These compounds, which provide an optimal balance between lipophilicity (AlogP), BBB permeability, and water solubility, are suitable candidates for further *in vivo* research and lead optimization. The calculated AlogP98 value for the **ECA** compound was found to be 5.048. Although this value is slightly above the ideal threshold of 5.0 typically associated with optimal cell permeability, it indicates a high lipophilic character that may facilitate passive diffusion across biological membranes. This small deviation does not significantly violate the drug-likeness criteria and demonstrates a favorable pharmacokinetic profile. All compounds (**ECA-ECM**) were predicted to be nonmutagenic. This is important data indicating that the compounds have a low potential for DNA damage and a low genotoxic risk (Supporting Table S1). Moreover, most of the compounds exhibit a noncarcinogenic profile in mouse and rat models. This provides theoretical evidence that the compounds may be safe for long-term use (Supporting Table S1).

**Table 4:** *In silico* ADMET Estimation via BIOVIA Discovery Studio^a^

Compounds	ADMET solubility level	ADMET BBB level	ADMET CYP2D6	ADMET absorption level	ADMET AlogP98	Number H Acceptors Lipinski	Number H Donor Lipinski	Molecular Weight (g/mol)
ECA	1	1	false	0	5.048	5	1	445.596
ECB	2	2	true	0	3.19	6	2	413.553
ECC	2	1	true	0	4.419	5	2	411.58
ECD	1	1	true	0	5.09	4	2	394.482
ECE	1	0	true	0	5.549	4	2	410.936
ECF	1	1	true	0	4.868	5	2	406.517
ECG	1	1	true	0	5.371	4	2	390.518
ECH	1	4	false	1	4.779	7	2	421.489
ECK	1	1	true	0	5.083	4	2	380.455
ECL	1	0	true	0	5.542	4	2	396.91
ECM	1	0	false	0	5.626	4	2	441.361

a Solubility is rated on a scale where 0 denotes very low solubility, 1 indicates very low but feasible solubility, 2 represents low solubility, and 3 signifies good solubility. Blood–Brain Barrier (BBB) permeability is classified as 0 for very high permeability, 1 for high, 2 for moderate, 3 for low, and 4 for undefined. The Cytochrome P450 2D6 status is indicated as “True” for inhibitors and “False” for noninhibitors. AlogP98 value below 5 is considered indicative of ideal cell permeability.

### 2.5 Structure–Activity Relationship

Figure 10 illustrates a pharmacophore model for the **ECB** compound. The core structure of **ECB** (orange and light blue spheres) supports high-affinity ligand binding by forming stable pi–pi and alkyl interactions with the enzyme residues. The NH proton of the amide group (pink sphere) functions as a hydrogen bond donor. For this study, a modification in this group (e.g., *N*-methylation) can disrupt the ideal orientation of the molecule. The tertiary amine function in the morpholine ring (red sphere) acquires an ionic character through protonation under physiological conditions. This positive center forms strong cation-pi interactions with the aromatic residues of both enzymes. The piperidine and piperazine rings of the **ECA** and **ECC** compounds exhibit similar properties. Additional heteroatoms, such as the oxygen atom in the morpholine ring, can determine the selectivity profile by forming extra polar interactions with surrounding water molecules or amino acid side chains. The aliphatic chain length (−CH_2_−) acts as an ideal linker to bridge the distance between the PAS and CAS regions. The flexibility of this chain may play a role in the molecule’s compatibility with both enzymes. A change of 1–2 carbons in chain length can play a decisive role in the activity. In the pharmacophore model, hydrophobic moieties at three different points (light blue spheres) allow the molecule to bind to hydrophobic vacancies within the protein. The presence of groups (e.g., methyl or ethyl) at these sites may increase affinity for tight binding. However, the addition of polar groups (OH, NH_2_) to these sites would likely decrease activity as it would disrupt the hydrophobic interaction. Consequently, the obtained biological data demonstrate an excellent selectivity of the molecule. The high correlation between *in silico* and *in vitro* results proves that the groups present in the molecule exhibit potent cholinesterase inhibition.

**Figure 10:**
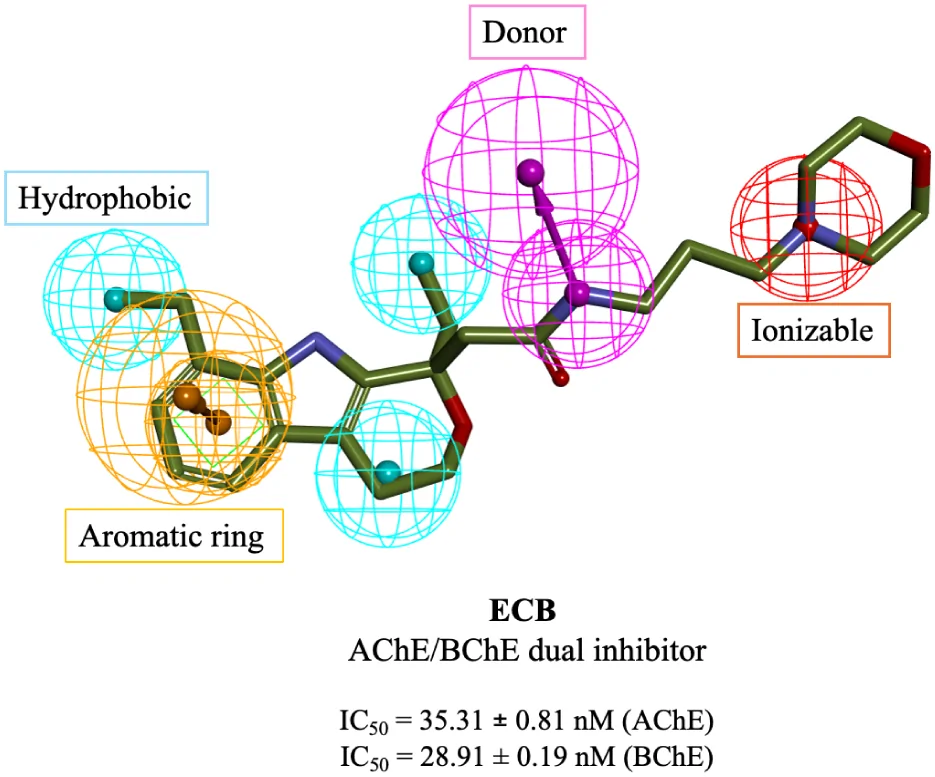
A pharmacophore model of the AChE/BChE dual inhibitor **ECB**.

## 3 Conclusions

In this study, 11 novel pyrano­[3,4-*b*]­indole derivatives were designed, synthesized, characterized, and evaluated *in vitro* for their biological activities, including enzyme inhibitory and anticancer effects. All compounds (**ECA-ECM**) showed strong enzyme-inhibition against AChE and BChE. Among all synthesized compounds, **ECB** was determined to be the most effective molecule with the lowest IC_50_ values, and to be a dual enzyme inhibitor (AChE/BChE). While compound **ECH** exhibited a comparable IC_50_ value against AChE (80.02 ± 0.60 nM) to tacrine (75.63 ± 0.77 nM), it was less effective than the other tested compounds. However, **ECH** showed a strong enzyme inhibitory effect against BChE compared to the reference drug tacrine. Although the **ECH** has good binding free energy, the presence of a nitro group in its structure may have had a negative effect on AChE inhibition. The anticancer activity results of the **ECH** compound in five different cell lines were not as expected (above 100 μM). Therefore, **ECH** containing a nitro functional group was not found to be ideal as an anticancer agent and AChE inhibitor at low doses; rather, it may be a BChE inhibitor. Regarding the anticancer activities of the compounds, **ECK** containing 4-fluoroaniline showed the highest cytotoxic effect at low doses in five different cell lines, particularly in colon and TNBC cell lines. According to our findings, **ECD, ECF, ECG, ECL**, and **ECM** showed strong anticancer activity in most cell lines. These compounds are structurally aromatic amide-substituted pyrano­[3,4-*b*]­indole derivatives.

Despite the application of various synthetic strategies and optimization efforts, compound **ECC** was obtained with a low yield. The **ECC** compound has shown promising *in vitro* activity. Future studies will focus on optimizing the synthetic route to overcome the current low yields and facilitate more comprehensive *in vivo* studies. The results regarding AD have revealed that the synthesized compounds are promising multitarget candidates for AChE and BChE and dual AChE/BChE inhibitors. In addition, most of the compounds exhibited significant cytotoxic effects in the treatment of breast and colon cancers. This study may offer a new perspective in the design and development of potential inhibitor candidates for AD and cancer.

## 4 Experimental Section

### 4.1 General

All chemicals and solvents were obtained from Sigma, Merck, Apollo Scientific, and VWR. FT-IR spectra were recorded on a PerkinElmer Spectrum 100 FT-IR spectrometer. ^1^H and ^13^C NMR spectra were acquired using JEOL ECX-400 and Agilent 600 MHz NMR spectrometers. MS analyses were performed using a Shimadzu LC-MS/MS 8040 system and QTOF-HRMS Agilent 6530 Accurate-Mass. Chemical shifts (δ) are reported in parts per million (ppm), with coupling constants (*J*) given in Hertz (Hz) to indicate signal multiplicity. The NMR signals are reported with the following abbreviations: (s) singlet, (d) doublet, (t) triplet, (m) multiplet, (dd) doublet of doublets, and (qd) quartet of doublets. MS confirmed the molecular ion as the predominant species with no significant impurity ions detected.

For *in vitro* enzyme-activity studies, AChE from *Electrophorus electricus* (electric eel) (Sigma C2888-500UN) and BChE from equine serum (C7512-1.2KU) were purchased. For cytotoxic activity, DMEM/F-12 (1:1) (CE) (500 mL) (Thermo Fisher Scientific (Gibco), trypsin (Thermo Fisher Scientific (Gibco)), Penicillin/Streptomycin (PS) (Thermo Fisher Scientific (Gibco)), and Fetal Bovine Serum (FBS) (Serox) were purchased. MTT reagent was obtained from Cayman chemical.

### 4.2 Synthesis

1,8-Diethyl-1,3,4,9-tetrahydropyrano­[3,4-*b*]­indol-1-yl)­acetic acid (1 equiv), 1-ethyl-3-(3-(dimethylamino)­propyl)­carbodiimide hydrochloride (EDCI·HCl) (1.5 equiv), *N,N*-diisopropylethylamine (6 equiv), and 1-hydroxybenzotriazole hydrate (HOBt·H_2_O) (1.5 equiv) were stirred in dimethylformamide (DMF) for 1 h at room temperature (r.t.). The amine derivative (1.5 equiv) was then added, and the reaction was stirred at r.t. for 48 h. The reaction was monitored using thin layer chromatography (TLC). After the reaction was complete, the reaction mixture was added to cold water and stirred for approximately 10 min. Then, the mixture was extracted using ethyl acetate (EtOAc). The organic phase was washed with water and dried with Na_2_SO_4_ and removed using a rotary evaporator to obtain the crude product.^24^ The compound (**ECC**) was purified by the prep TLC. The structures of the synthesized compounds were elucidated by FT-IR, ^1^H NMR, ^13^C NMR, and MS spectroscopic analyses (Supporting Figure S1–S42).

***1-(4-Benzylpiperazin-1-yl)-2-(1,8-diethyl-1,3,4,9-tetrahydropyrano­[3,4-*** *b* ***]­indol-1-yl)­ethan-1-one (ECA)*:** FT-IR Spectrum (cm^–1^): 3379 (N–H), 3067, 3033 (Ar C–H), 2982–2851 (Aliphatic C–H), 1706 (C=O) (Supporting Figure S1). ^1^H NMR (400 MHz, DMSO-*d*_6_) δ 8.84 (s, 1H), 7.88–7.76 (m, 1H), 7.68 (t, *J* = 8.4 Hz, 2H), 7.61–7.50 (m, 2H), 7.45 (d, *J* = 7.9 Hz, 2H), 7.14 (d, *J* = 3.9 Hz, 1H), 4.37 (s, 2H), 4.10 (s, 2H), 1.49 (s, 3H). 3.35 and 2.52 (methylene protons of the piperazine overlapped with the solvent) (Supporting Figure S2). ^13^C NMR (100 MHz, CDCl_3_) δ 173.29, 135.93, 134.48, 126.63, 126.18, 120.41, 119.60, 115.96, 108.44, 74.62, 60.65, 51.97, 42.82, 30.68, 24.22, 22.42, 13.76, 7.59 (Supporting Figure S3). Formula: C_28_H_35_N_3_O_2_.

***2-(1,8-Diethyl-1,3,4,9-tetrahydropyrano­[3,4-*** *b* ***]­indol-1-yl)-N-(3-morpholinopropyl)­acetamide (ECB)*:** FT-IR Spectrum (cm^–1^): 3312 (N–H), 2928, 2870 (Aliphatic C–H), 1645 (C=O) (Supporting Figure S4). ^1^H NMR (600 MHz, DMSO-*d*_6_): δ 10.47 (s, 1H), 7.59 (t, *J* = 5.7 Hz, 1H), 7.19 (d, *J* = 7.6 Hz, 1H), 6.90–6.82 (m, 2H), 3.92 (dd, *J* = 6.4, 4.3 Hz, 2H), 3.51 (t, *J* = 4.6 Hz, 4H), 3.12–2.96 (m, 2H), 2.85–2.57 (m, 6H), 2.29 – 2.12 (m, 6H), 2.01–1.91 (m, 2H), 1.47 (*J* = 7.2 Hz, 2H), 1.22 (t, *J* = 7.5 Hz, 3H), 0.63 (t, *J* = 7.3 Hz, 3H) (Supporting Figure S5). ^13^C NMR (150 MHz, DMSO-*d*_6_): δ 169.76, 137.21, 134.82, 126.90, 126.53, 120.02, 119.13, 115.84, 107.29, 66.67, 66.64, 60.28, 56.18, 53.75, 53.72, 44.49, 37.25, 30.91, 26.41, 26.38, 24.20, 24.17, 22.41, 14.81, 8.24 (Supporting Figure S6). LC-MS/MS [M – 1]^−^ 412.30 g/mol (%100) (Supporting Figure S7). Formula: C_24_H_35_N_3_O_3_.

***2-(1,8-Diethyl-1,3,4,9-tetrahydropyrano­[3,4-*** *b* ***]­indol-1-yl)-N-(3-(piperidin-1-yl)­propyl) acetamide (ECC)*:** ^1^H NMR Spectrum (400 MHz, CDCl_3_) δ 9.86 (s, 1H), 8.12 (s, 1H), 7.33 (d, *J* = 7.8 Hz, 1H), 7.09–6.94 (m, 2H), 4.03 (dd, *J* = 17.0, 11.5 Hz, 2H), 3.35 (d, *J* = 5.8 Hz, 2H), 2.97–2.64 (m, 8H), 2.44–2.25 (m, 6H), 2.17 (t, *J* = 7.2 Hz, 2H), 2.01 (dd, *J* = 14.5, 7.2 Hz, 2H), 1.46 (s, 2H), 1.36 (t, *J* = 7.6 Hz, 4H), 1.26 (s, 2H), 0.87 (t, *J* = 7.4 Hz, 3H) (Supporting Figure S8). ^13^C NMR Spectrum: (100 MHz, CDCl_3_) δ 170.94, 134.58, 120.02, 119.36, 115.74, 107.35, 75.39, 60.56, 58.16, 54.35, 45.13, 25.88, 24.21, 22.47. ^13^C NMR (APT) Spectrum: (100 MHz, CDCl_3_) δ 170.95, 126.92, 120.00, 119.49, 115.74, 107.35, 104.60, 78.71, 75.38, 60.56, 54.33, 45.10, 30.47, 25.92, 24.20, 22.47, 7.72 (Supporting Figure S9). QTOF-HRMS [M + H]^+^ 412.29647; [M + H + 1]^+^ 413.30059; [M + H + 2]^+^ 414.30477 (Supporting Figure S10). Formula: C_25_H_37_N_3_O_2_.

***2-(1,8-Diethyl-1,3,4,9-tetrahydropyrano­[3,4-*** *b* ***]­indol-1-yl)-N-(4-fluorobenzyl)­acetamide (ECD)*:** FT-IR Spectrum (cm^–1^): 3286 (N–H), 3091 and 3050 (Ar C–H), 2966–2849 (Aliphatic C–H), 1621 (C=O) (Supporting Figure S11). ^1^H NMR (600 MHz, DMSO-*d*_6_): δ 10.47 (s, 1H), 8.09 (t, *J* = 6.1 Hz, 1H), 7.20 (dd, *J* = 7.7, 1.3 Hz, 1H), 7.15–7.08 (m, 2H), 7.00–6.93 (m, 2H), 6.91–6.82 (m, 2H), 4.32–4.15 (m, 2H), 3.98–3.89 (m, 2H), 2.89–2.68 (m, 4H), 2.67–2.53 (m, 2H), 1.99 (dd, *J* = 7.2, 1.6 Hz, 2H), 1.21 (t, *J* = 7.5 Hz, 3H), 0.63 (t, *J* = 7.3 Hz, 3H) (Supporting Figure S12). ^13^C NMR (150 MHz, DMSO-*d*_6_): δ 169.81, 162.23, 160.63, 134.87, 129.25, 129.20, 126.96, 120.03, 119.14, 115.85, 115.28, 115.14, 75.60, 41.72, 31.05, 24.16, 22.53, 22.34, 14.82, 8.26 (Supporting Figure S13). LC-MS/MS [M + 1]^+^ 395.20 g/mol (%100) (Supporting Figure S14). Formula: C_24_H_27_FN_2_O_2_.

***N-(4-Chlorobenzyl)-2-(1,8-diethyl-1,3,4,9-tetrahydropyrano­[3,4-*** ***b*** ***]­indol-1-yl)­acetamide (ECE)*:** FT-IR Spectrum (cm^–1^): 3396 (N–H), 3033 (Ar C=CH), 2942–2911 (Aliphatic C–H), 1642 (C=O) (Supporting Figure S15). ^1^H NMR (600 MHz, DMSO-*d*_6_): δ 10.46 (s, 1H), 8.10 (t, *J* = 6.1 Hz, 1H), 7.25–7.15 (m, 3H), 7.11–7.06 (m, 2H), 6.92–6.83 (m, 2H), 2.89–2.68 (m, 4H), 2.67–2.54 (m, 1H), 4.36–4.14 (m, 2H), 3.96–3.88 (m, 2H), 1.99 (qd, *J* = 7.2, 3.1 Hz, 2H), 1.20 (t, *J* = 7.5 Hz, 3H), 0.64 (t, *J* = 7.3 Hz, 3H) (Supporting Figure S16). ^13^C NMR (150 MHz, DMSO-*d*_6_): δ 169.86, 139.00, 137.03, 134.87, 129.13, 128.44, 126.97, 120.04, 119.14, 115.85, 107.38, 75.95, 60.42, 41.78 (Supporting Figure S17). LC-MS/MS [M + 1]^+^ 411.25 g/mol (%100) (Supporting Figure S18). Formula: C_24_H_27_ClN_2_O_2_.

***2-(1,8-Diethyl-1,3,4,9-tetrahydropyrano­[3,4-*** ***b*** ***]­indol-1-yl)-N-(4-methoxybenzyl)­acetamide (ECF)*:** FT-IR Spectrum (cm^–1^): 3278 (N–H), 3088–3049 (Ar C=CH), 2969–2865 (Aliphatic C–H), 1622 (C= O) (Supporting Figure S19). ^1^H NMR (600 MHz, CDCl_3_) δ 9.54–9.46 (m, 1H), 7.32 (d, *J* = 7.8 Hz, 1H), 7.05 (t, *J* = 7.5 Hz, 1H), 6.99 (d, *J* = 7.0 Hz, 1H), 6.94 (d, *J* = 8.5 Hz, 2H), 6.67 (d, *J* = 8.7 Hz, 2H), 4.50–4.12 (m, 2H), 4.05–3.94 (m, 2H), 3.75 (s, 3H), 3.01–2.62 (m, 8H), 2.18–1.92 (m, 3H), 1.32–1.26 (m, 3H), 0.86 (t, *J* = 7.4 Hz, 3H) (Supporting Figure S20). ^13^C NMR (150 MHz, CDCl_3_) δ 170.87, 158.78, 135.87, 134.76, 129.92, 128.73, 127.00, 126.25, 120.24, 119.48, 115.77, 113.91, 107.65, 75.78, 60.48, 55.25, 44.29, 42.80, 30.90, 29.70, 24.06, 22.24, 13.88, 7.77 (Supporting Figure S21). QTOF-HRMS [M + H]^+^ 407.23167; [M + Na]^+^ 429.21358 (Supporting Figure S22). Formula: C_25_H_30_N_2_O_3_.

***2-(1,8-Diethyl-1,3,4,9-tetrahydropyrano­[3,4-*** ***b*** ***]­indol-1-yl)-N-(4-methylbenzyl)­acetamide (ECG)*:** FT-IR Spectrum (cm^–1^): 3280 (N–H), 3049 (Ar C–H), 2969–2838 (Aliphatic C–H), 1623 (C=O) (Supporting Figure S23). ^1^H NMR (600 MHz, DMSO-*d*_6_): δ 10.47 (s, 1H), 8.05 (t, *J* = 6.0 Hz, 1H), 7.19 (dd, *J* = 7.7, 1.2 Hz, 1H), 7.05–6.98 (m, 4H), 6.92–6.82 (m, 2H), 4.27–4.10 (m, 2H), 3.98–3.85 (m, 2H), 2.88–2.67 (m, 4H), 2.65–2.54 (m, 2H), 2.22 (s, 3H), 2.00 (d, *J* = 7.3 Hz, 2H), 1.21 (t, *J* = 7.5 Hz, 3H), 0.62 (t, *J* = 7.3 Hz, 3H) (Supporting Figure S24). ^13^C NMR (150 MHz, DMSO-*d*_6_): δ 169.75, 137.16, 136.78, 136.01, 129.14, 127.39, 126.92, 126.55, 120, 119.12, 115.84, 107.35, 75.95, 60.30, 42.22, 30.97, 24.15, 22.37, 21.08, 14.80, 8.24 (Supporting Figure S25). LC-MS/MS [M – 1]^−^ 391.30 g/mol (%100) (Supporting Figure S26). Formula: C_25_H_30_N_2_O_2_.

***2-(1,8-Diethyl-1,3,4,9-tetrahydropyrano­[3,4-*** ***b*** ***]­indol-1-yl)-N-(4-nitrobenzyl)­acetamide (ECH)*:** FT-IR Spectrum (cm^–1^): 3336, 3276 (N–H), 3109, 3078 (Ar C=C), 2963–2870 (Aliphatic C–H), 1627 (C=O) (Supporting Figure S27). ^1^H NMR (600 MHz, DMSO-*d*_6_): δ 10.46 (s, 1H), 8.19 (t, *J* = 6.1 Hz, 1H), 7.95–7.89 (m, 2H), 7.28–7.22 (m, 2H), 7.19 (dd, *J* = 7.7, 1.3 Hz, 1H), 6.91–6.83 (m, 2H), 4.40–4.29 (m, 2H), 4.03–3.92 (m, 2H), 2.92–2.72 (m, 4H), 2.66–2.57 (m, 2H), 1.99 (m, 2H), *J* = 18.7, 7.2 Hz, 2H), 1.22–1.16 (m, 3H), 0.66 (t, *J* = 7.3 Hz, 3H) (Supporting Figure S28). ^13^C NMR (150 MHz, DMSO-*d*_6_): δ 170.05, 158.84, 148.19, 146.63, 136.90, 134.86, 128.18, 127.00, 123.64, 120.08, 119.16, 115.85, 107.38, 75.96, 60.34, 44.27, 42.06, 31.15, 24.16, 22.31, 14.84, 8.24 (Supporting Figure S29). LC-MS/MS [M – 1]^−^ 420.25 g/mol (%100) (Supporting Figure S30). Formula: C_24_H_27_N_3_O_4_.

***2-(1,8-Diethyl-1,3,4,9-tetrahydropyrano­[3,4-*** ***b*** ***]­indol-1-yl)-N-(4-fluorophenyl)­acetamide (ECK)*:** FT-IR Spectrum (cm^–1^): 3306 (N–H), 3061 (Ar C=CH), 2995–2877 (Aliphatic C–H), 1671 (C=O) (Supporting Figure S31). ^1^H NMR (600 MHz, DMSO-*d*_6_): δ 10.45 (s, 1H), 9.70 (s, 1H), 7.57–7.52 (m, 2H), 7.20 (d, J = 7.5 Hz, 1H), 7.11–7.05 (m, 2H), 6.92–6.82 (m, 2H), 4.03–3.92 (m, 2H), 3.02–2.92 (m, 2H), 2.87–2.78 (m, 2H), 2.70–2.56 (m, 2H), 2.10–2.01 (m, 1H), 1.25–1.19 (m, 2H), 0.64 (t, *J* = 7.2 Hz, 3H) (Supporting Figure S32). ^13^C NMR (150 MHz, DMSO-*d*_6_): δ 168.50, 134.95, 126.98, 126.49, 121.52, 121.47, 120.07, 119.14, 115.83, 115.67, 115.53, 107.55, 76.28, 60.57, 45.56, 31.37, 24.15, 22.38, 14.87, 8.33 (Supporting Figure S33). LC-MS/MS [M – 1]^−^ 379.20 g/mol (%100) (Supporting Figure S34). Formula: C_23_H_25_FN_2_O_2._

***N-(4-Chlorophenyl)-2-(1,8-diethyl-1,3,4,9-tetrahydropyrano­[3,4-*** ***b*** ***]­indol-1-yl)­acetamide (ECL)*:** FT-IR Spectrum (cm^–1^): 3311 (N–H), 3050 (Ar C–H), 2965–2844 (Aliphatic C–H), 1673 (C=O) (Supporting Figure S35). ^1^H NMR (400 MHz, DMSO-*d*_6_) δ 10.54 (s, 1H), 9.92 (s, 1H), 7.60 (d, *J* = 8.3 Hz, 2H), 7.32 (d, *J* = 8.5 Hz, 2H), 7.23 (d, *J* = 7.4 Hz, 1H), 6.93–6.86 (m, 2H), 3.99 (s, 2H), 3.03 (d, *J* = 13.7 Hz, 2H), 2.88–2.82 (m, 2H), 2.66–2.61 (m, 2H), 2.08 (s, 2H), 1.25 (t, *J* = 7.3 Hz, 3H), 0.66 (t, *J* = 7.2 Hz, 3H) (Supporting Figure S36). ^13^C NMR (100 MHz, DMSO-*d*_6_) δ 168.80, 138.51, 136.73, 135.00, 128.98, 127.10, 127.04, 126.50, 121.26, 120.09, 119.16, 115.85, 107.61, 76.36, 60.57, 45.69, 40.63, 40.43, 40.22, 40.01, 39.80, 39.59, 39.38, 31.46, 24.18, 22.40, 14.90, 8.35 (Supporting Figure S37). QTOF-HRMS [M + H]^+^ 397.16661; [M + 3]^+^ 399.16499; [M + 4]^+^ 400.16676 (Supporting Figure S38). Formula: C_23_H_25_ClN_2_O_2_.

***N-(4-Bromophenyl)-2-(1,8-diethyl-1,3,4,9-tetrahydropyrano­[3,4-*** *b* ***]­indol-1-yl)­acetamide (ECM)*:** FT-IR Spectrum (cm^–1^): 3310 (N–H), 3057 (Ar C–H), 2965–2852 (Aliphatic C–H), 1673 (C=O) (Supporting Figure S39). ^1^H NMR (400 MHz, DMSO-*d*_6_) δ 10.57 (s, 1H), 9.96 (s, 1H), 7.56 (d, *J* = 8.4 Hz, 2H), 7.46 (d, *J* = 8.3 Hz, 2H), 7.23 (s, 1H), 6.90 (m, 2H), 4.00 (s, 2H), 3.05 (d, *J* = 13.7 Hz, 2H), 2.86 (d, *J* = 8.5 Hz, 2H), 2.64 (s, 2H), 2.08 (s, 2H), 1.30–1.21 (m, 3H), 0.66 (s, 3H) (Supporting Figure S40). ^13^C NMR (100 MHz, DMSO-*d*_6_) δ 168.84, 138.94, 136.72, 135.00, 131.89, 127.05, 126.49, 121.63, 115.85, 115.09, 107.61, 76.37, 45.73, 40.64, 40.43, 40.22, 40.01, 39.80, 39.59, 39.36, 31.49, 24.19, 22.41, 8.35 (Supporting Figure S41). LC-MS/MS [M + 1]^+^ 441.15 g/mol (%100); 442.20 (%17.93) (Supporting Figure S42). Formula: C_23_H_25_BrN_2_O_2_.

### 4.3 *In Vitro* Enzyme Activity

According to Ellman’s method, *in vitro* enzyme-activities of the synthesized compounds on AChE (*electric eel*) and BChE (from equine serum) were investigated.^60^ These enzymes were dissolved in DMSO at an initial concentration of 1 (mg/mL). Acetylthiocholine iodide (AChI) and butyrylcholine iodide (BChI) were used as substrates for enzymatic reactions. For this purpose, (0.1 mL) of Tris/HCl buffer (pH 8.0, 1.0 M) and different concentrations of the compounds (10–30 μg/mL) were added to a 50 μL solution of AChE and BChE (5.30 × 10^–3^ EU). The mixture was incubated at 20 °C for 20 min. Subsequently, 50 μL of 5,5′-dithio-bis­(2-nitro-benzoic acid) (DTNB, 0.5 mM) and AChI/BChI were added, and enzymatic reactions were initiated. The enzyme inhibition activities were measured at 412 nm using a spectrophotometer.^28,36,38,61^

### 4.4 Cell Culture and Cytotoxicity Assay

MTT assay was used to determine the cytotoxicity of the synthesized compounds.^62,63^ Human TNBC (MDA-MB-231, BT-20, BT-549), estrogen receptor positive (ER+) (MCF-7), and colon cancer (HT-29) cell lines were cultured in DMEM containing 2 mM l-Glutamine, 10% FBS, and 1% PS. The cultures were maintained in a 37 °C incubator with 5% CO_2_. All experiments were performed with multiple replicates. The cytotoxic effects of the synthesized compounds were determined using the MTT reagent. The cells were seeded into a 96-well plate, and the drugs were applied to the cells at increasing doses up to 100 μM for 72 h. The absorbances were measured at 570 nm in a plate reader. DMSO was used as a control.

### 4.5 Statistical Analysis

Statistical analysis was performed by using GraphPad Prism software and significant results of *p* values were evaluated.

### 4.6 *In Silico* Analyses

#### 4.6.1 Molecular Docking and MD Simulations

The studies were performed using Maestro Schrödinger (Schrödinger Release LLC, New York, NY). Default settings were used. Compounds were prepared using the LigPrep module of the Maestro. Protonation states at neutral pH 7 were determined by the Epic.^64^ Optimized Potentials for Liquid Simulations 4 (OPLS4) force field was used to determine the low energy conformations of the compounds in geometry optimization studies.^65^ For molecular docking studies, the crystal structures of cholinesterases (AChE and BChE) were downloaded from Protein Data Bank (https://www.rcsb.org/) (PDBs: 4EY7 for AChE and 6I0C for BChE). The Protein Preparation Wizard module and the OPLS4 force field were used to prepare the proteins. Water molecules were removed from the protein, and missing hydrogen atoms were added, and the proteins were prepared at physiological pH 7.4. To determine how ligands with a 3D structure bind to the active site of the protein, the binding site was determined based on the cocrystallized ligand using the Receptor Grid Generation module. Molecular docking was performed using the Glide/SP method. Subsequently, the top docking poses of the complexes were used for MD simulation analyses performed by Desmond over a 100 ns period.^66^ The complex solution was performed using the TIP3P water model. Sodium and chloride ions were used to neutralize the system at 0.15 M. The ensemble class, temperature, and pressure were selected as NPT, 300 K, and 1.01325 bar, respectively. The isotropic Martyna–Tobias–Klein barostat and the Nose–Hoover thermostat methods were used to control the temperature and pressure of the system, respectively. The interactions between the protein and ligand were obtained using a simulation interactions diagram.^67^

#### 4.6.2 Binding Free Energy Analysis

Prime method^68^ of Maestro was used to determine binding free energy values (ΔG_bind_) of the protein–ligand complexes. The molecular mechanics generalized Born surface area (MM/GBSA) approach uses the VSGB solvation model. Frames were extracted from MD simulation trajectories every 10 ns to obtain average binding free energies.

#### 4.6.3 *In Silico* ADMET

*In silico* ADMET estimation, pharmacokinetic and toxicity profiles of the synthesized compounds were performed using BIOVIA Discovery Studio 2022.^67^ TOPKAT Ames mutagenicity and carcinogenicity, hepatotoxicity, and BBB penetration were evaluated for the compounds.

## Supplementary Material



## Data Availability

The data underlying this study are available in the published article and its online Supporting Information.

## Notes

Characterization data for the synthesized compounds and *in silico* toxicity prediction (PDF)

## Abbreviations

(AD): Alzheimer’s disease
(AChE): Acetylcholinesterase
(ACh): Acetylcholine
ADMET: (Absorption, distribution, metabolism, excretion, toxicity)
(AChI): Acetylthiocholine iodide
(BBB): Blood–brain barrier
(BChE): Butyrylcholinesterase
(BChI): Butyrylcholine iodide
(^13^C NMR): Carbon-13 nuclear magnetic Resonance
(CAS): Catalytic active site
(CNS): Central nervous system
(ChAT): Choline acetyltransferase
(DMF): Dimethylformamide
(DTNB): 5,5′-Dithio-bis­(2-nitro-benzoic acid)
(d): Doublet
(dd): Doublet of doublets
(Dox): Doxorubicin
(HOBt.H_2_O): 1-Hydroxybenzotriazole hydrate
(ER+): Estrogen receptor positive
(EtOAc): Ethyl acetate
(EDCI.HCl): 1-Ethyl-3-(3-(dimethylamino)­propyl)­carbodiimide hydrochloride
(eEF2K): Eukaryotic elongation factor 2 kinase
(FBS): Fetal bovine Serum
(FT-IR): Fourier-transform infrared
(HBA): Hydrogen bond acceptors
(HBD): Hydrogen bond donors
(LC-MS/MS): Liquid chromatography tandem mass spectrometry
(Ro5): Lipinski’s rule of five
(MS): Mass spectrometry
(μM): Micromolar
(MD): Molecular dynamics
(MM/GBSA): Molecular mechanics/generalized born surface area
(MWs): Molecular weights
(m): Multiplet
(MTDLs): Multiple target directed ligands
MTT: (3-(4,5-dimethylthiazol-2-yl)-2,5-diphenyltetrazolium bromide)
(nM): Nanomolar
(NSAID): Nonsteroidal anti-inflammatory drug
(OPLS4): Optimized potentials for liquid simulations 4
(ppm): Parts per million
(PS): Penicillin/Streptomycin
(PAS): Peripheral anionic site
(Prep TLC): Prep thin layer chromatography
(^1^H NMR): Proton nuclear magnetic resonance
(QTOF-HRMS): Quadrupole time-of-flight high-resolution mass Spectrometry
(qd): Quartet of doublets
(RMSD): Root-mean-square deviation
(RMSF): Root-mean-square fluctuation
(s): Singlet
(TLC): Thin layer chromatography
(t): Triplet
(TNBC): Triple negative breast cancer
