# Supplementary material for: Design, Synthesis, In Vitro Biological Evaluation, and In Silico Analyses of Novel Pyrano[3,4‑b]indole-Containing Compounds as Promising Multitarget Candidates for Alzheimer’s Disease and Cancer Therapy
Source: ACS Omega. 2026 Aug 26;11(35):52714–30. doi: 10.1021/acsomega.6c03670 (PMC13563997; doi:10.1021/acsomega.6c03670)
Supplement: Supplementary file 1 [file ao6c03670_si_001.pdf]

Supporting Information

Design, Synthesis, In Vitro Biological Evaluation, and In Silico Analyses of Novel Pyrano[3,4-b]indole containing Compounds as Promising Multitarget Candidates for Alzheimer's Disease and Cancer Therapy

Ferah Comert Onder[a,b,*], Alper Onder[b], Kadircan Ural[c], Merve Sıkık[c], Khaled A.N. Abusharkh[b,d], Bulent Ozpolat[e], Mehmet Ay[b]

[a]Department of Medical Biology, Faculty of Medicine, Çanakkale Onsekiz Mart University, Çanakkale, 17020, Türkiye

[b]Natural Products and Drug Research Laboratory, Department of Chemistry, Faculty of Science, Çanakkale Onsekiz Mart University, Çanakkale, 17020, Türkiye

[c]Department of Medical System Biology, School of Graduate Studies, Çanakkale Onsekiz Mart University, Çanakkale, 17020, Türkiye

[d]Department of Chemistry and Chemical Technology, Faculty of Science and Technology, Al-Quds University, East Jerusalem, Palestine

[e]Stephenson School of Biomedical Engineering, The University of Oklahoma, Norman, OK 73019, USA

Correspondence to Ferah Comert Onder. Department of Medical Biology, Faculty of Medicine, Çanakkale Onsekiz Mart University, Çanakkale, 17020, Türkiye

Email: ferahcomertonder@comu.edu.tr

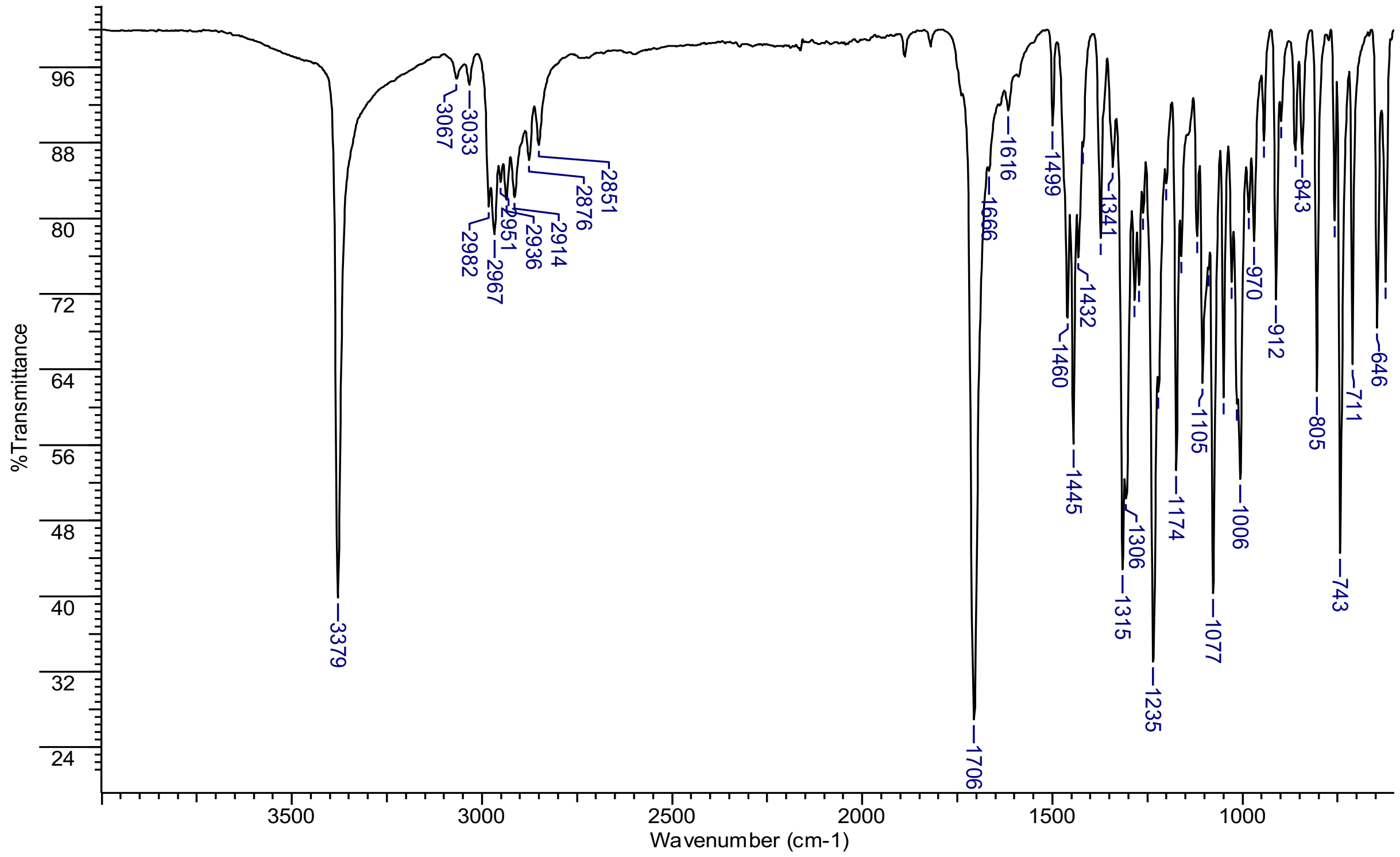

Figure S1. FTIR Spectrum for 1-(4-benzylpiperazin-1-yl)-2-(1,8-diethyl-1,3,4,9-tetrahydropyrano[3,4-b]indol-1-yl)ethan-1-one (**ECA**)

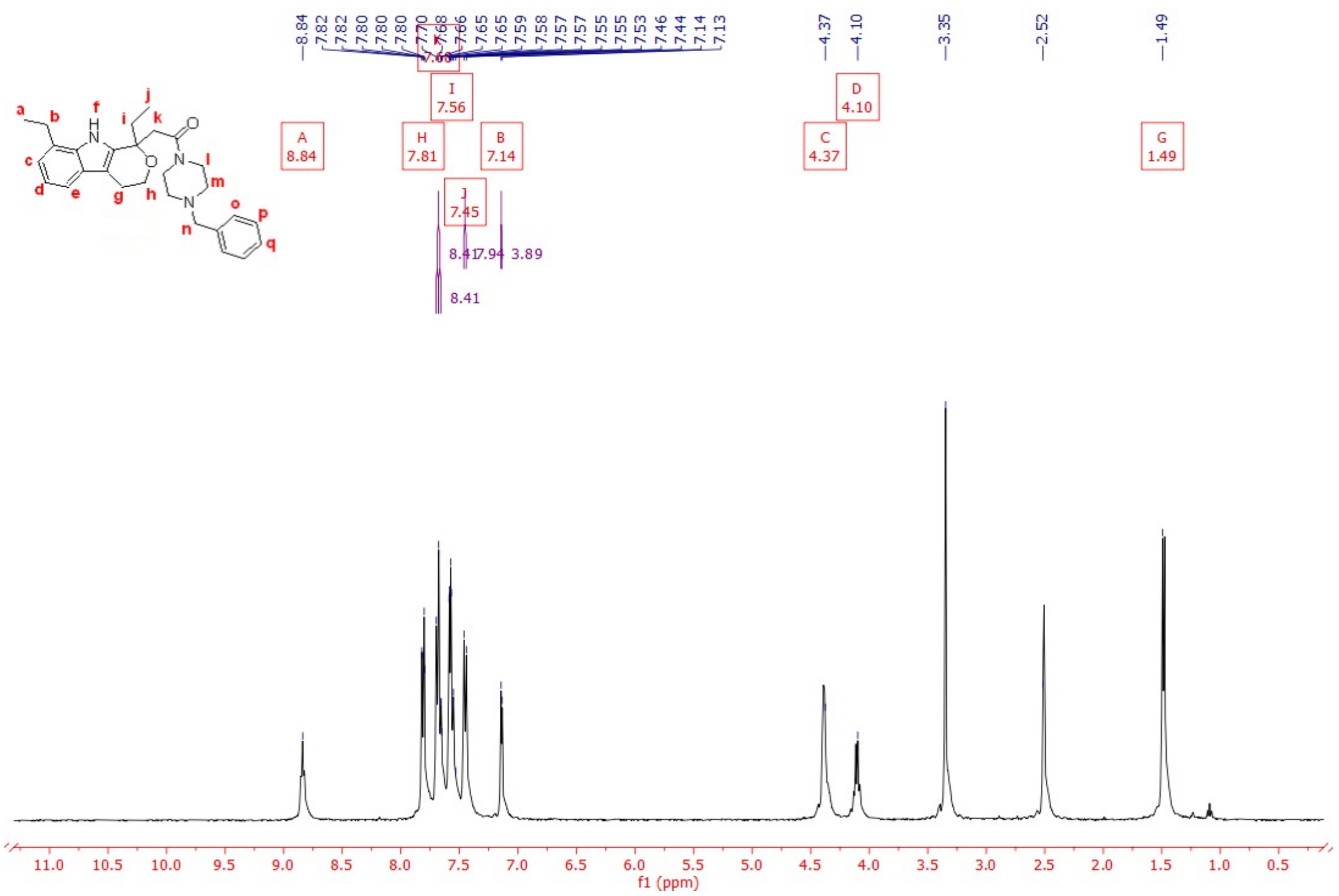

Figure S2. ^{1}H NMR Spectrum for 1-(4-benzylpiperazin-1-yl)-2-(1,8-diethyl-1,3,4,9-tetrahydropyrano[3,4-b]indol-1-yl)ethan-1-one (**ECA**)

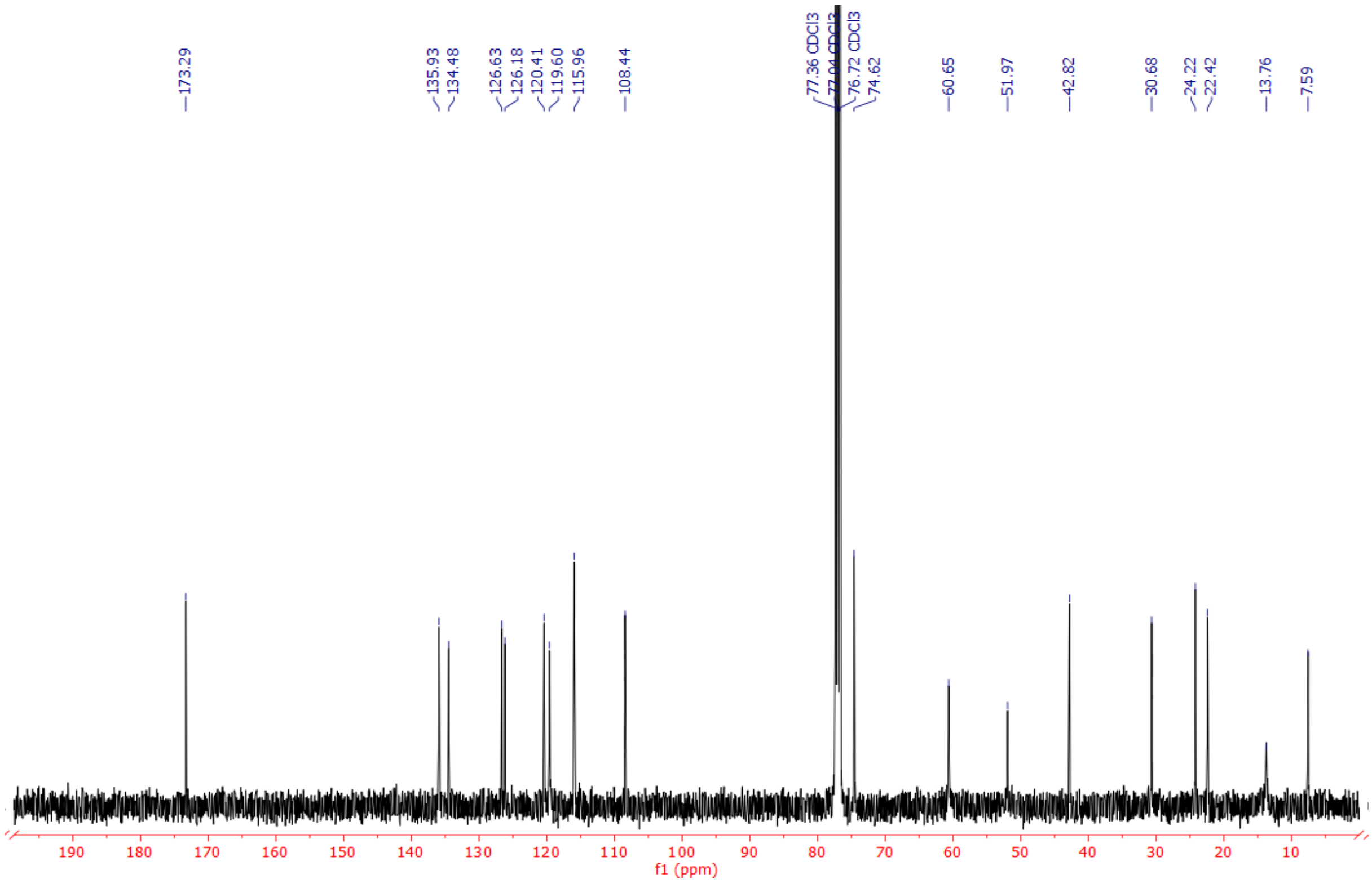

Figure S3. ^{13}C NMR Spectrum for 1-(4-benzylpiperazin-1-yl)-2-(1,8-diethyl-1,3,4,9-tetrahydropyrano[3,4-b]indol-1-yl)ethan-1-one (**ECA)**

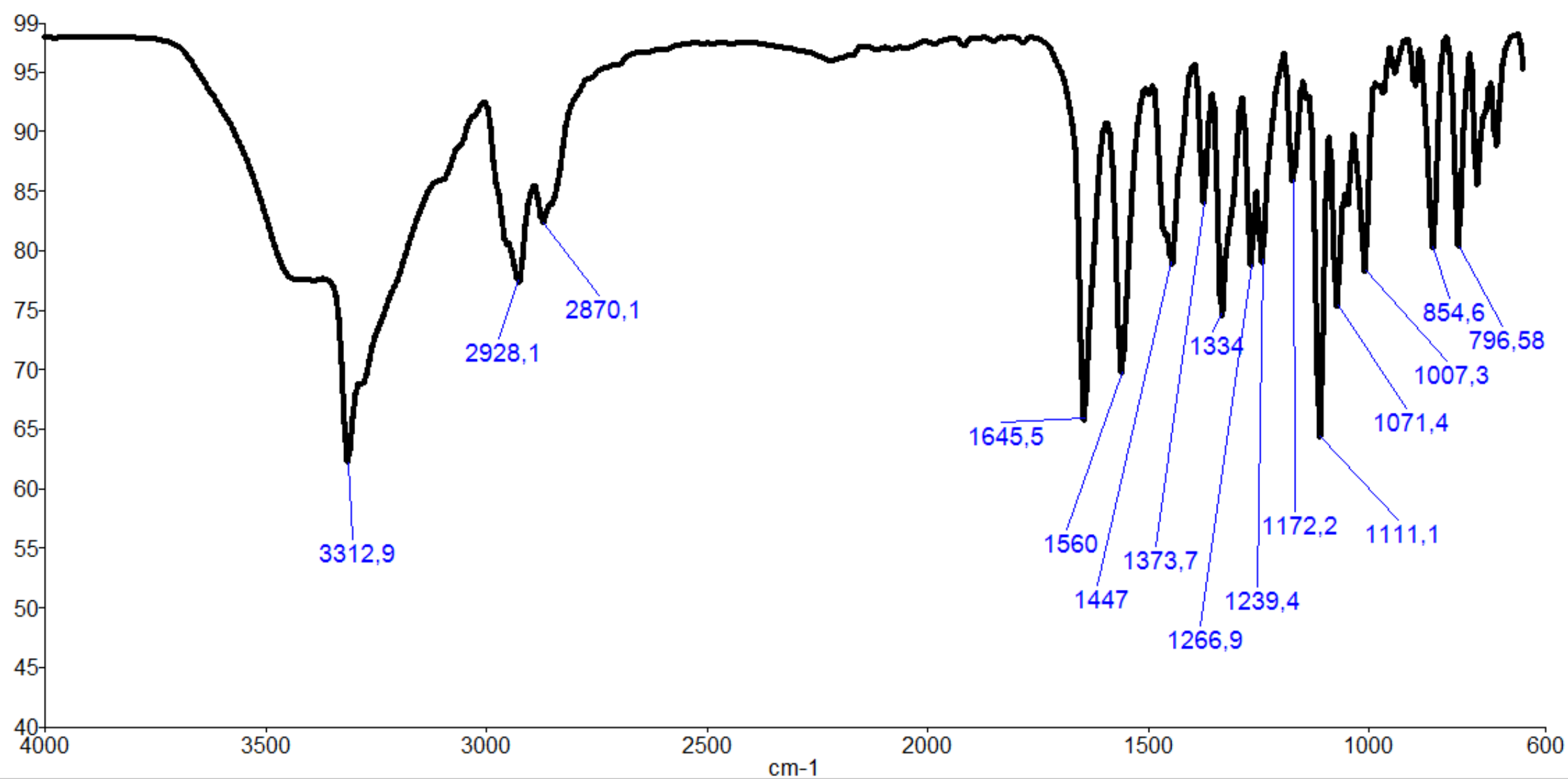

Figure S4. FTIR Spectrum for 2-(1,8-diethyl-1,3,4,9-tetrahydropyrano[3,4-b]indol-1-yl)-*N*-(3-morpholinopropyl)acetamide (**ECB**)

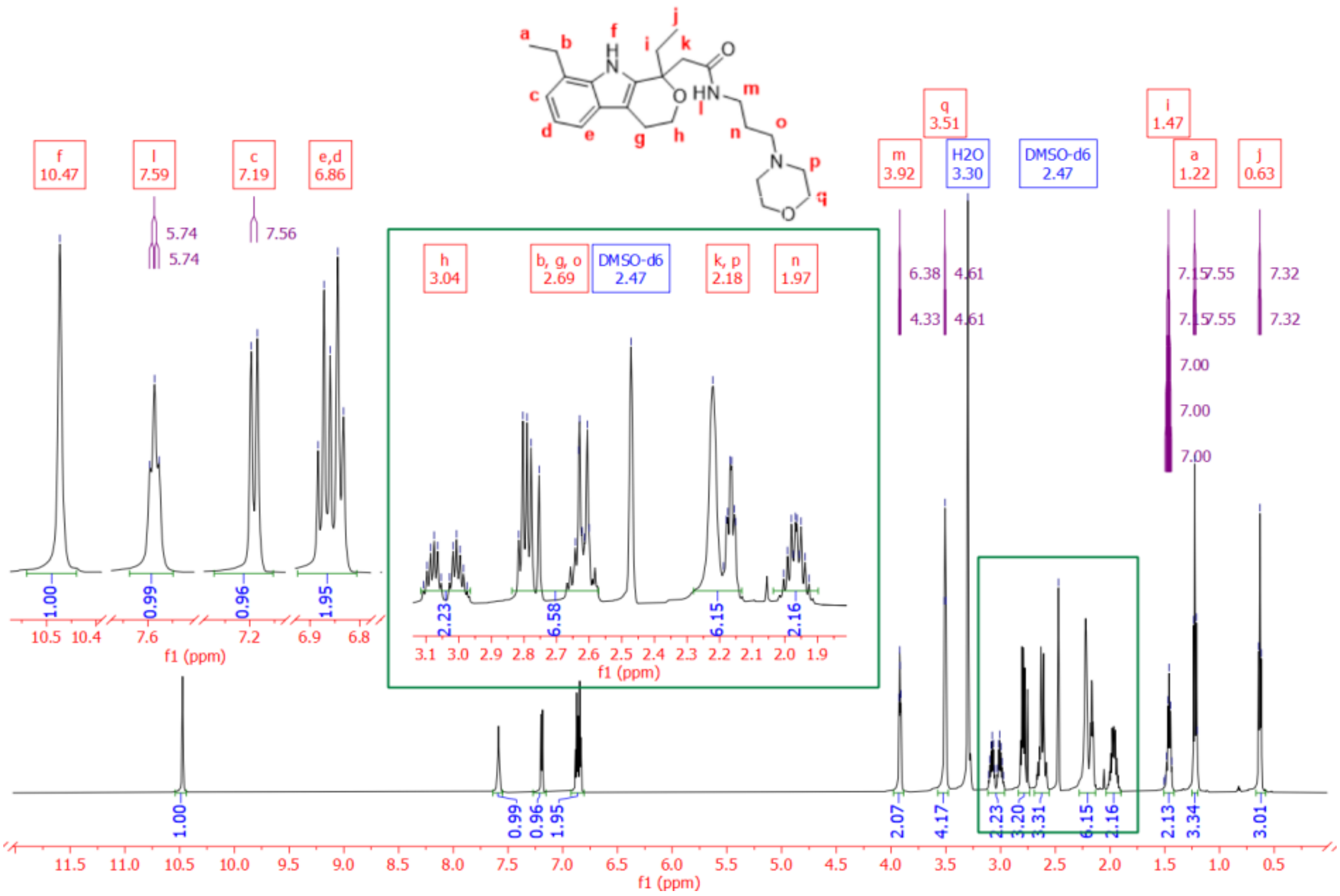

Figure S5. ^{1}H NMR Spectrum for 2-(1,8-diethyl-1,3,4,9-tetrahydropyrano[3,4-b]indol-1-yl)-*N*-(3-morpholinopropyl)acetamide (**ECB**)

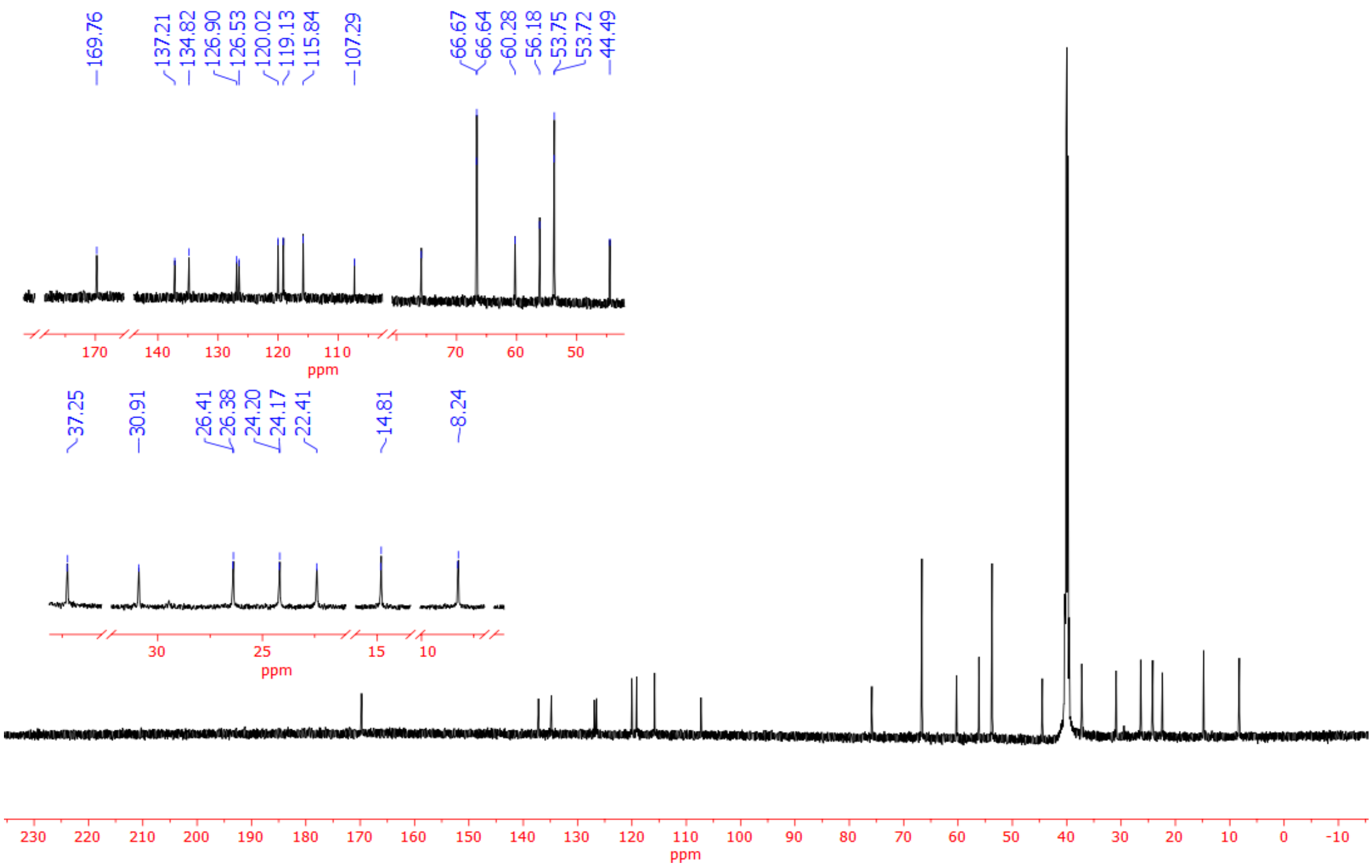

Figure S6. ^{13}C NMR Spectrum for 2-(1,8-diethyl-1,3,4,9-tetrahydropyrano[3,4-b]indol-1-yl)-*N*-(3-morpholinopropyl)acetamide (**ECB**)

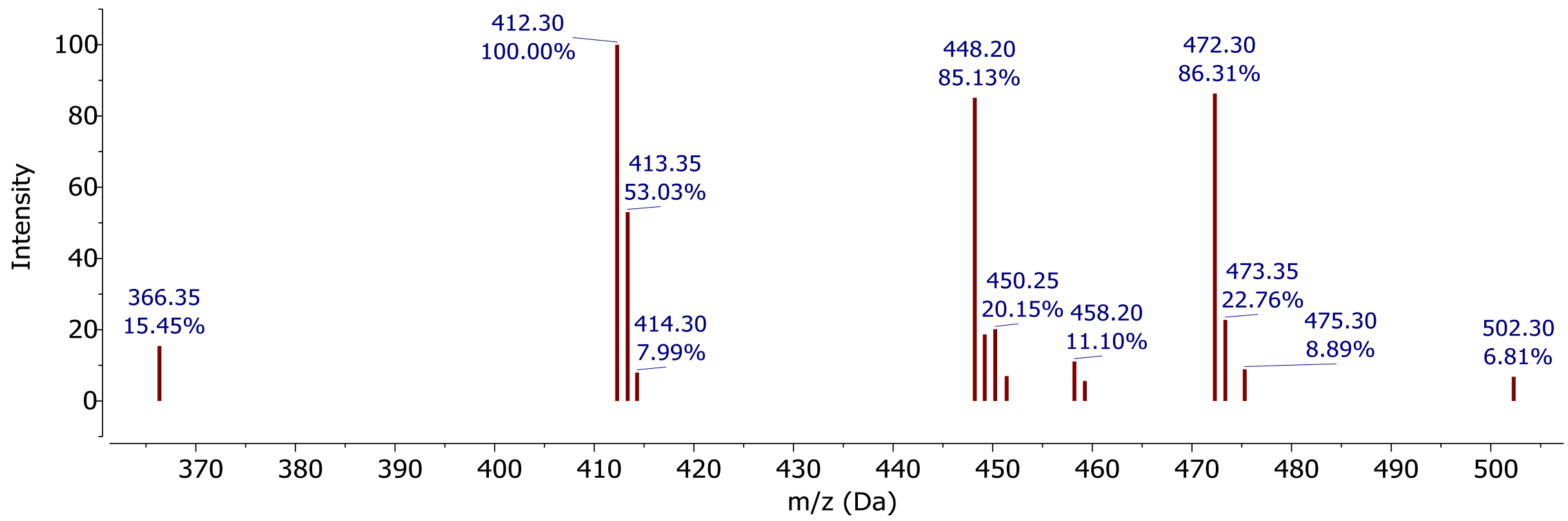

Figure S7. LC-MS/MS Spectrum for 2-(1,8-diethyl-1,3,4,9-tetrahydropyrano[3,4-b]indol-1-yl)-*N*-(3-morpholinopropyl)acetamide (**ECB**)

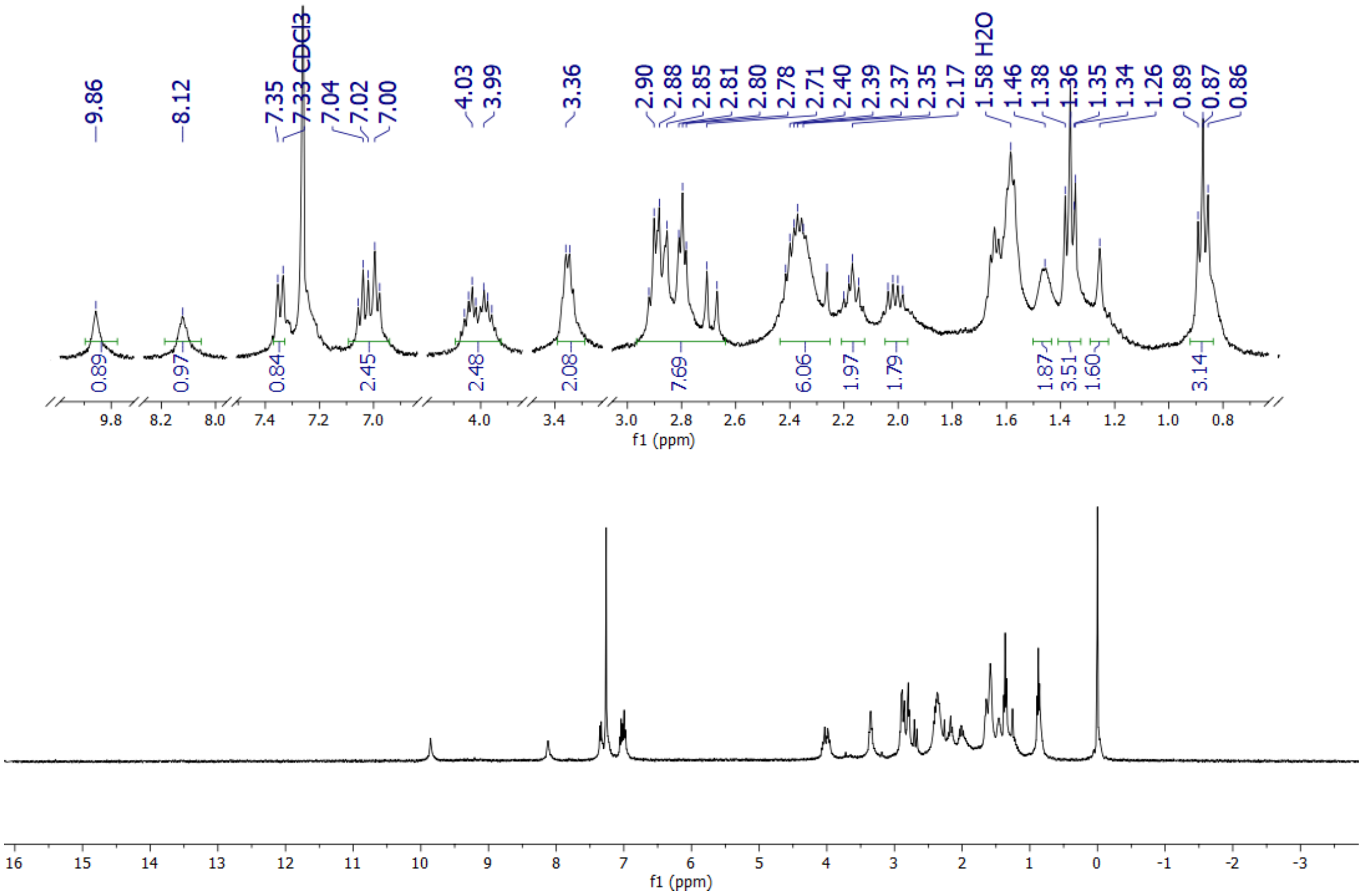

Figure S8. ^{1}H NMR Spectrum for 2-(1,8-diethyl-1,3,4,9-tetrahydropyrano[3,4-b]indol-1-yl)-*N*-(3-(piperidin-1-yl)propyl) acetamide (**ECC**)

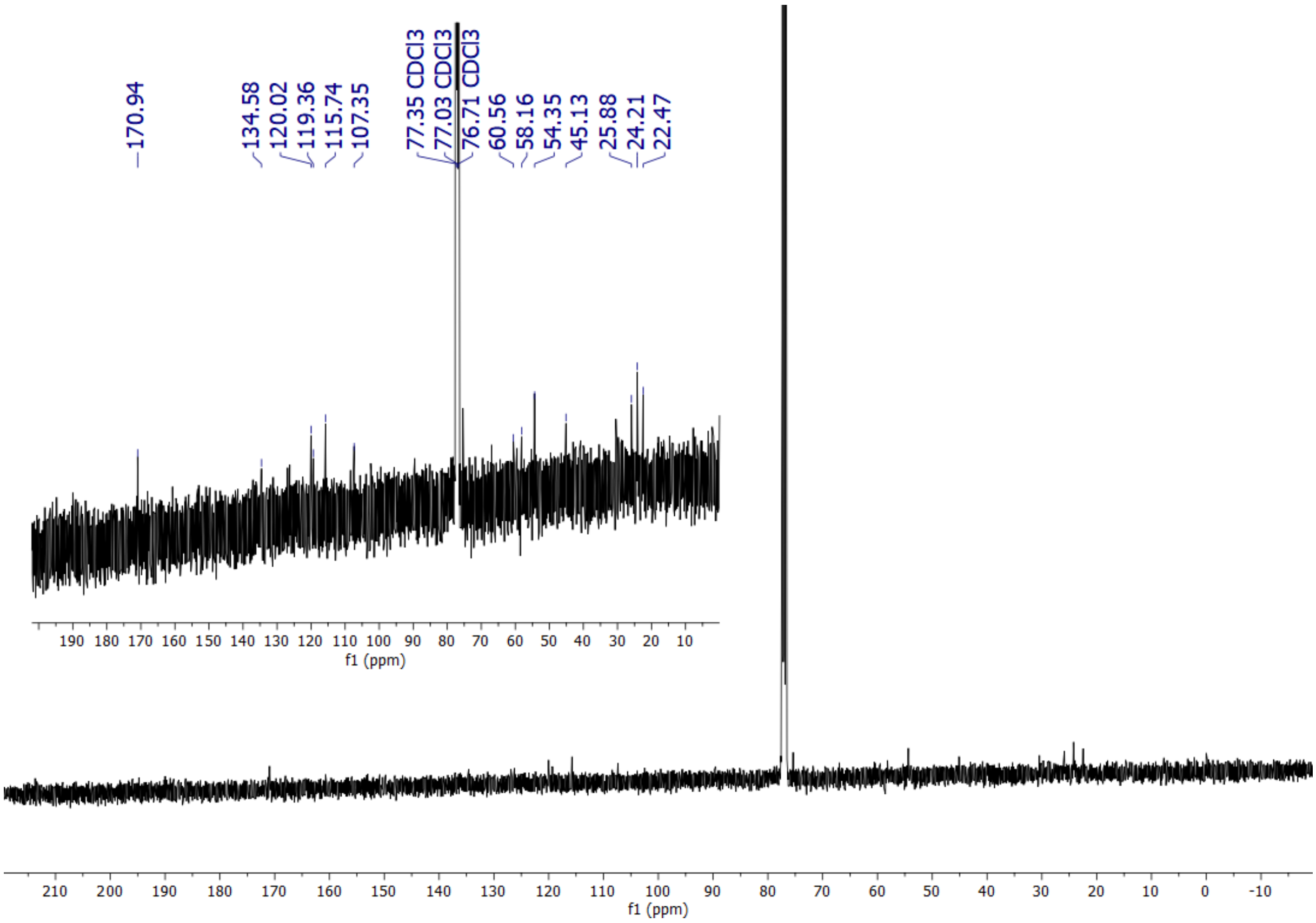

^{13}C NMR Spectrum for 2-(1,8-diethyl-1,3,4,9-tetrahydropyrano[3,4-b]indol-1-yl)-*N*-(3-(piperidin-1-yl)propyl) acetamide (**ECC**)

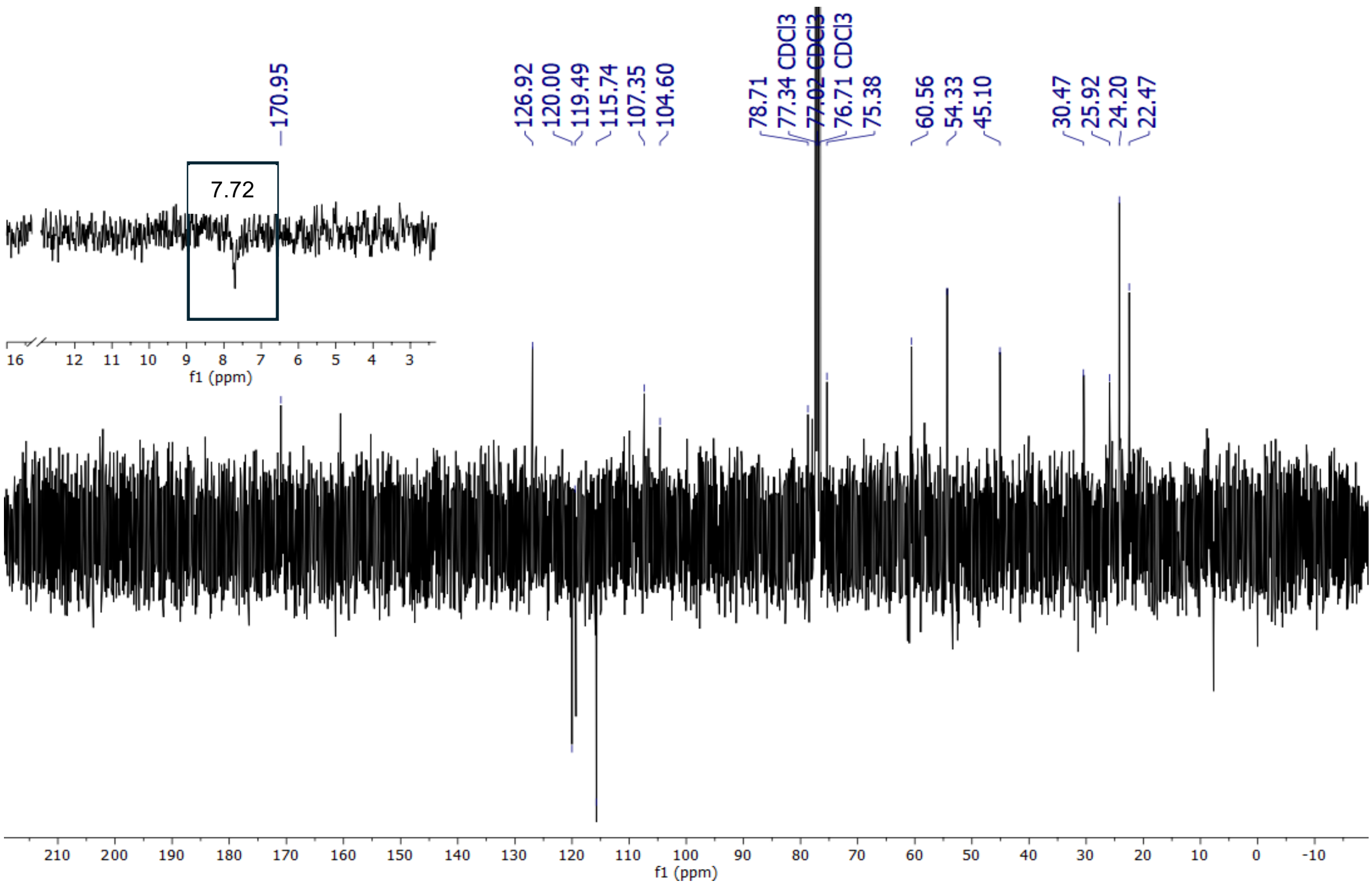

Figure S9. ^{13}C NMR (APT) Spectrum for 2-(1,8-diethyl-1,3,4,9-tetrahydropyrano[3,4-b]indol-1-yl)-*N*-(3-(piperidin-1-yl)propyl) acetamide (**ECC**)

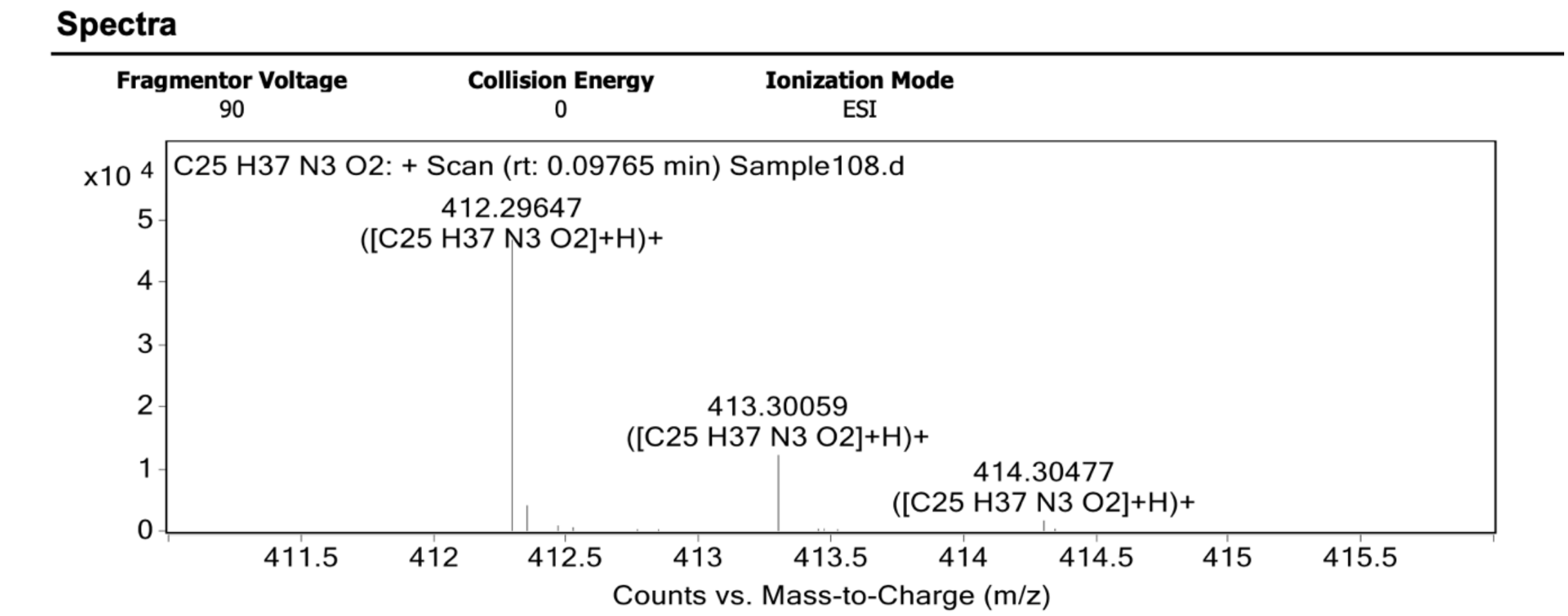

Figure S10. QTOF-HRMS Spectrum for 2-(1,8-diethyl-1,3,4,9-tetrahydropyrano[3,4-b]indol-1-yl)-*N*-(3-(piperidin-1-yl)propyl) acetamide (**ECC**)

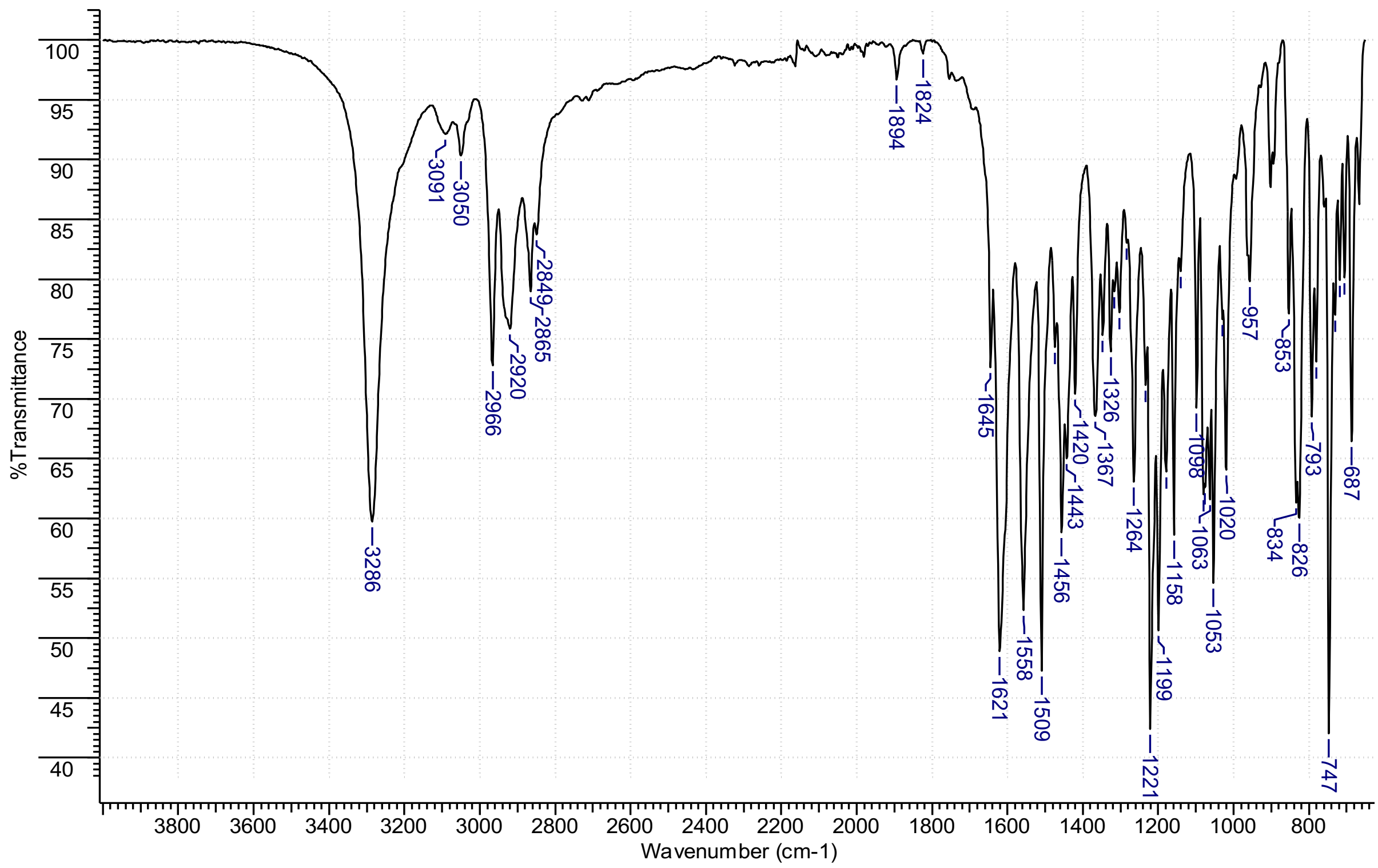

Figure S11. FTIR Spectrum for 2-(1,8-diethyl-1,3,4,9-tetrahydropyrano[3,4-b]indol-1-yl)-*N*-(4-fluorobenzyl)acetamide (**ECD**)

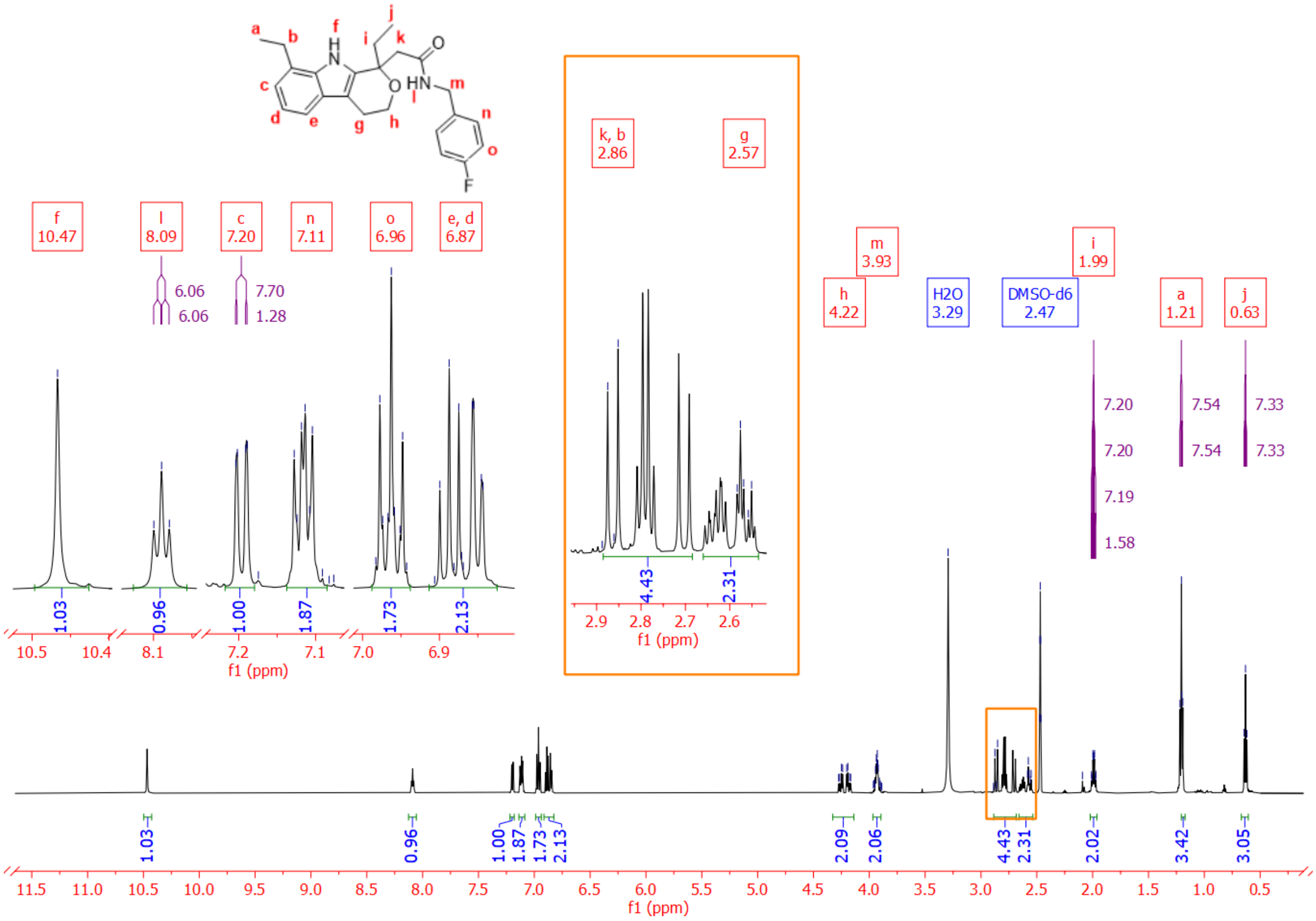

Figure S12. ^{1}H NMR Spectrum for 2-(1,8-diethyl-1,3,4,9-tetrahydropyrano[3,4-b]indol-1-yl)-*N*-(4-fluorobenzyl)acetamide (**ECD**)

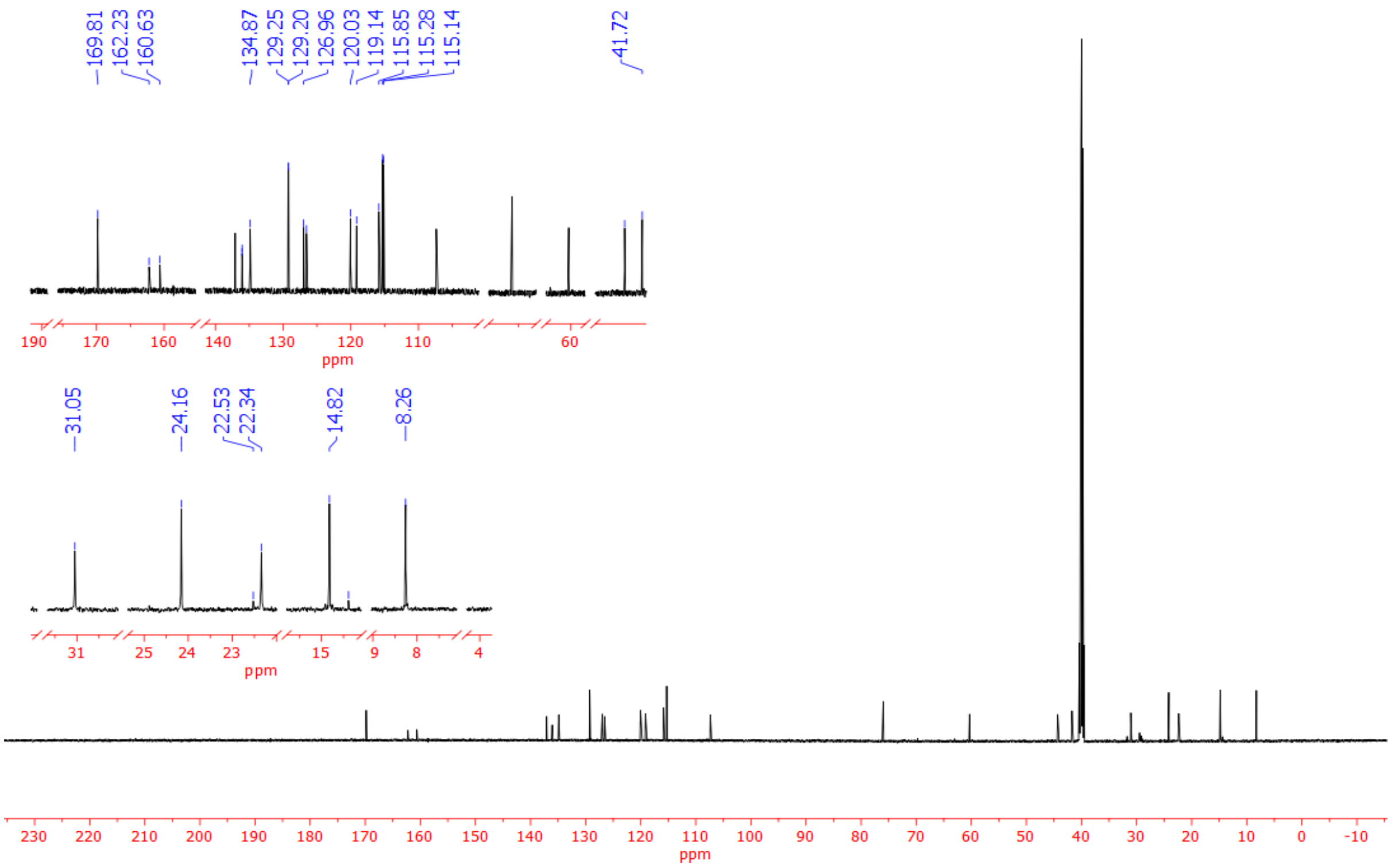

Figure S13. ^{13}C NMR Spectrum for 2-(1,8-diethyl-1,3,4,9-tetrahydropyrano[3,4-b]indol-1-yl)-*N*-(4-fluorobenzyl)acetamide (**ECD**)

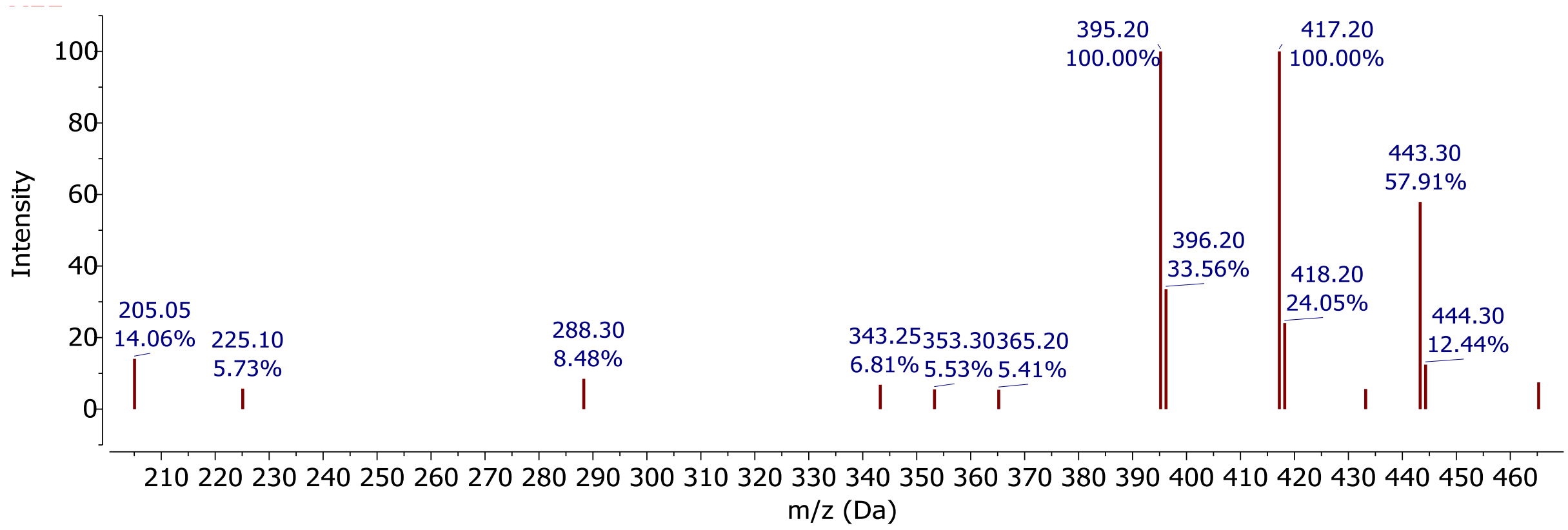

Figure S14. LC-MS/MS Spectrum for 2-(1,8-diethyl-1,3,4,9-tetrahydropyrano[3,4-b]indol-1-yl)-*N*-(4-fluorobenzyl)acetamide (**ECD**)

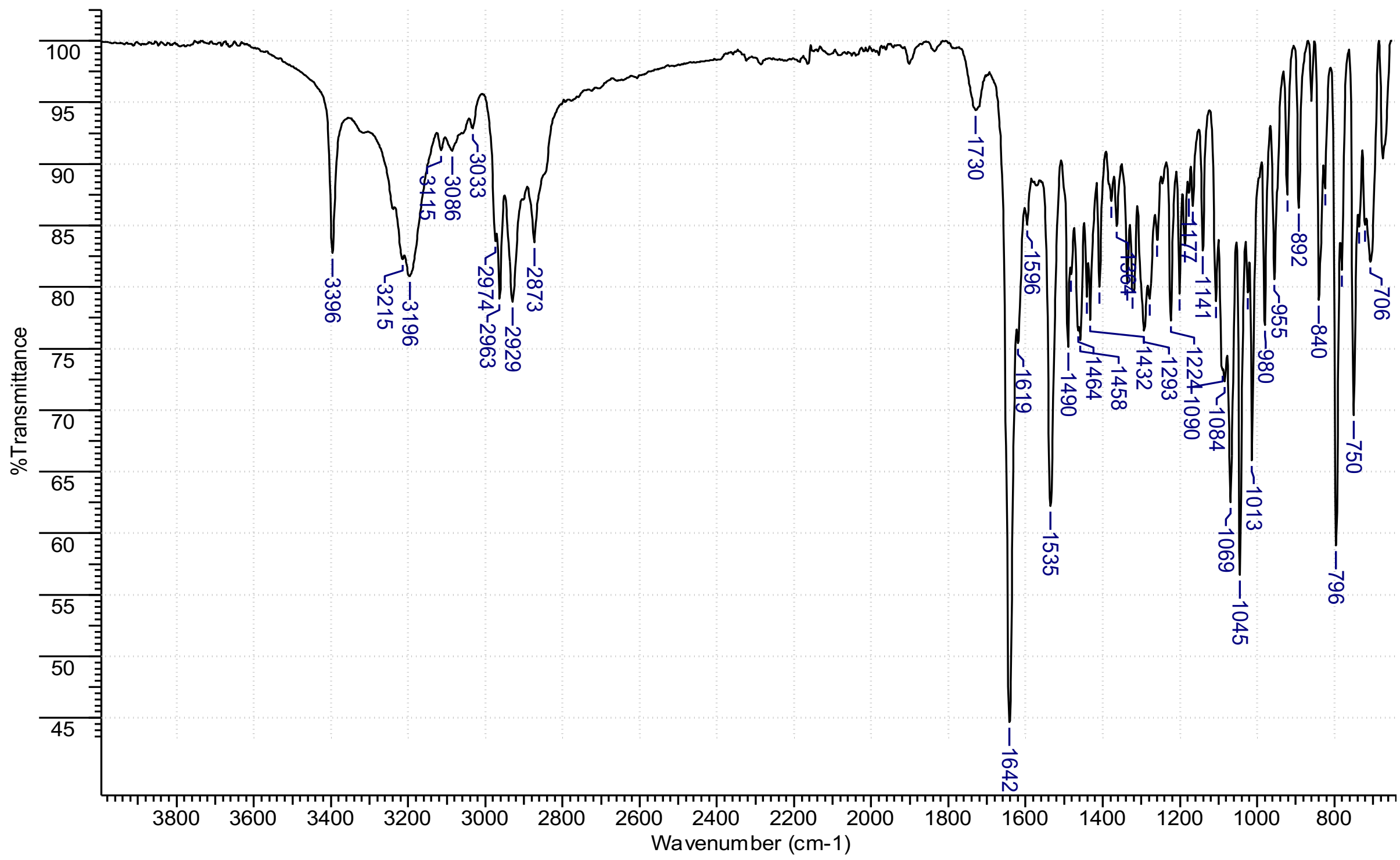

Figure S15. FTIR Spectrum for *N*-(4-chlorobenzyl)-2-(1,8-diethyl-1,3,4,9-tetrahydropyrano[3,4-b]indol-1-yl)acetamide (**ECE**)

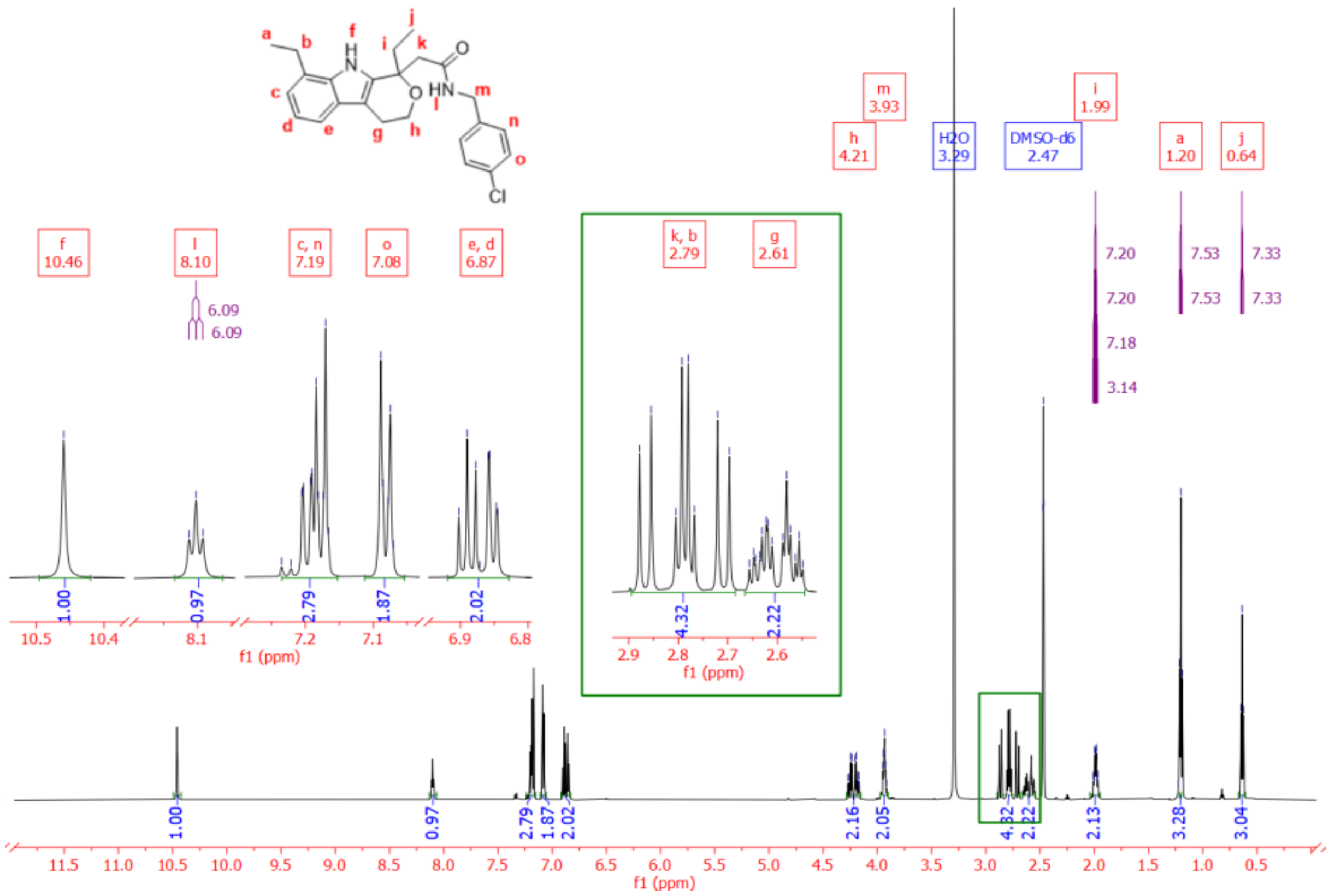

Figure S16. ^{1}H NMR Spectrum for *N*-(4-chlorobenzyl)-2-(1,8-diethyl-1,3,4,9-tetrahydropyrano[3,4-b]indol-1-yl)acetamide (**ECE**)

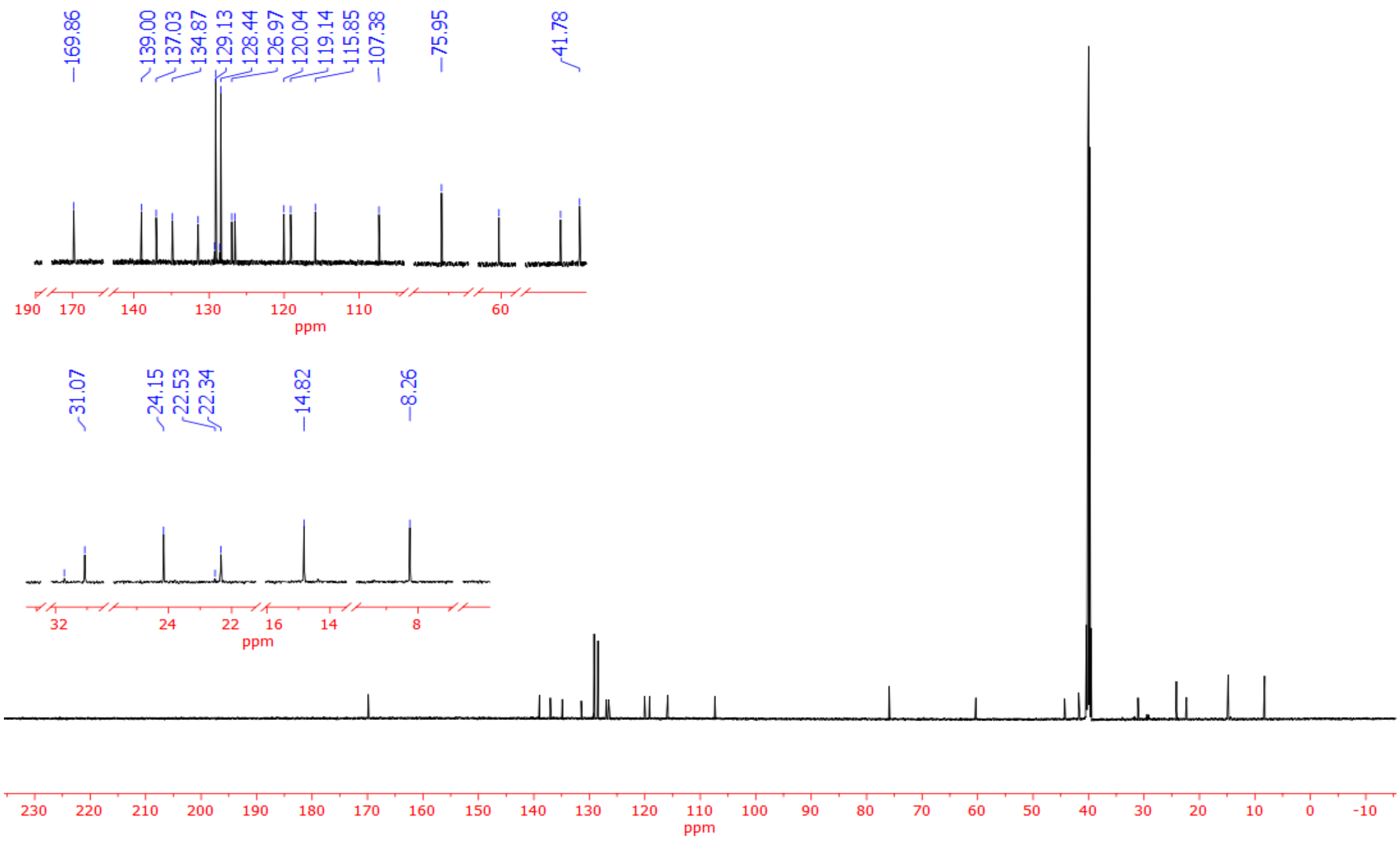

Figure S17. ^{13}C NMR Spectrum for *N*-(4-chlorobenzyl)-2-(1,8-diethyl-1,3,4,9-tetrahydropyrano[3,4-b]indol-1-yl)acetamide (**ECE**)

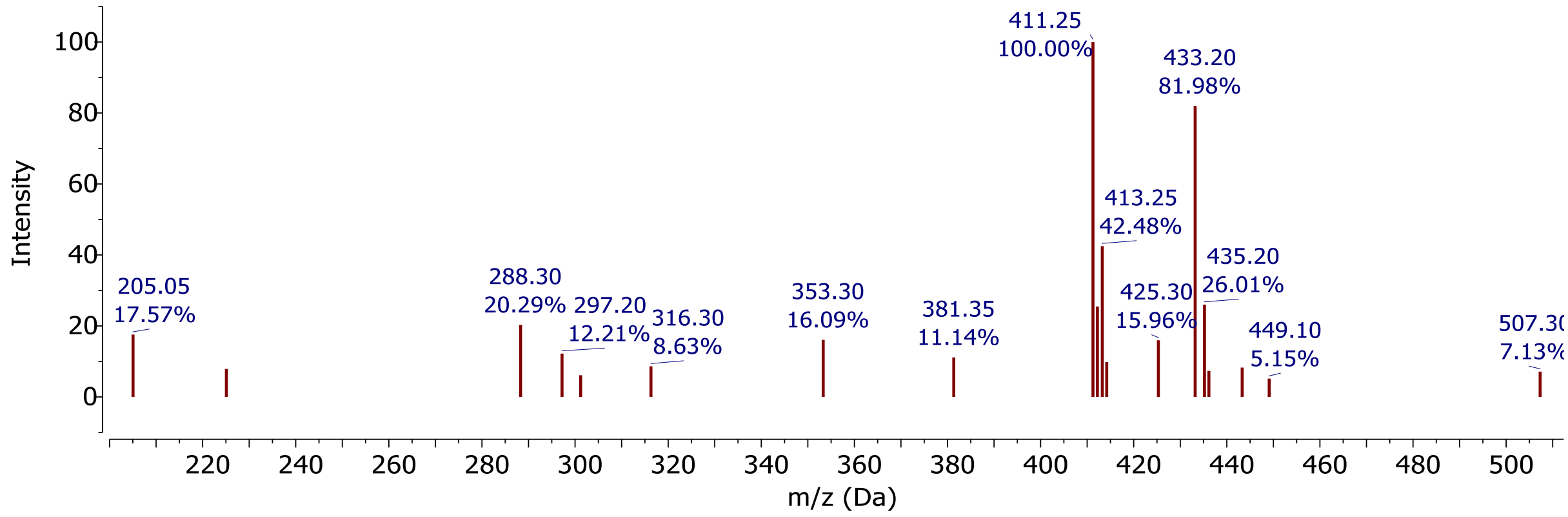

Figure S18. LC-MS/MS Spectrum for *N*-(4-chlorobenzyl)-2-(1,8-diethyl-1,3,4,9-tetrahydropyrano[3,4-b]indol-1-yl)acetamide (**ECE**)

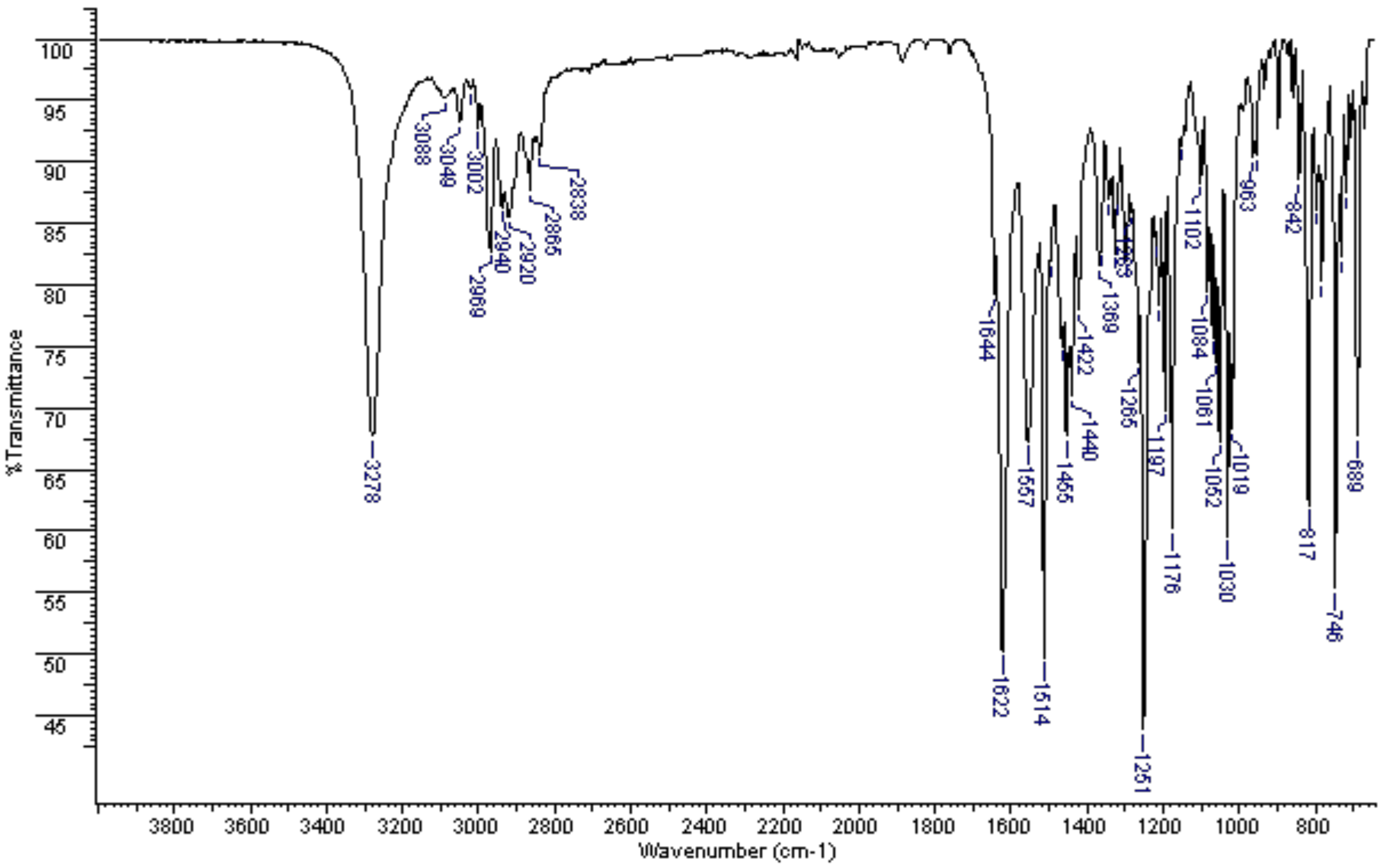

Figure S19. FTIR Spectrum for 2-(1,8-diethyl-1,3,4,9-tetrahydropyrano[3,4-b]indol-1-yl)-*N*-(4-methoxybenzyl)acetamide (**ECF**)

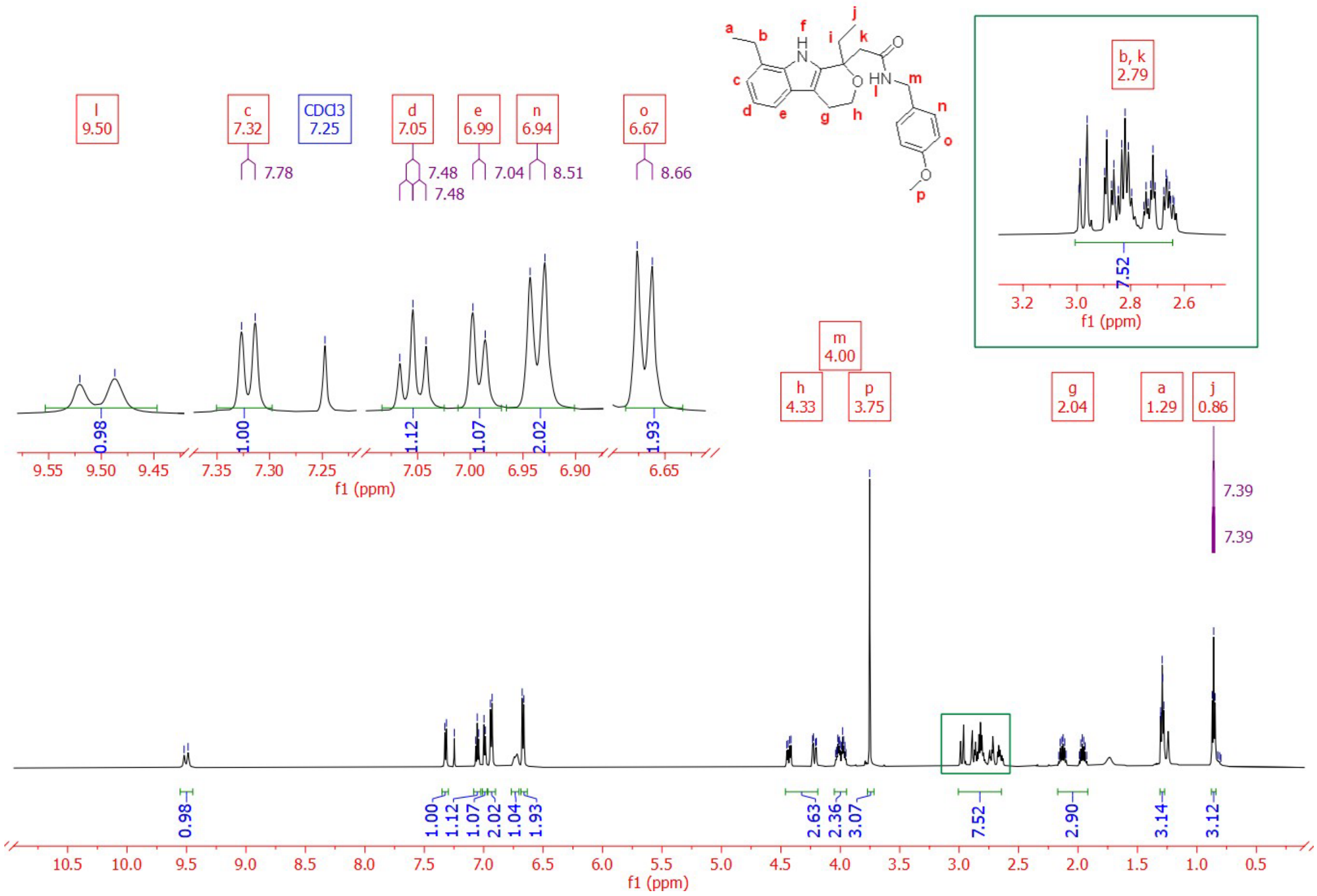

Figure S20. ^{1}H NMR Spectrum for 2-(1,8-diethyl-1,3,4,9-tetrahydropyrano[3,4-b]indol-1-yl)-*N*-(4-methoxybenzyl)acetamide (**ECF**)

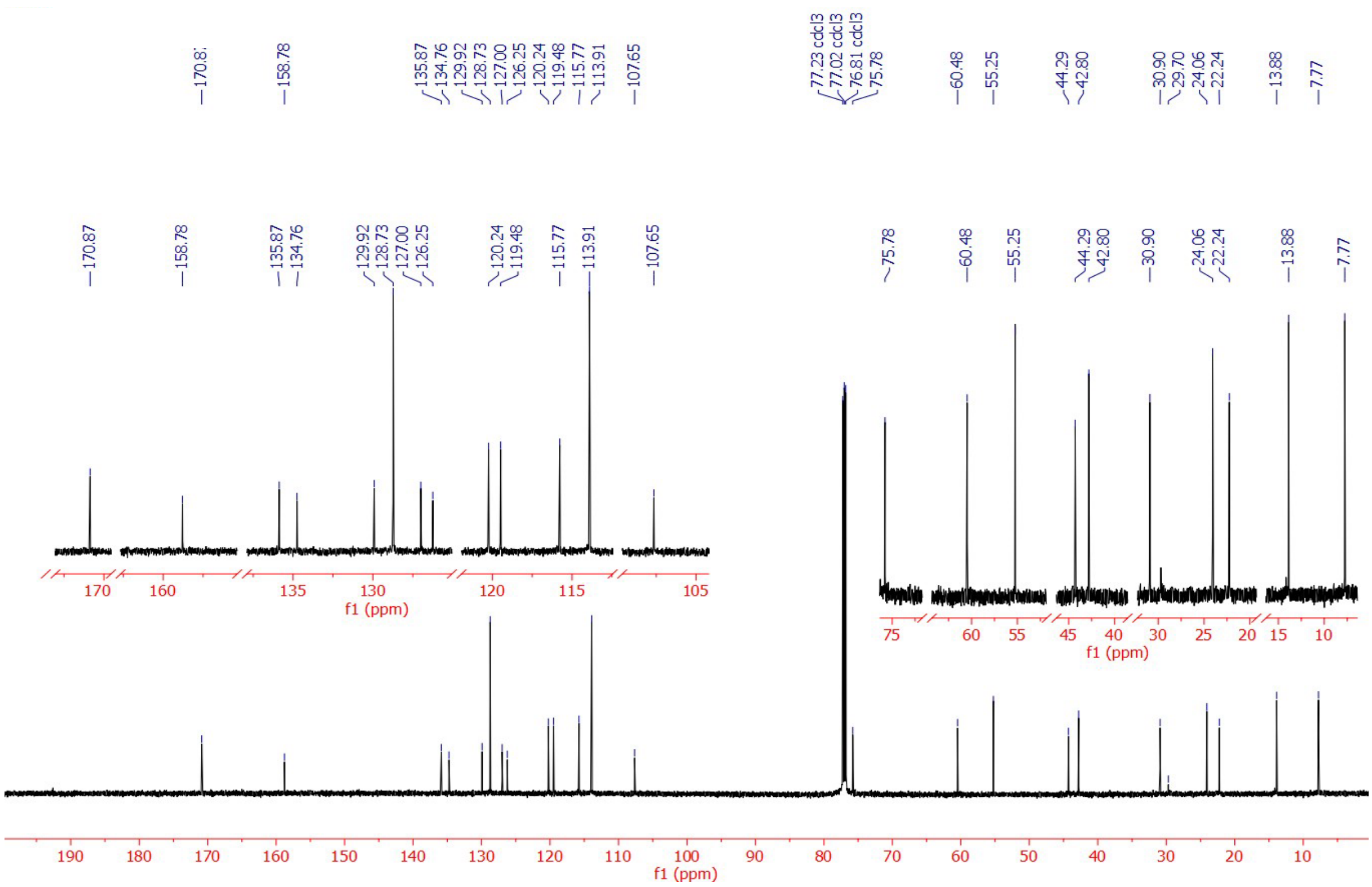

Figure S21. ^{13}C NMR Spectrum for 2-(1,8-diethyl-1,3,4,9-tetrahydropyrano[3,4-b]indol-1-yl)-*N*-(4-methoxybenzyl)acetamide (**ECF**)

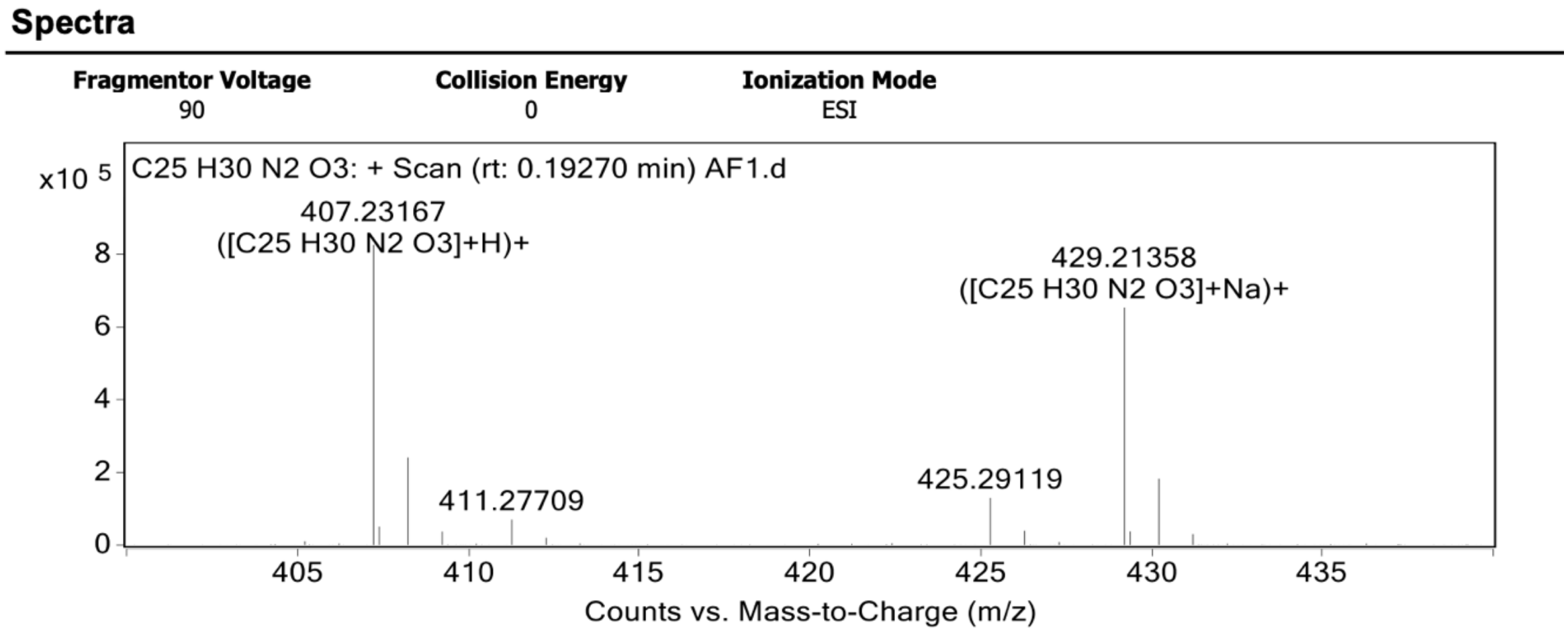

Figure S22. QTOF HRMS Spectrum for 2-(1,8-diethyl-1,3,4,9-tetrahydropyrano[3,4-b]indol-1-yl)-*N*-(4-methoxybenzyl)acetamide (**ECF**)

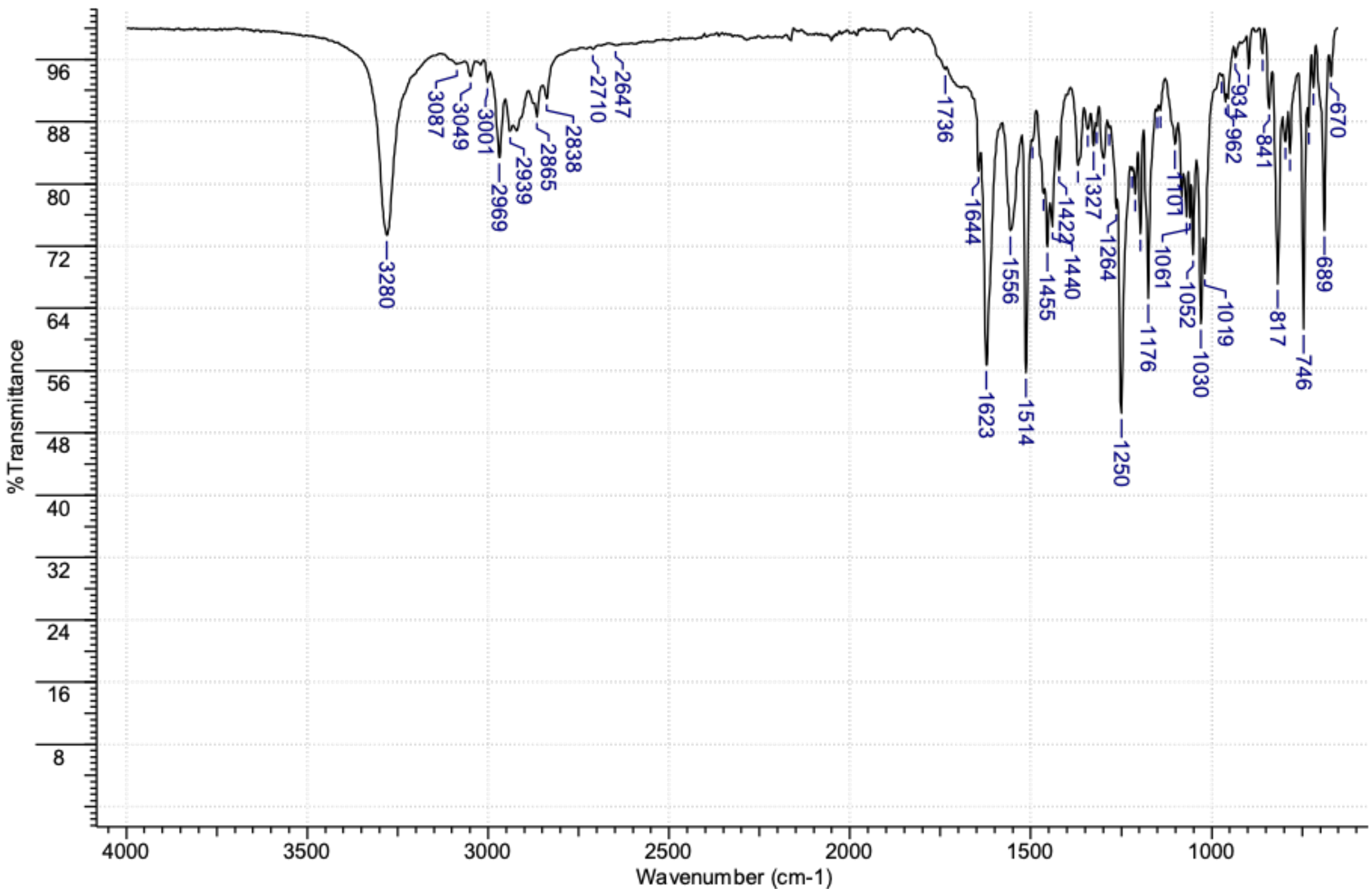

Figure S23. FTIR Spectrum for 2-(1,8-diethyl-1,3,4,9-tetrahydropyrano[3,4-b]indol-1-yl)-*N*-(4-methylbenzyl)acetamide (**ECG**)

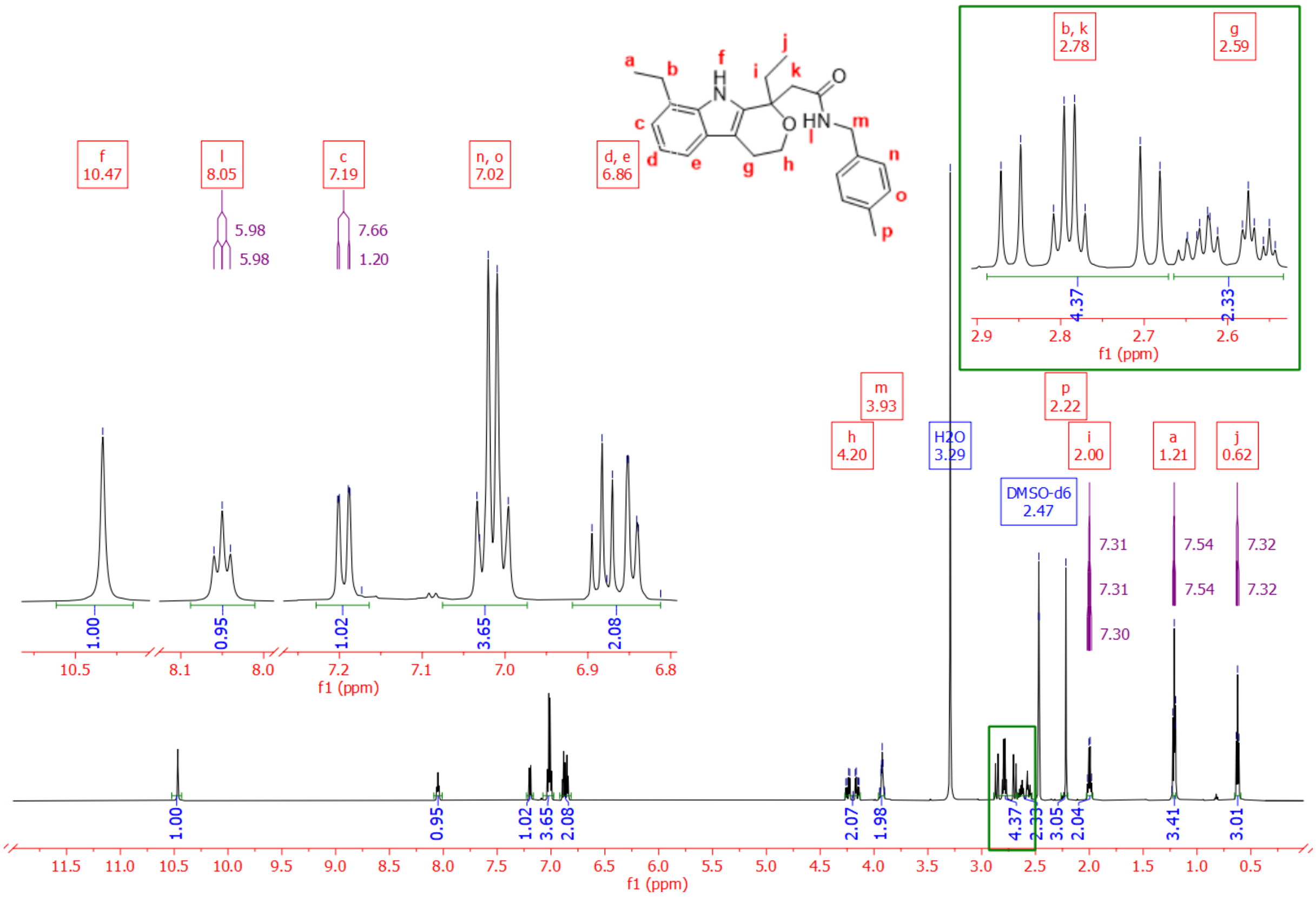

Figure S24. ^{1}H NMR Spectrum for 2-(1,8-diethyl-1,3,4,9-tetrahydropyrano[3,4-b]indol-1-yl)-*N*-(4-methylbenzyl)acetamide (**ECG**)

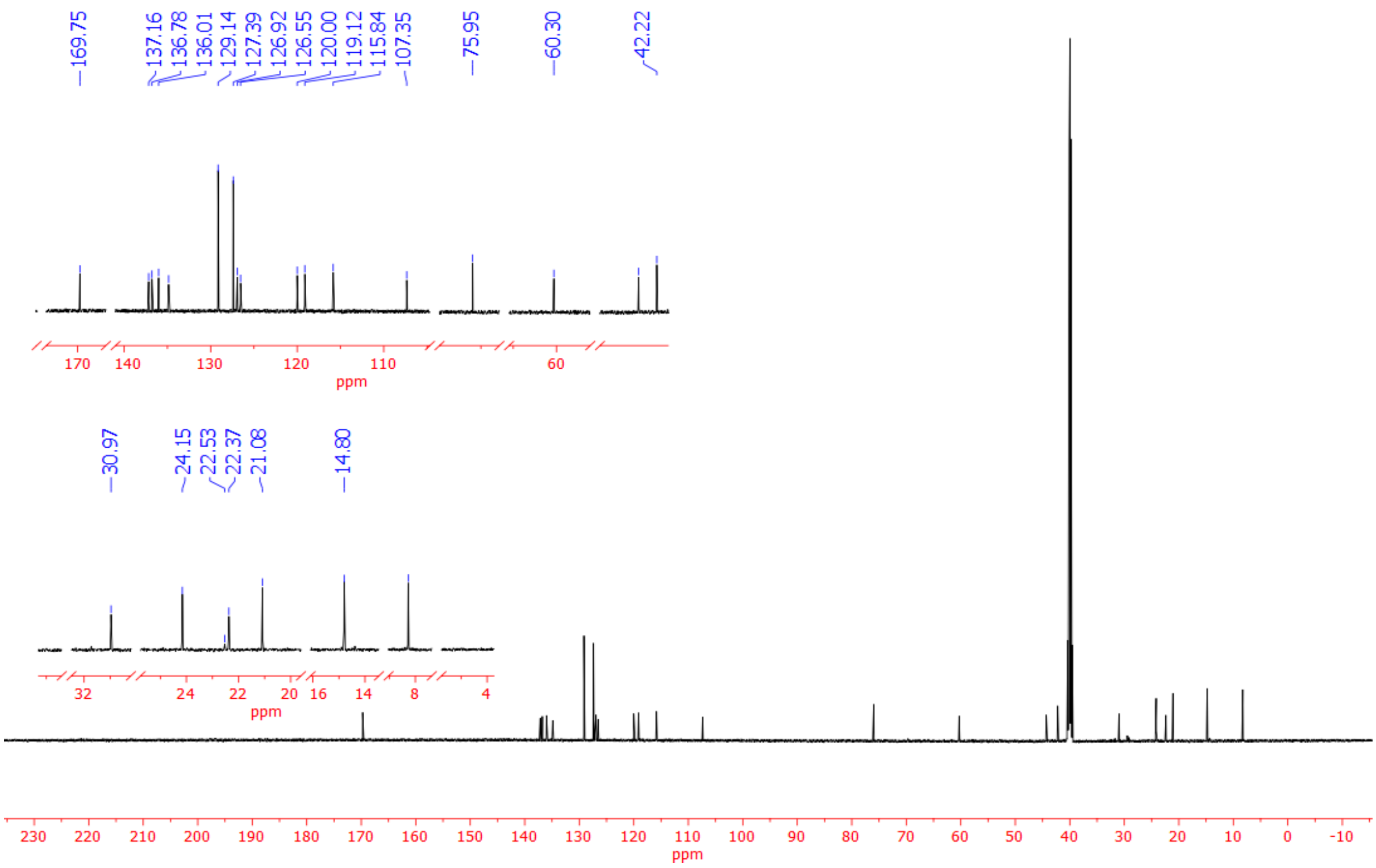

Figure S25. ^{13}C NMR Spectrum for 2-(1,8-diethyl-1,3,4,9-tetrahydropyrano[3,4-b]indol-1-yl)-*N*-(4-methylbenzyl)acetamide (**ECG**)

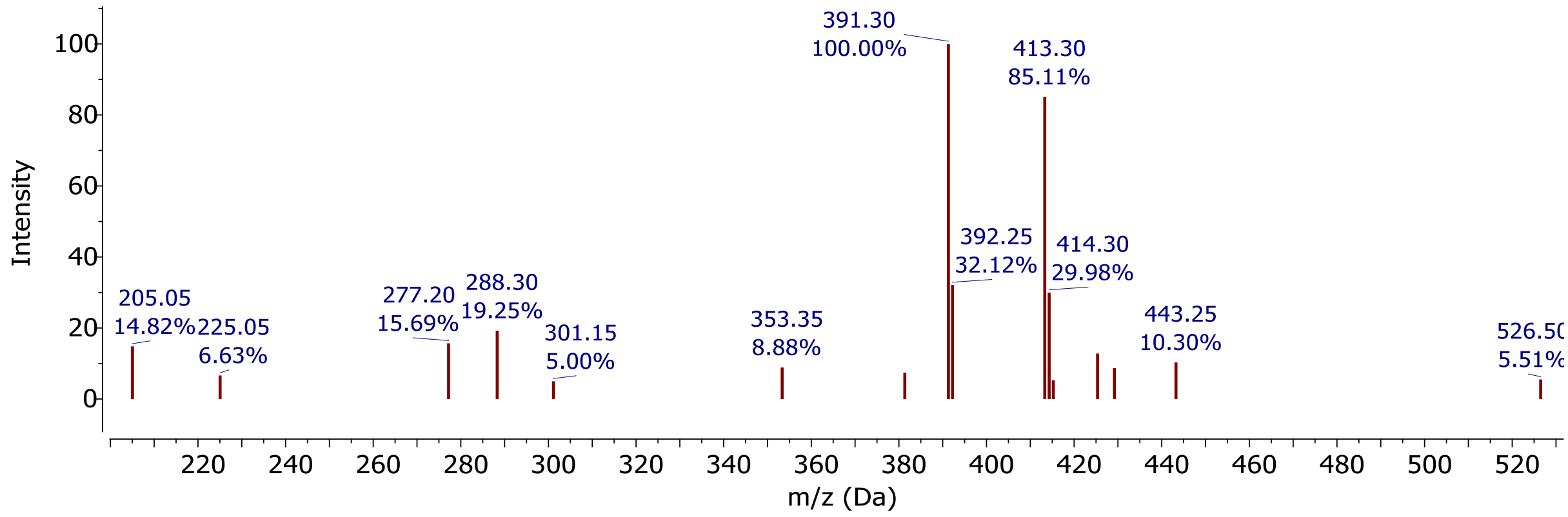

Figure S26. LC-MS/MS Spectrum for 2-(1,8-diethyl-1,3,4,9-tetrahydropyrano[3,4-b]indol-1-yl)-*N*-(4-methylbenzyl)acetamide (**ECG**)

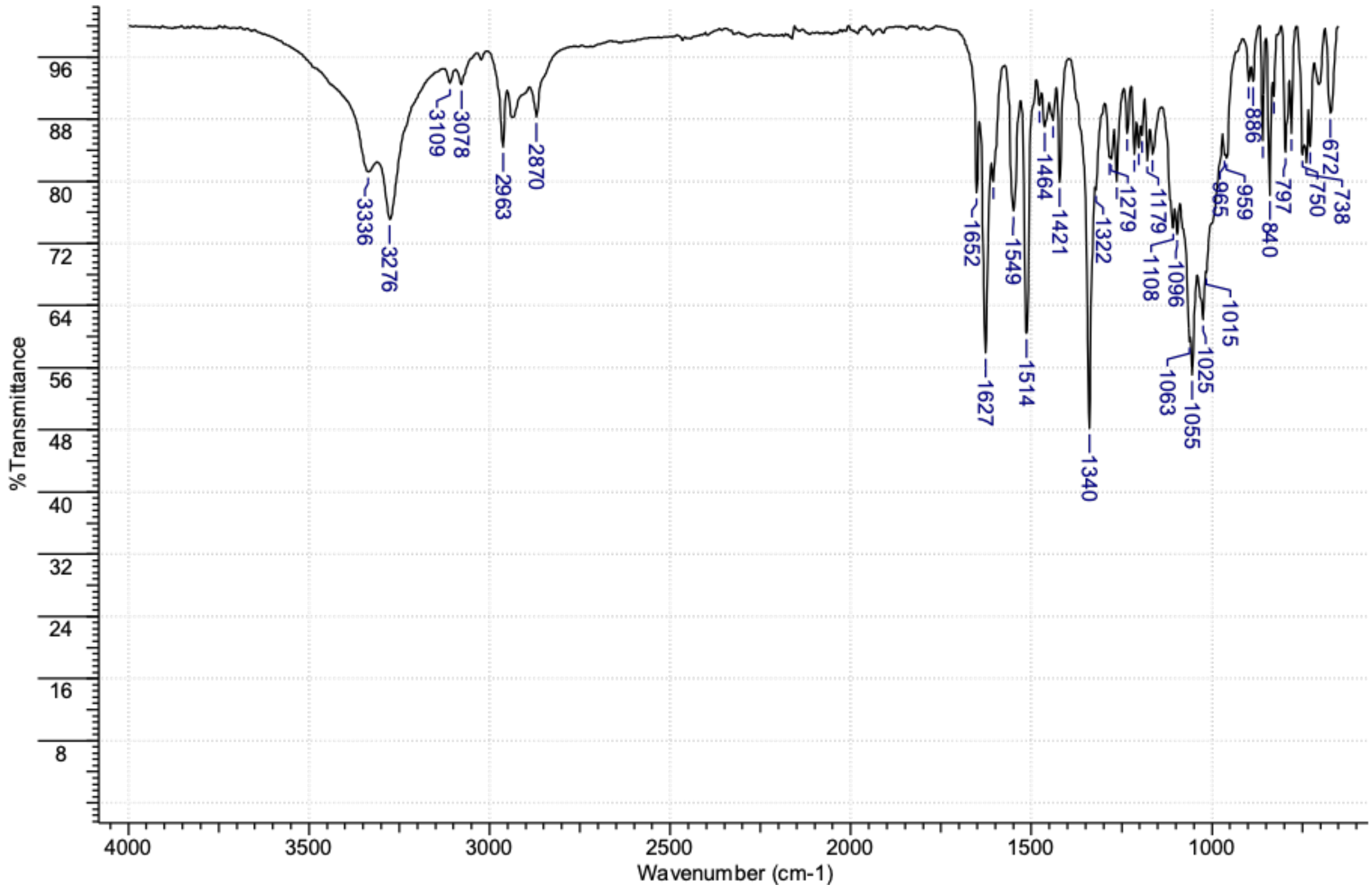

Figure S27. FTIR Spectrum for 2-(1,8-diethyl-1,3,4,9-tetrahydropyrano[3,4-b]indol-1-yl)-*N*-(4-nitrobenzyl)acetamide (**ECH**)

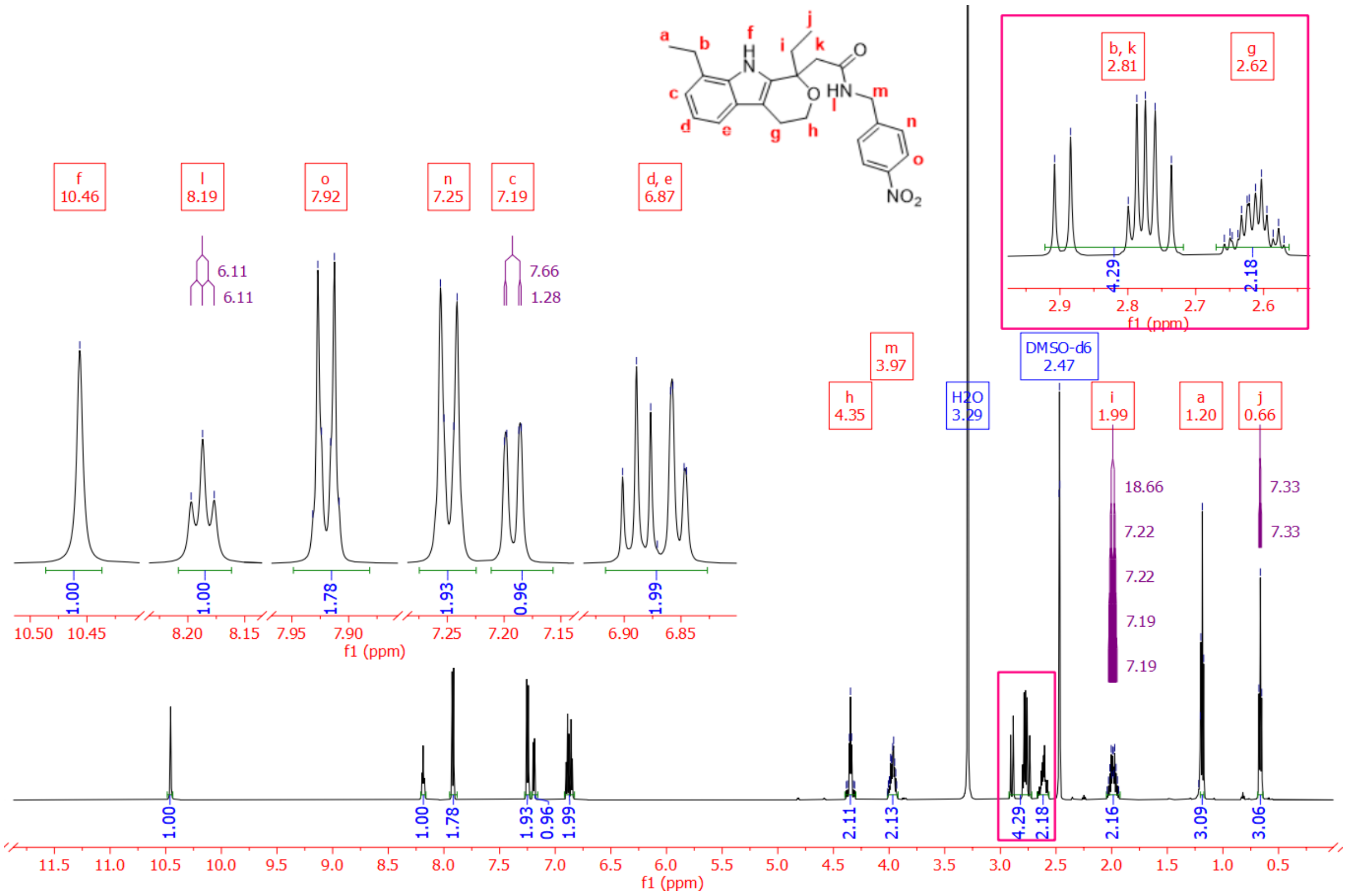

Figure S28. ^{1}H NMR Spectrum for 2-(1,8-diethyl-1,3,4,9-tetrahydropyrano[3,4-b]indol-1-yl)-*N*-(4-nitrobenzyl)acetamide (**ECH**)

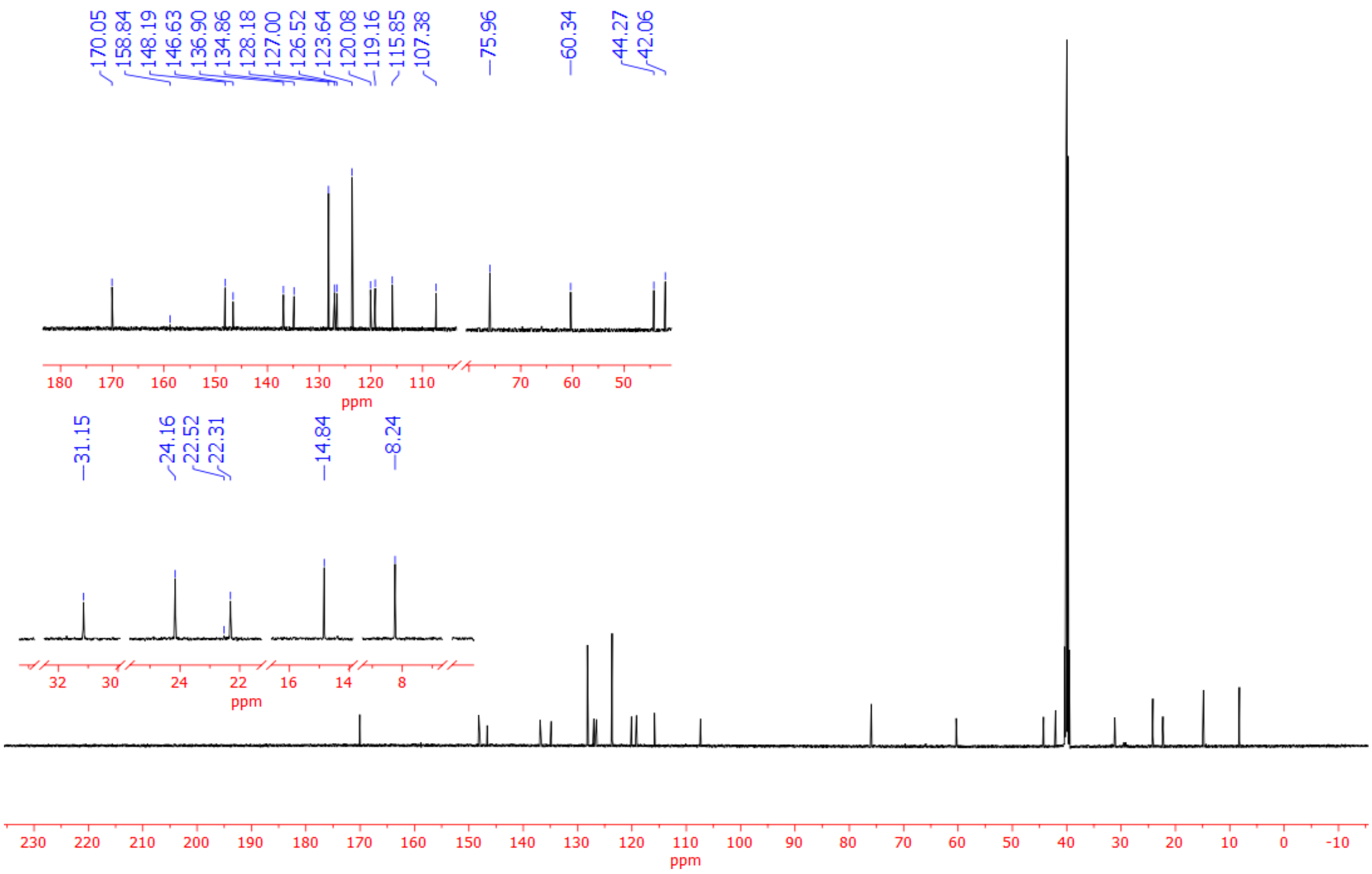

Figure S29. ^{13}C NMR Spectrum for 2-(1,8-diethyl-1,3,4,9-tetrahydropyrano[3,4-b]indol-1-yl)-*N*-(4-nitrobenzyl)acetamide (**ECH**)

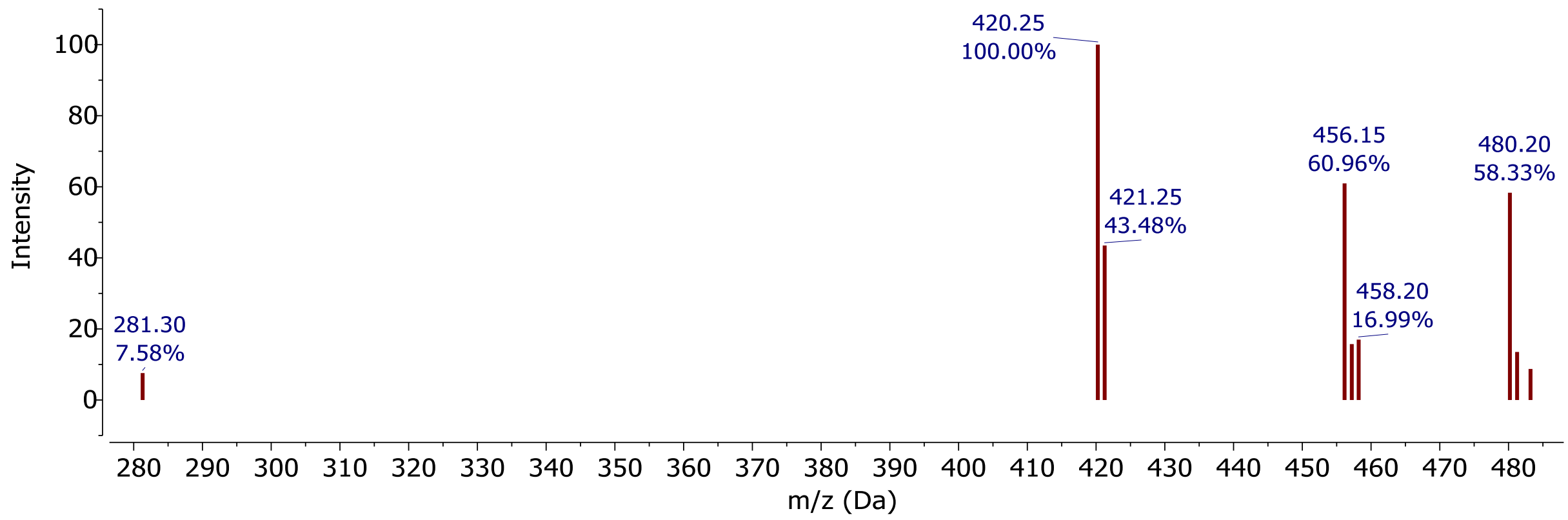

Figure S30. LC-MS/MS Spectrum for 2-(1,8-diethyl-1,3,4,9-tetrahydropyrano[3,4-b]indol-1-yl)-*N*-(4-nitrobenzyl)acetamide (**ECH**)

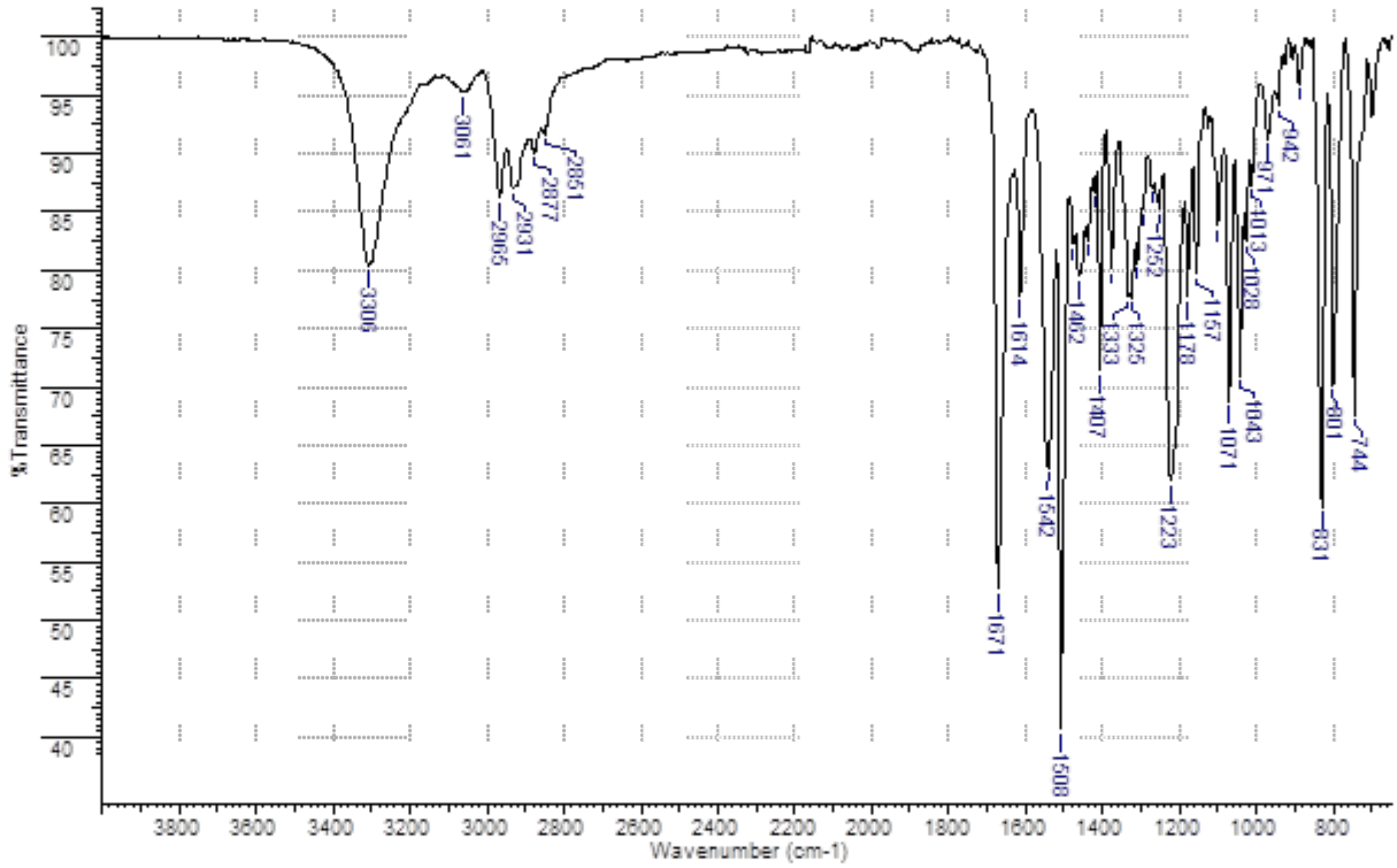

Figure S31. FTIR Spectrum for 2-(1,8-diethyl-1,3,4,9-tetrahydropyrano[3,4-b]indol-1-yl)-*N*-(4-fluorophenyl)acetamide (**ECK**)

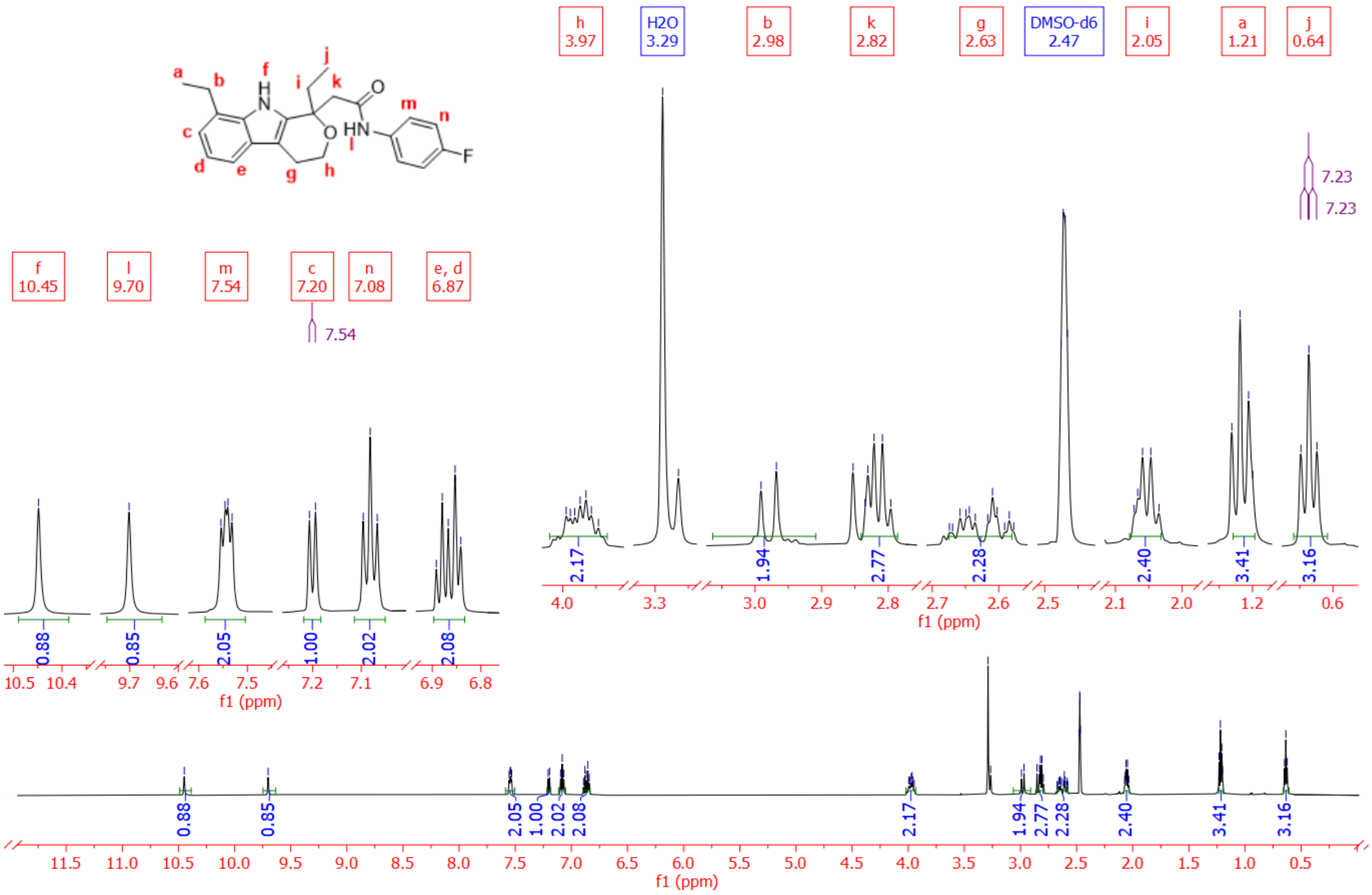

Figure S32. ^{1}H NMR Spectrum for 2-(1,8-diethyl-1,3,4,9-tetrahydropyrano[3,4-b]indol-1-yl)-*N*-(4-fluorophenyl)acetamide (**ECK**)

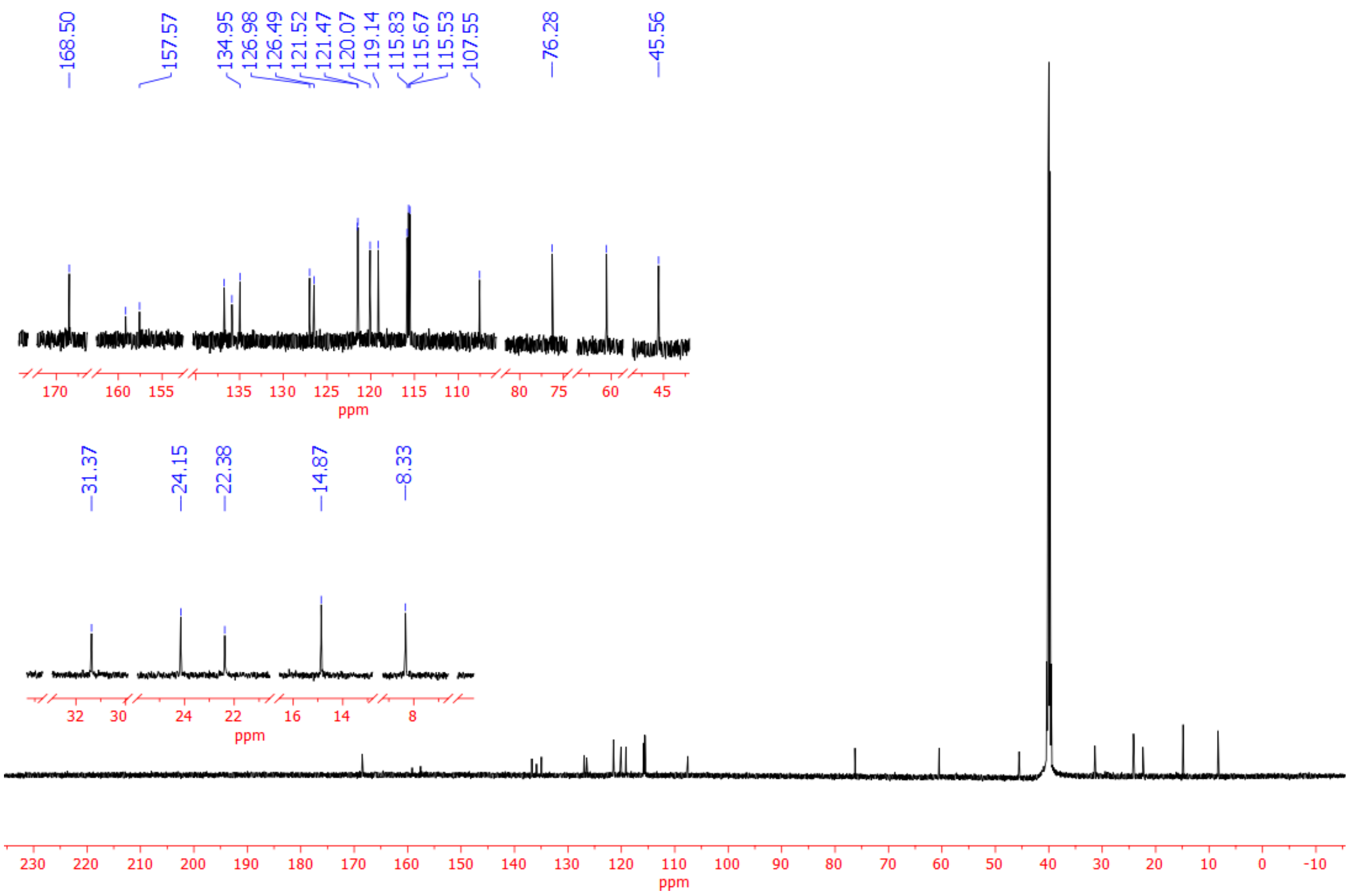

Figure S33. ^{13}C NMR Spectrum for 2-(1,8-diethyl-1,3,4,9-tetrahydropyrano[3,4-b]indol-1-yl)-*N*-(4-fluorophenyl)acetamide (**ECK**)

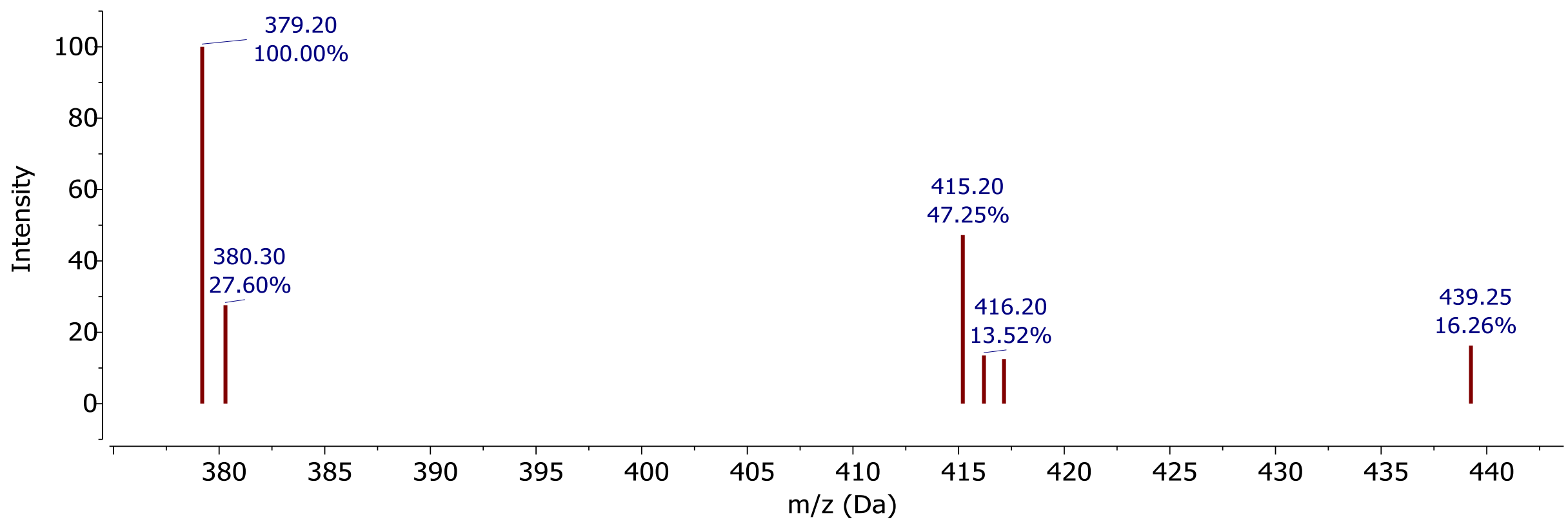

Figure S34. LC-MS/MS Spectrum for 2-(1,8-diethyl-1,3,4,9-tetrahydropyrano[3,4-b]indol-1-yl)-*N*-(4-fluorophenyl)acetamide (**ECK**)

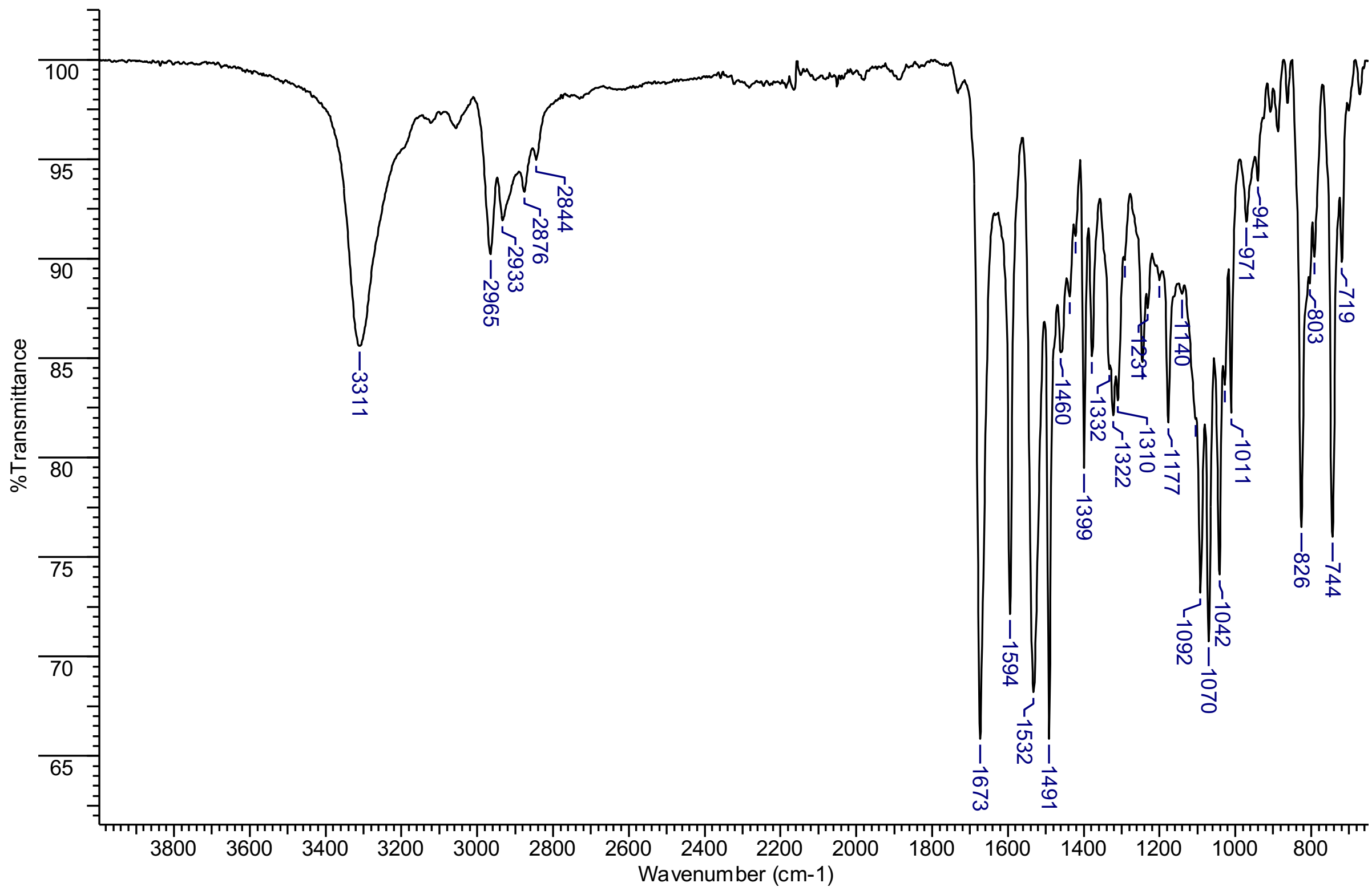

Figure S35. FTIR Spectrum for *N*-(4-chlorophenyl)-2-(1,8-diethyl-1,3,4,9-tetrahydropyrano[3,4-b]indol-1-yl)acetamide (**ECL**)

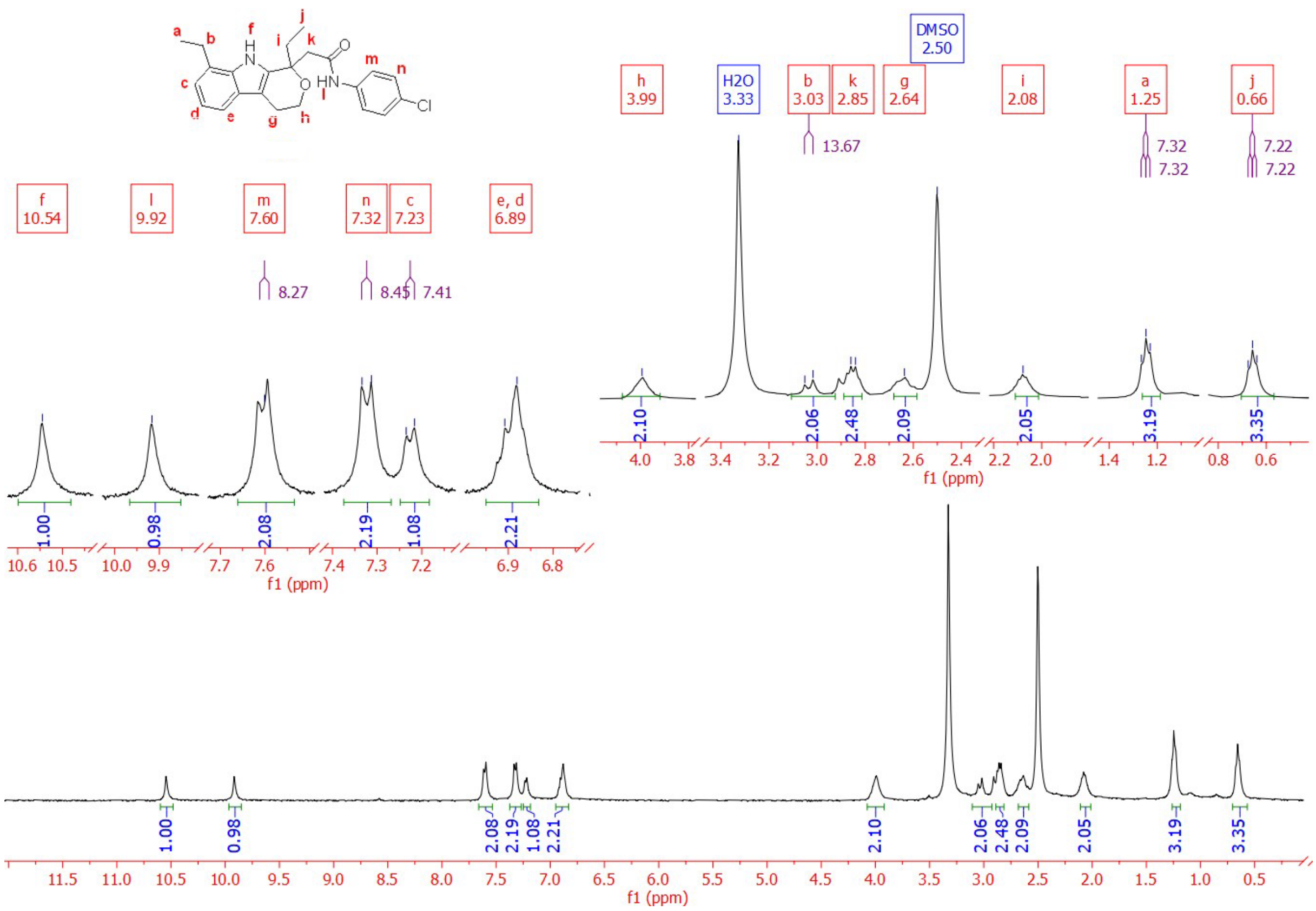

Figure S36. ^{1}H NMR Spectrum for *N*-(4-chlorophenyl)-2-(1,8-diethyl-1,3,4,9-tetrahydropyrano[3,4-b]indol-1-yl)acetamide (**ECL**)

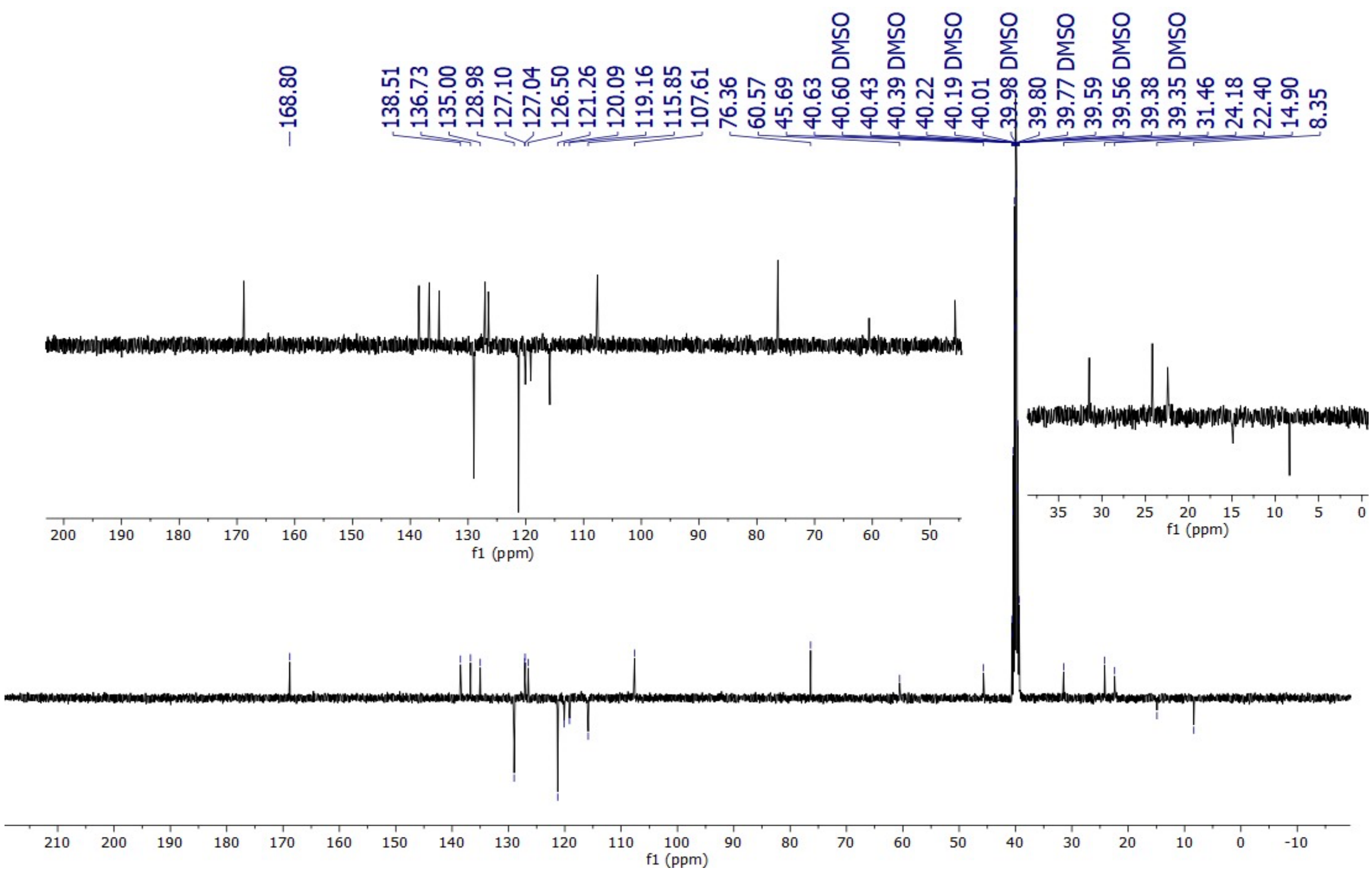

Figure S37. ^{13}C NMR (APT) Spectrum for *N*-(4-chlorophenyl)-2-(1,8-diethyl-1,3,4,9-tetrahydropyrano[3,4-b]indol-1-yl)acetamide (**ECL**)

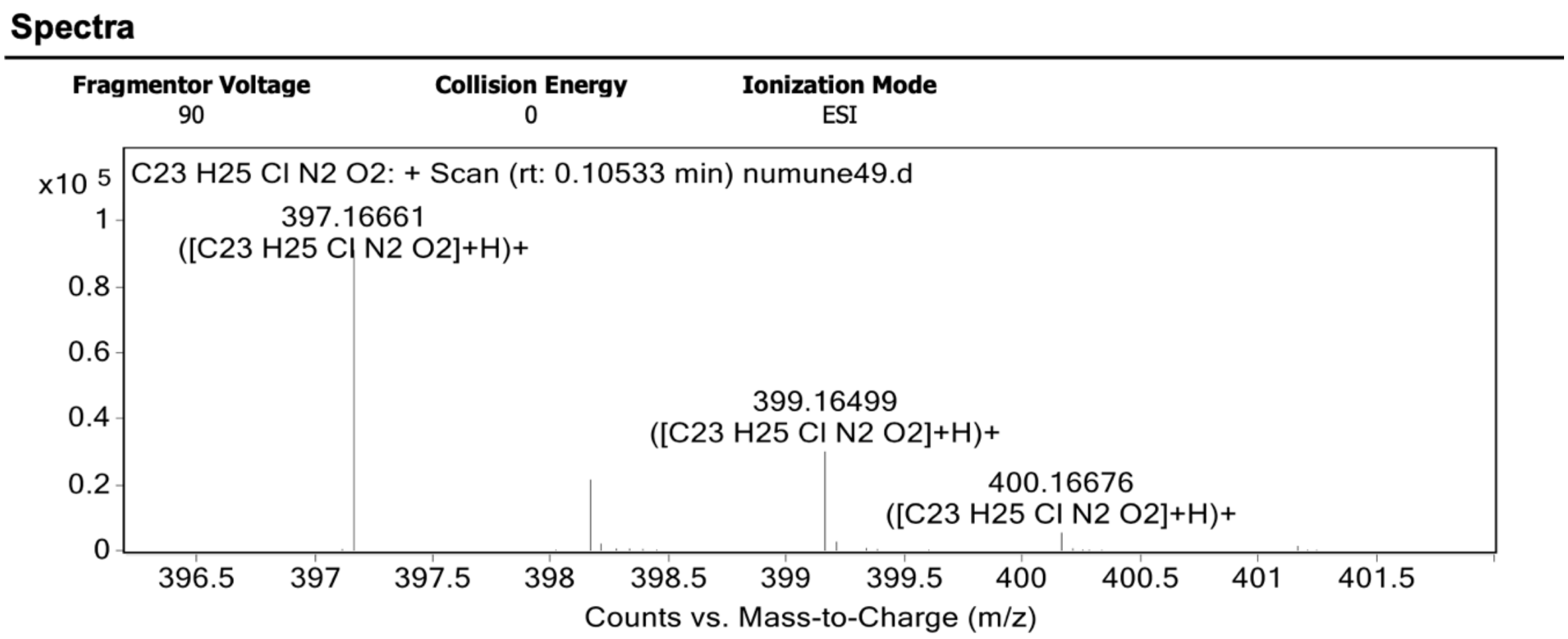

Figure S38. QTOF HRMS Spectrum for *N*-(4-chlorophenyl)-2-(1,8-diethyl-1,3,4,9-tetrahydropyrano[3,4-b]indol-1-yl)acetamide (**ECL**)

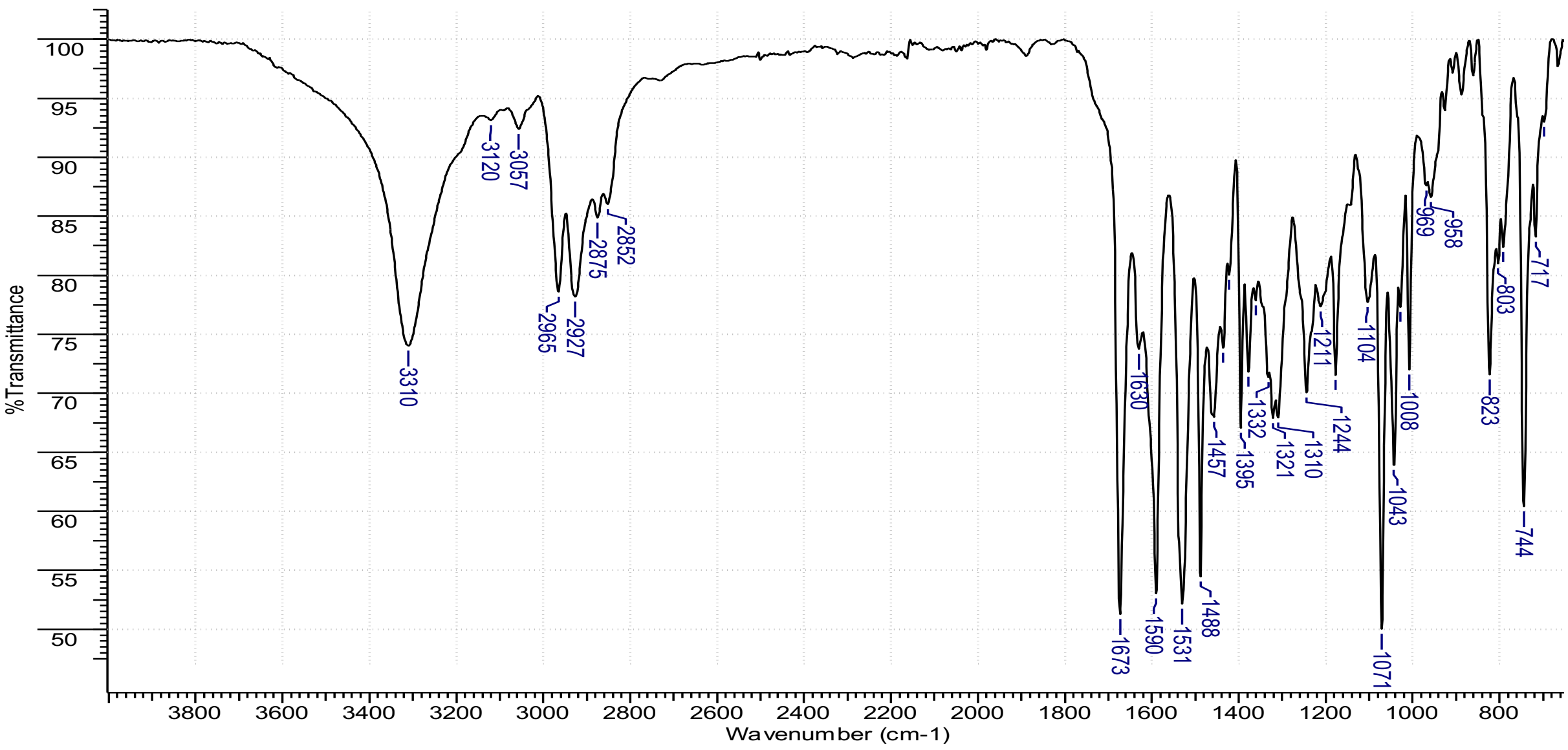

Figure S39. FTIR Spectrum for *N*-(4-bromophenyl)-2-(1,8-diethyl-1,3,4,9-tetrahydropyrano[3,4-b]indol-1-yl)acetamide (**ECM**)

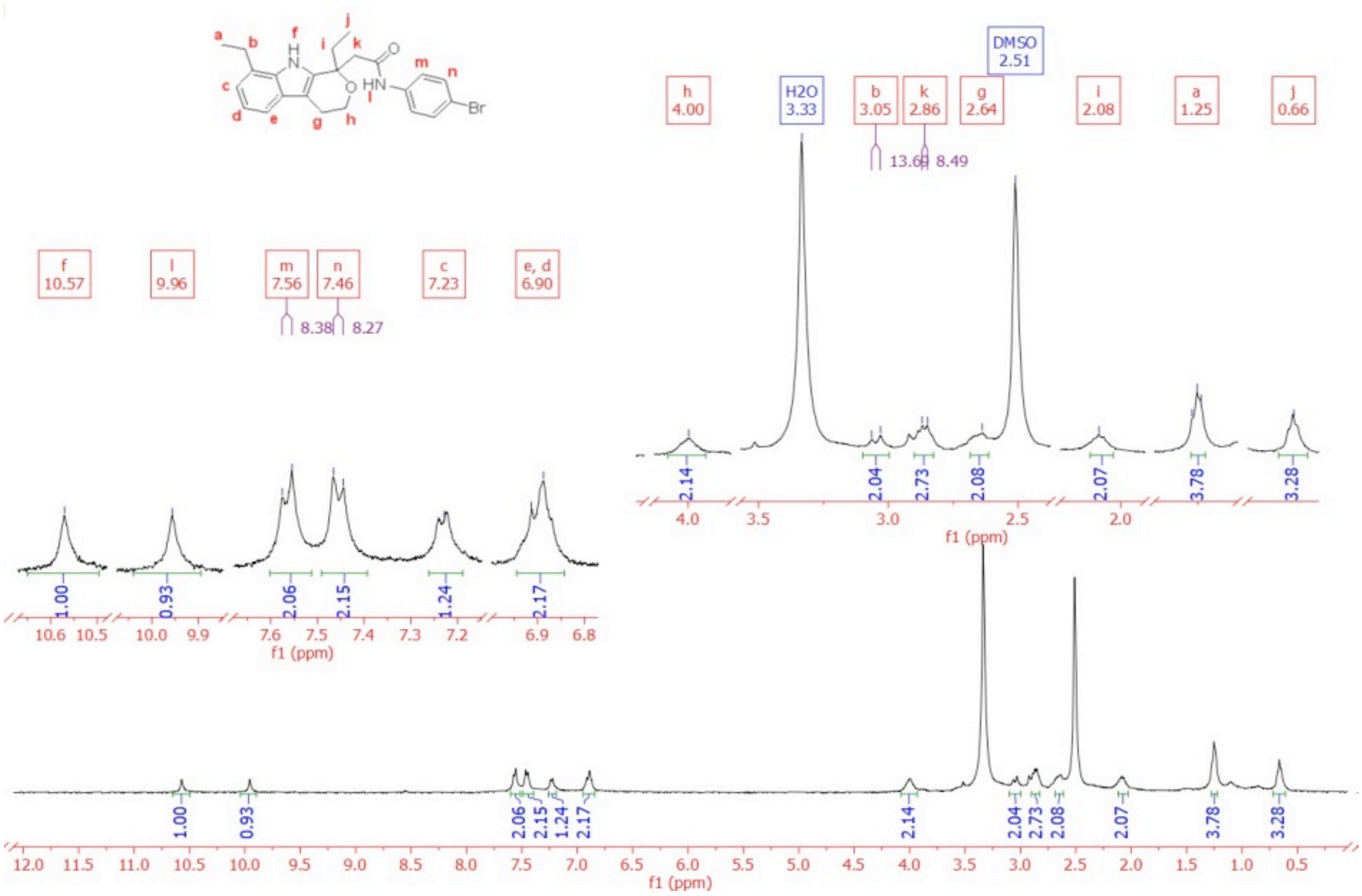

Figure S40. ^{1}H NMR Spectrum for *N*-(4-bromophenyl)-2-(1,8-diethyl-1,3,4,9-tetrahydropyrano[3,4-b]indol-1-yl)acetamide (**ECM**)

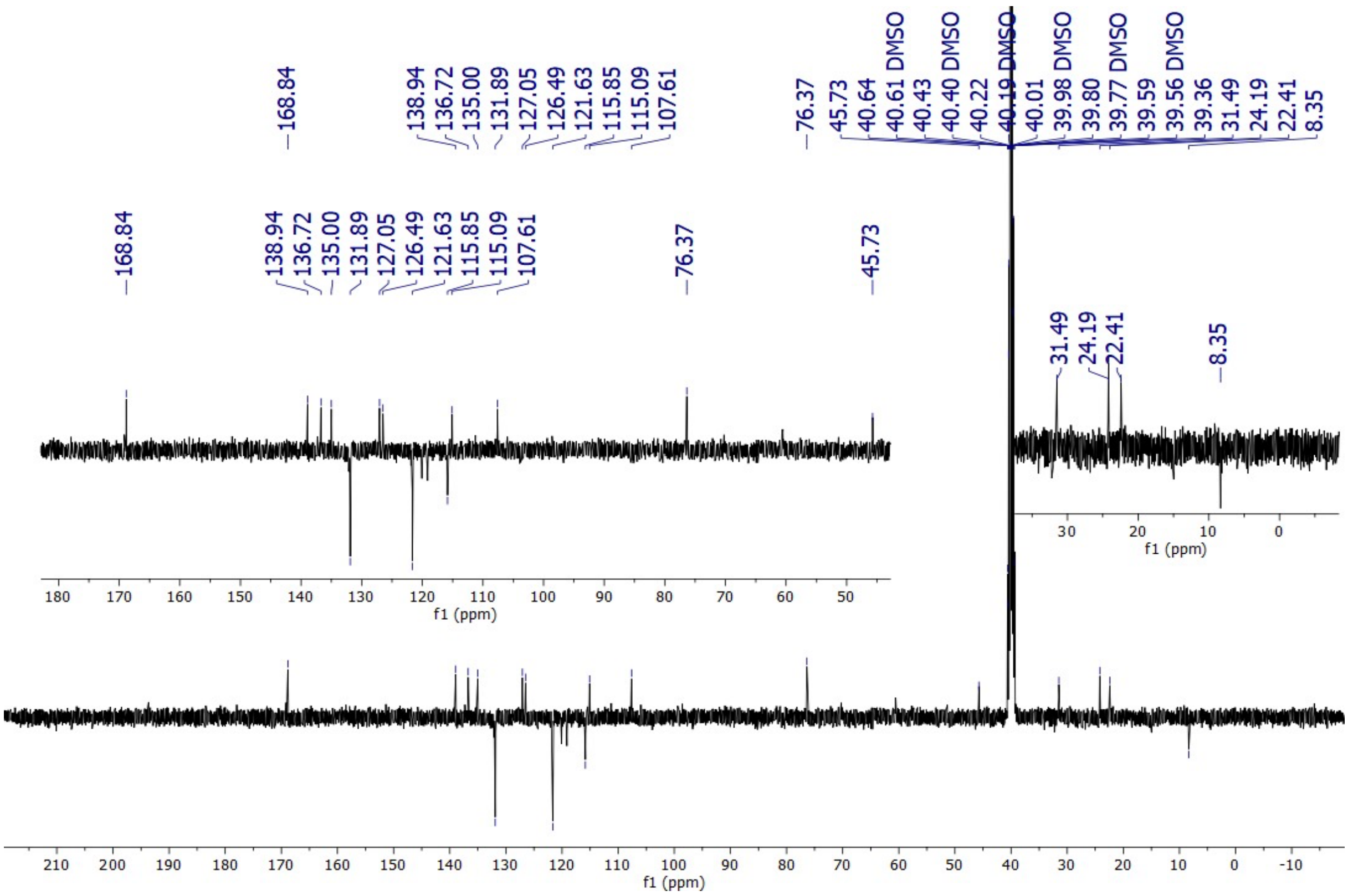

Figure S41. ^{13}C NMR **(APT)** Spectrum for *N*-(4-bromophenyl)-2-(1,8-diethyl-1,3,4,9-tetrahydropyrano[3,4-b]indol-1-yl)acetamide (**ECM**)

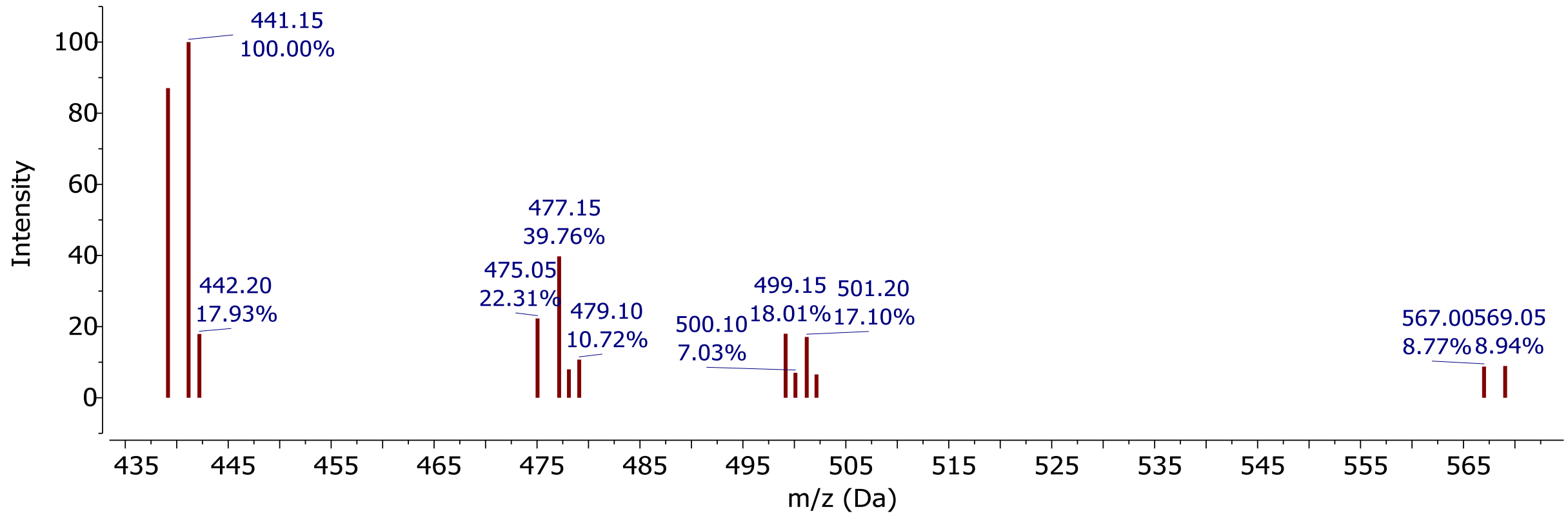

Figure S42. LC-MS/MS Spectrum for *N*-(4-bromophenyl)-2-(1,8-diethyl-1,3,4,9-tetrahydropyrano[3,4-b]indol-1-yl)acetamide (**ECM**)

Table S1. *In silico* toxicity prediction of the synthesized compounds (**ECA-ECM**).

Compounds	TOPKAT AMES Prediction	TOPKAT Mouse Female NTP Prediction	TOPKAT Mouse Male NTP Prediction	TOPKAT Rat Female NTP Prediction	TOPKAT Rat Male NTP Prediction	TOPKAT Mouse Female FDA	TOPKAT Mouse Male FDA
**ECA**	Non-Mutagen	Non-Carcinogen	Non-Carcinogen	Non-Carcinogen	Non-Carcinogen	Non-Carcinogen	Non-Carcinogen
**ECB**	Non-Mutagen	Non-Carcinogen	Non-Carcinogen	Non-Carcinogen	Non-Carcinogen	Non-Carcinogen	Non-Carcinogen
**ECC**	Non-Mutagen	Non-Carcinogen	Non-Carcinogen	Non-Carcinogen	Non-Carcinogen	Non-Carcinogen	Non-Carcinogen
**ECD**	Non-Mutagen	Non-Carcinogen	Non-Carcinogen	Non-Carcinogen	Non-Carcinogen	Non-Carcinogen	Non-Carcinogen
**ECE**	Non-Mutagen	Non-Carcinogen	Non-Carcinogen	Non-Carcinogen	Non-Carcinogen	Non-Carcinogen	Non-Carcinogen
**ECF**	Non-Mutagen	Non-Carcinogen	Non-Carcinogen	Non-Carcinogen	Non-Carcinogen	Non-Carcinogen	Non-Carcinogen
**ECG**	Non-Mutagen	Non-Carcinogen	Non-Carcinogen	Non-Carcinogen	Non-Carcinogen	Non-Carcinogen	Non-Carcinogen
**ECH**	Non-Mutagen	Non-Carcinogen	Non-Carcinogen	Carcinogen	Carcinogen	Non-Carcinogen	Non-Carcinogen
**ECK**	Non-Mutagen	Non-Carcinogen	Non-Carcinogen	Non-Carcinogen	Non-Carcinogen	Non-Carcinogen	Non-Carcinogen
**ECL**	Non-Mutagen	Non-Carcinogen	Non-Carcinogen	Non-Carcinogen	Non-Carcinogen	Non-Carcinogen	Non-Carcinogen
**ECM**	Non-Mutagen	Non-Carcinogen	Carcinogen	Non-Carcinogen	Non-Carcinogen	Non-Carcinogen	Non-Carcinogen
